# Repurposing MDM2 inhibitor RG7388 for *TP53*-mutant NSCLC: a p53-independent pyroptotic mechanism via ROS/p-p38/NOXA/caspase-3/GSDME axis

**DOI:** 10.1038/s41419-025-07770-2

**Published:** 2025-06-17

**Authors:** Gaoyan Tang, Xuelei Cao, Jiaqi Chen, Fu Hui, Na Xu, Yiqing Jiang, Hongmin Lu, Haifeng Xiao, Xiuming Liang, Mingzhe Ma, Yu Qian, Dongli Liu, Zhenlu Wang, Shuzhen Liu, Guohua Yu, Lei Sun

**Affiliations:** 1 Department of Oncology, Weifang People’s Hospital, the First Affiliated Hospital of Shandong Second Medical University, Weifang, China; 2 Department of Clinical Laboratory, Qilu Hospital of Shandong University, Jinan, China; 3 Shandong Engineering Research Center of Biomarker and Artificial Intelligence Application, Jinan, Shandong China; 4 Cancer Prevention and Treatment Center, The Second Hospital of Shandong University, Jinan, China; 5 Department of Oncology, Shanghai Renji Hospital, Shanghai Jiaotong University medical school, Shanghai, China; 6 Division for Biomolecular and Cellular Medicine, Department of Laboratory Medicine, Karolinska Institutet, Stockholm, Sweden; 7 Karolinska ATMP Center, ANA Futura, Karolinska Institutet, Stockholm, Sweden; 8 Department of Cellular Therapy and Allogeneic Stem Cell Transplantation (CAST), Karolinska University Hospital, Stockholm, Sweden; 9 Department of Gastric Surgery, Fudan University Shanghai Cancer Center, Shanghai, China; 10 Department of thoracic head and neck medical oncology, University of Texas MD Anderson Cancer Center, Houston, Texas USA; 11 Department of Radiation Oncology, Shanghai General Hospital, Shanghai Jiaotong University medical school, Shanghai, China

**Keywords:** Apoptosis, Non-small-cell lung cancer, Cancer therapeutic resistance

## Abstract

Non-small cell lung cancer (NSCLC) is highly malignant with limited treatment options, largely due to the inherent tumoral heterogeneity and acquired resistance towards chemotherapy and immunotherapy. RG7388, a known MDM2 inhibitor, exhibited anticancer activity in *TP53*-wild-type (*TP53*^WT^) NSCLC by triggering the p53/PUMA axis-dependent apoptosis. However, our study uncovered previously unrecognized p53-independent anticancer effects of RG7388 in *TP53*-mutant (*TP53*^mutant^) NSCLC, although the underlying mechanisms remained elusive. Here, we demonstrated that RG7388 specifically induced the NOXA/caspase-3 axis-dependent apoptosis and gasdermin E (GSDME)-mediated secondary pyroptosis in *TP53*^mutant^ NSCLC, as validated through in silico analyses and multiple biological assays. Mechanically, we identified reactive oxygen species (ROS) as the critical mediator in NOXA upregulation and p38 MAPK pathway activation in RG7388 treated *TP53*^mutant^ NSCLC. This was further supported by the use of ROS scavengers, N-acetylcysteine (NAC), and Ferrostatin-1 (Fer-1), which attenuated these effects. Pharmacologic inhibition of p38 MAPK signaling by SB203580 rescued RG7388-induced ROS-dependent NOXA accumulation and subsequent apoptosis and pyroptosis, highlighting the central role of the ROS/phosphorylated p38 MAPK (p-p38)/NOXA/caspase-3 axis in RG7388-induced *TP53*^mutant^ NSCLC cell death. Our findings revealed a novel mechanism for selectively targeting mutant p53-derived cancer through ROS/p-p38-mediated NOXA accumulation, offering potential therapeutic implications given the current lack of direct mutant p53 targeting strategies in cancer. Furthermore, immunohistochemical (IHC) analysis of an NSCLC tissue microarray confirmed a strong positive correlation between p-p38 and NOXA expression. Clinical data analysis further suggested that the p-p38/NOXA axis might be a potential prognostic biomarker for overall survival (OS) in NSCLC patients.

## Introduction

Lung cancer remains the most frequently diagnosed cancer and the leading cause of cancer-related deaths globally, with a dismal 5-year survival rate of <20% [1]. Among lung cancer subtypes, non-small cell lung cancer (NSCLC) constitutes approximately 85% of all cases, with adenocarcinomas representing nearly 40% of the histological types [2]. A significant proportion of NSCLC patients (approximately 50%) harbor genetic alterations in key oncogenes, including *EGFR*, *KRAS*, and *ALK*, which drive dysregulated cellular metabolism and sustained proliferative signaling [2–4]. In parallel, genomic changes in tumor suppressor genes such as *TP53* and *LKB1* are also frequently observed in NSCLC [4]. The *TP53* gene encodes p53, a tumor suppressor and master regulator of cellular stress responses that coordinates DNA repair, cell cycle arrest, and apoptosis [5]. Mutations in *TP53* in NSCLC patients are associated with more aggressive phenotypes, increased resistance to conventional chemotherapy, and poorer patient survival outcomes [5, 6]. Recent advances in targeted therapy have led to the development of novel small-molecule inhibitors that specifically target mutant p53. These inhibitors aim to mitigate the gain-of-function and dominant-negative effects of mutant p53 by promoting its degradation [5, 7]. Given the clinical challenges posed by *TP53* mutations, there is a pressing need to explore novel natural or synthetic compounds that can effectively target mutant p53 and improve therapeutic outcomes.

MDM2, an E3 ubiquitin ligase, is a key negative regulator of the tumor suppressor p53, promoting its ubiquitination and subsequent proteasomal degradation [8]. RG7388 (idasanutlin), a second-generation MDM2 inhibitor, selectively disrupts the MDM2-p53 interaction through high-affinity binding to the p53 pocket of MDM2, thereby restoring wild-type p53 activity and promoting apoptosis in *TP53*^WT^ cancers [9, 10]. Unlike first-generation inhibitors (Nutlin-3a and RG7112), RG7388 exhibits superior binding affinity and pharmacokinetic properties, demonstrating potent antitumor effects in preclinical models of hematologic malignancies and solid tumors [9, 11, 12]. However, its efficacy and underlying mechanisms in *TP53*mutant NSCLC, where p53-dependent apoptosis is compromised, remain poorly understood. Beyond apoptosis, emerging evidence from neuroblastoma studies demonstrates that RG7388 mediates p53-independent suppression of HIF-1α/VEGF-mediated angiogenesis [12]. Nevertheless, the molecular pathways governing alternative non-apoptotic cell death modalities, such as pyroptosis, remain to be fully elucidated. This gap highlights the critical need to investigate RG7388’s therapeutic potential in *TP53*^mutant^ NSCLC, where conventional therapies often fail due to chemoresistance.

Pyroptosis is a lytic, proinflammatory cell death modality executed by gasdermin proteins (GSDMs) following caspase activation. It is characterized by the formation of pores on the cell membrane mediated by GSDMs, leading to the loss of membrane integrity, increased permeability, and eventually cell membrane rupture, which triggers inflammatory reactions [13]. The most well-studied pyroptosis pathway is mediated by gasdermin D (GSDMD), which is activated by pro-inflammatory Caspases. Two distinct inflammatory pathways are involved in GSDMD-mediated pyroptosis: the canonical caspase-1/GSDMD and non-canonical caspase-4/5/11/GSDMD pathways [13, 14]. In addition to GSDMD-mediated pyroptosis, Gasdermin E (GSDME) can also induce pyroptosis when activated by pro-apoptotic caspase-3, a key executor of apoptosis, in an inflammasome-independent manner [14]. GSDME is a substrate of caspase-3, and its cleaved N-terminal fragments bind to membrane lipids and perforate the membrane, causing cell swelling and pyroptotic cell death [14]. This caspase-3/GSDME axis facilitates a switch from apoptosis to pyroptosis, particularly during chemotherapy [15, 16]. Pyroptosis has been increasingly recognized for its contribution to the anti-tumor properties of chemotherapy drugs [15–18]. Recent studies have highlighted that diverse small-molecule inhibitors can enhance therapeutic efficacy, tackle drug resistance and improve anti-tumor immunotherapy by activating the caspase-3/GSDME pathway-mediated pyroptosis [17–20]. Therefore, the development of strategies to activate GSDME-mediated pyroptosis is feasible for treating *TP53*^mutant^ refractory NSCLC. Furthermore, substantial evidence indicates that reactive oxygen species (ROS) plays a critical role in driving GSDME-mediated pyroptosis via mitochondrial oxidative damage, thereby exhibiting anti-tumor effects [17, 21–24]. These insights provide a potential therapeutic approach for cancer treatment by promoting mitochondrial oxidative damage-mediated pyroptosis.

Increasing evidence suggests that NOXA can serve as an important intracellular therapeutic target for inducing effective cancer cell death [25–28]. NOXA, a proapoptotic Bcl-2 homology 3 (BH3)-only member of the B-cell lymphoma 2 (BCL2) protein family, plays a pivotal role in the p53-dependent or p53-independent apoptosis [26, 28, 29]. Deletion of the *Phorbol-12-Myristate-13-Acetate-Induced Protein 1* (*PMAIP1*) gene encoding NOXA or the absence of NOXA expression has been shown to confer resistance to various apoptosis stimuli [25, 30]. During the process of apoptosis, NOXA translocates to the mitochondria, where it promotes permeabilization of the outer mitochondrial membrane, leading to cytochrome c release and subsequent activation of caspase proteins [31]. NOXA can be transcriptionally induced in various cellular stress conditions, such as hypoxia, DNA damage, or anti-cancer drugs, through the p53-dependent or p53-independent mechanisms [27, 28]. For example, excessive ROS can induce NOXA-mediated mitochondrial dysfunction, ultimately resulting in apoptosis [27, 32]. However, the precise mechanisms underlying the upregulation of NOXA by ROS have not yet been fully elucidated. Furthermore, extensive research has demonstrated that NOXA induction can prime cancer cells to apoptosis [25, 27, 30]. Despite its established role in apoptosis, the involvement of NOXA in pyroptosis has not been explored, to the best of our knowledge.

In this study, we identified the critical role of NOXA in RG7388-induced pyroptosis in *TP53*^mutant^ NSCLC. We were the first to report that RG7388, a novel MDM2 inhibitor, exhibited significant therapeutic potential in *TP53*^mutant^ NSCLC. Furthermore, we revealed that NOXA orchestrated the crosstalk between primary apoptosis and secondary pyroptosis triggered by RG7388 in *TP53*^mutant^ NSCLC cells. Additionally, we elucidated the underlying molecular mechanisms of NOXA-mediated cancer cell death, highlighting its potential as a novel therapeutic target for *TP53*^mutant^ NSCLC.

## Materials and methods

### Cell culture and regents

Human lung cancer cell lines including HCC827, NCI-H23, PC9, NCI-H1975, NCI-H3122, NCI-H2087, Calu-1, Calu-3, NCI-H1299, NCI-H358, NCI-H460 and A549 were obtained from ZQXZBIO, Shanghai, China. For lentivirus package, HEK293T cell was also obtained from ZQXZBIO. All cells were identified by short tandem repeat (STR) and tested for mycoplasma contamination. Cells were cultured in DMEM (C1199550013T, Gibco, USA) or RPMI-1640 (C1187550013T, Gibco, USA) supplement with fetal bovine serum (FBS; #04-001-1, Biological Industries, China), 100 U/mL penicillin and 100 U/mL streptomycin (A5873601, Gibco, USA) at 37 °C in a humidified atmosphere of 5% CO_2_.

RG7388 (T6254), SB203580 (T1764) and Ferrostatin-1 (T6500) were obtained from Target Mol (Boston, USA). RG7388 (HY-15676), Nutlin-3a (HY-10029), Z-VAD-FMK (HY-16658B), Z-IETD-FMK (HY-101297), Z-DEVD-FMK (HY-12466), SM-164 (HY-15989), SP600125 (HY-12041), Hexadimethrine bromide (HY-112735) and Acetylcysteine (HY-B0215) were obtained from MCE (NJ, USA). DAPI (C1002) and Propidium Iodide (ST511), was obtained from Beyotime (Shanghai, China). Puromycin (HY-B1743A) was obtained from Selleck (Houston, USA).

### Ethics statement and organoids culture

We collected NSCLC tissues for organoid culture via core needle biopsies. This study was approved by the Ethics Committee of Weifang People’s Hospital, Shandong Second Medical University (No. KYLL20240105-2) and was conducted in accordance with the principles of the Declaration of Helsinki. Written informed consent was obtained from all participants prior to tissue collection. NSCLC tissues were cut into 3-5 mm^3^ cubes and the isolated tissues were incubated in Advanced DMEM/F12 buffer (#12634-010, Gibco, USA) containing 5 mg/mL Collagenase II (C2-BIOC, Sigma-Aldrich, USA), 100 μg/mL DNase I (#10104159001, Roche, USA) and 10 μM Y27632 (S1049, Selleck, USA) on a shaker for 1-3 h at 37 °C, mechanical pipetting every 15 min, followed by filtration through 70 mm cell strainers (BS-70-CS, Bioshop, Hefei, China). The Red Blood Cell Lysis Buffer (B541001, Sangong Biotech, Shanghai, China) was used to remove erythrocytes. For organoid culture, 1×10^5^ cells were embedded in 20 μL ice-cold Matrigel (#356231, Corning, USA) and seeded into a 48-well tissue culture plate. Once Matrigel was solidified at 37 °C, 500 μL of pre-warmed BioGenous^TM^ Lung Organoid culture Medium (K2138-LA, BioGenous, Hangzhou, China) was added. For organoid passaging, NSCLC organoids were released in TrypLE^TM^ (#12605-028, Gibco, USA), and mechanical dissociation was performed every 5 min at 37 °C, followed by centrifugation at 300 g for 4 min. Ultimately, single cells were reseeded in Matrigel and cultured as described above. The organoid medium was refreshed every 2–3 d. After three passaging, Nutlin-3A was applied to establish a pure culture consisting of solid tumor organoids with *TP53* mutation [33].

### Cell viability and lactate dehydrogenase (LDH) cytotoxicity assays

Lung cancer cells were seeded into a 96-well plate. After treatment, the culture media were carefully discarded. Subsequently, the cells were incubated with fresh culture media containing 10% Cell Counting Kit-8 (CCK8) reagent (Dojindo, Kumamoto, Japan) at 37 °C for 30 min. Finally, the absorbance was measured at 450 nm using a SpectraMax^®^ Mini Multi-Mode Microplate Reader (Molecular devices, San Jose, CA, USA), and the cell viability was calculated. For LDH leakage analysis, cells were cultured in 96-well plates and then subjected to different treatments. After the cell culture supernatant was collected, LDH release was examined using the LDH-Glo^TM^ Cytotoxicity assay (J2380, Promega, USA) according to the manufacturer’s instruction. Cytotoxicity or mortality (%) = (Absorbance of sample - Absorbance of sample control wells) × (Absorbance of maximum cellular enzyme activity - Absorbance of sample control wells) × 1/2 × 100. All experiments were conducted in three independent experiments.

### Colony formation assay

A total of 0.2×10^3^ - 0.5×10^3^ cells per well were seeded evenly into six-well plates and incubated at 37 °C overnight. After treatment with the indicated inhibitor(s) for 6 h, the medium was discharged and cells were cultured with fresh medium. The cell culture medium was changed every 3 days. After 2 weeks of culture, the culture medium was removed, and the colonies were fixed in 4% paraformaldehyde (PFA; P0099, Beyotime, Shanghai, China) for 15 min, and then stained with 0.1% crystal violet solution (E607309, Sangong Biotech, Shanghai, China) at room temperature for 15 min.

### Caspase-3 activity assay

Caspase-3 activity in this study was detected by using a GreenNuc™ Caspase-3 activity kit (C1168, Beyotime, Shanghai, China) according to manufacturer’s instructions. Briefly, cells were incubated with 5 μM GreenNuc™ Caspase-3 substrate for 30 min and then the nuclei were stained with DAPI for 10 min. The images were photographed with a fluorescence microscope (IX73, Olympus, Japan).

### Propidium iodide (PI) staining

PI staining kit (ST1569, Beyotime, Shanghai, China) was used to evaluate pyroptotic cell death. HCC827 or PC9 cells were pretreated with SB203580 or not, and then treated with RG7388. Cells were then washed thrice with PBS and incubated with the PI buffer for 30 min at 37 °C. Subsequently, cells were washed thrice with PBS and photographed using a fluorescence microscope (IX73, Olympus, Japan).

### Flow cytometric analysis

A total of 1×10^6^ cells were seeded and treated with the indicated inhibitors. Cells were harvested, washed with cold PBS, and stained with the FITC-labeled Annexin V and PI using the Annexin V-FITC/PI Apoptosis Kit (AT101, Multi-Sciences Biotech, Hangzhou, China). Data was obtained using BD FACSMelody™ Cell Sorter and analyzed by BD FACSChorus™ software (BD Biosciences, USA). PI-positive cells were considered pyroptotic cells.

### Measurement of mitochondrial ROS

The ROS levels and mitochondria in cancer cells were detected with the DCFH-DA Detection Kit (S0033, Beyotime, Shanghai, China) and Mito-Tracker Red CMXRos kit (C1035, Beyotime, Shanghai, China), respectively. Briefly, cancer cells were seeded in a 12-well plate and incubated with RG7388 for 6 h. After washing, the cells were stained with 10 μM of DCFH-DA (blue) and 20 nM of Mito-Tracker buffer (red) at 37 °C for 30 min according to the manufacturer’s instructions. Subsequently, cells were washed with PBS three times and then imaged with a fluorescence microscope (IX73, Olympus, Japan).

### RNA isolation and real-time quantitative reverse transcription PCR (qRT-PCR)

As directed by the manufacturer, total RNA was extracted using RNA Isolation Kit (SB-R001, Sharebio, shanghai, China). After quantification, 1 μg total RNA was reverse transcribed to complementary DNA (SB-RT001, Sharebio, Shanghai, China). Transcript levels of indicated genes were quantified by qPCR on an Applied Biosystems QuantStudio^TM^ 5 Real-time PCR system (Thermo Fisher Scientific, USA) with SYBR Green (SB-Q204; Sharebio, Shanghai, China). The relative expression of mRNA was quantified by comparing the cycle threshold method (2^-ΔΔCt^). The sequences of primers were listed in Supplementary Table S1.

### Cytokine ELISA

The following ELISA kits, which were purchased from Multi-Sciences Biotech, Hangzhou, China, were used to determine supernatant levels of the following interleukins according to the manufacturer’s instructions: Human IL-1α ELISA kit (EK101A), Human IL-1β ELISA kit (EK101B), Human IL-6 ELISA kit (EK106) and Human IL-8 ELISA kit (EK108). All experiments were conducted in three independent experiments.

### Immunoblotting analysis

Whole protein lysates from lung cancer cells were extracted using RIPA lysis buffer (C500005, Sangong Biotech, Shanghai, China) containing protease and phosphatase inhibitor cocktail (#78443, Thermo Fisher Scientific, USA). Protein concentrations were assessed with the bicinchoninic acid (BCA) Protein Quantification kit (P0010, Beyotime, Shanghai, China). Cell lysates (25 μg/lane) were separated using 6%-15% SDS-PAGE, transferred into a nitrocellulose membrane (#10600001, Cytiva, USA), and then blocked for 1.5 h with 5% fat-free dry milk (A600669, Sangong Biotech, Shanghai, China) in 1× Tris-buffered saline (TBS; B548105, Sangong Biotech, Shanghai, China) with 0.1% Tween-20 (A600560, Sangong Biotech, Shanghai, China). Membranes were immunoblotted with primary antibodies on a shaker at 4 °C overnight. After washing with TBST, membranes were blotted with horseradish peroxidase (HRP)-conjugated anti-rabbit IgG (1:3000 dilution) or anti-mouse IgG (1:2000 dilution) antibodies for 2 h at room temperature. Blots were visualized by a 4800 Multi automatic Chemiluminescent Imaging system (Tanon, Shanghai, China).

Primary antibodies that recognized EGFR (sc-373746, Santa Cruz), p-EGFR Tyr1068 (#3777, Cell Signaling Technology), p-SHP2 Tyr542 (#15543, Cell Signaling Technology), p-MEK1/2 Ser217/221 (#9154, Cell Signaling Technology), p-ERK1/2 Thr185/Tyr187 (#44-680 G, Invitrogen), p-AKT Ser473 (#4060, Cell Signaling Technology), p-mTOR Ser2448 (#5536, Cell Signaling Technology), p-p70 S6 Kinase Thr421/Ser424 (#9204, Cell Signaling Technology), p-S6 Ribosomal Protein Ser235/236 (#4858, Cell Signaling Technology), p-p38 MAPK Tyr 182 (sc-166182, Santa Cruz), p-SAPK/JNK Thr183/Tyr185 (#4668, Cell Signaling Technology), Cleaved Caspase-3 (#9661, Cell Signaling Technology), Cleaved Caspase-7 (#8438, Cell Signaling Technology), Cleaved Caspase-8 (#9496, Cell Signaling Technology), Cleaved PARP (#9541, Cell Signaling Technology), Gasdermin E (#84005, Cell Signaling Technology), p53 (sc-126, Santa Cruz), NOXA (sc-56169, Santa Cruz), G3BP1 (sc-365338, Santa Cruz), G3BP2 (#16276-1-AP, Proteintech, USA), PD-L1/CD274 (#66248-1-Ig, Proteintech, USA), β-actin (MA5-11869, Invitrogen), GAPDH (#5174, Cell Signaling Technology) were used. Goat Anti-Rabbit IgG HRP-linked (#7074, Cell Signaling Technology) and Horse Anti-Mouse IgG HRP-linked (#7076, Cell Signaling Technology) were used as the secondary antibodies. The protein bands were quantified using ImageJ Version 1.54p (National Institutes of Health, USA).

### Immunofluorescent (IF)

Cells were fixed with 4% PFA for 15 min at room temperature and then permeated with 0.5% Triton X-100 for 15 min at room temperature. After blocking with 5% bovine serum albumin (BSA; Solarbio, Beijing, China) for 1 h at room temperature, the cells were incubated with the primary antibody of NOXA (sc-56169, Santa Cruz, USA) diluted at 1:200 overnight at 4 °C, and then incubated with the Goat anti-Mouse IgG (H + L) Cross-Adsorbed Secondary Antibody (A-11005, Invitrogen, USA) diluted at 1:1000 for 2 h at room temperature and protected from light. The nuclei were stained with DAPI (C1002, Beyotime, Shanghai, China) according to the manufacturer’s instructions. The images were photographed with a fluorescence microscope (IX73, Olympus, Japan).

### Small interfering (siRNA) transfection

For siRNA transfection, cells were plated at 60%–80% confluence and transfected with indicated siRNA duplex using Lipofectamine^TM^ 2000 Transfection Reagent (#11668019, Invitrogen) according to the manufacturer’s instructions. The efficacy of the siRNA knockdown was determined using western blot analysis. siRNAs were ordered from Sangon Biotech or Genepharma company, Shanghai, China. The sequences were as follows: siRNA_Negative Control (NC): UUCUCCGAACGUGUCACGUTT- 3’; siRNA_NOXA_1: 5’-CAGGAACCUGACUGCAUCAAATT- 3’; siRNA_NOXA_2: 5’-CUCAGGAGAUUUGGAGACAAATT- 3’; siRNA_NOXA_3: 5’-GCAGAAACUUCUGAAUCUGAUTT- 3’; siRNA_G3BP1_1: 5’-GGGAAUUUGUGAGACAGUATT- 3’; siRNA_G3BP1_2: 5’-GCCUGAGCCAGUAUUAGAATT- 3’; siRNA_G3BP2_1: 5’-CCAGAAAGAAAGUUUAUGCAATT- 3’

siRNA_G3BP2_2: 5’-CGCAUCAAUACCAAGGGUGUUTT- 3’; siRNA_p53_1: 5’-CCACCAUCCACUACAACUATT- 3’; siRNA_p53_2: 5’-GCACAGAGGAAGAGAAUCUTT- 3’.

### Generation of stable knockout cell lines using CRISPR–Cas 9 gene editing

Lenti-CRISPRv2 (#52961), pSPAX2 (#12260) and pMD2.G (#12259) were purchased from Addgene, USA. For construction of knockout cell lines, the following sgRNAs targeting *PMAIP1* (NOXA), *G3BP1* and *G3BP2* were used: Control sgRNA: CACCGGACCGGAACGATCTCGCGTA; *PMAIP1* sgRNA_1: *PMAIP1* sgRNA_2: CACCGACGCTCAACCGAGCCCCGCG; *PMAIP1* sgRNA_1: CACCGGGTTCCTGAGCAGAAGAGTT; *G3BP1* sgRNA_1: CACCGAGAGTCTGAAGAAGAAGTAG; *G3BP1* sgRNA_2: CACCGAATTCCCGCCCGACCAGCAG; *G3BP2* sgRNA_1: CACCGAGAGCTGAAACCACAAGTGG; *G3BP2* sgRNA_2: CACCGGAAACAGGTTCTGCCGGAGG. These sgRNAs were cloned into the lenti-CRISPRv2 vector plasmid and transfected into HEK-293T cells together with the packing plasmids psPAX2 and pMD2.G After 48 h, these sgRNAs containing lentivirus was collected. Stable knockout cells were generated by transduction with the lentivirus and subsequent puromycin selection for 7 days. Knockout efficiencies were determined by western blotting.

### Cell morphology

Cells were seeded in 12-well plates and treated with the indicated inhibitors. Micrographs of cells were obtained under phase contrast illumination using a 4×, 10× or 20× objective (CKX53, Olympus, Japan). All image data shown were representative of at least three randomly selected fields.

### RNA-seq analysis

Total RNA was isolated using RNeasy mini kit (#74104, Qiagen, Germany), after which RNA samples were quantified using the Qubit^®^ 3.0 Fluorometer (Life Technologies, CA, USA), and RNA integrity was assessed using the Agilent 2100 Bioanalyzer (Agilent Technologies Inc., USA). RNA-seq libraries were prepared using the Stranded mRNA-seq Lib Prep Kit for lllumina (ABclonal, China) following Preparation Guide. Deep sequencing of the libraries was performed on an Illumina NovaSeq 6000 (Illumina, USA). The RNA library construction and RNA-seq analysis was performed by Sinotech Genomics Co., Ltd (Shanghai, China). The RNA-seq raw data was available at GEO (GSE287938).

### Tissue microarray (TMA) analysis

TMA slides from patients at different NSCLC stages (#HLugA180Su12) were purchased from Superchip Company (Shanghai, China). IHC was performed using the REAL EnVision Detection System (k500711, Dako, Denmark) according to the manufacturer’s instructions, and hematoxylin (G1120HE, Sarlabio, Beijing, China) was used for counterstaining. Images were obtained by using an Aperio Scanner System (Leica, German) at a magnification of 400×. The staining intensity in all sections was assessed using the H-score independently by two pathologists following a blinded manner, and a consensus was reached to establish the final outcome. Patients were stratified into low and high expression groups based on the relative protein expression compared to corresponding normal tissue, as determined by IHC quantification of TMA samples. Comprehensive clinicopathological metadata and preprocessed bioinformatics profiling data for the cohort were obtained from the manufacturer’s database (Superchip Company, Shanghai, China). Multivariable Cox regression analysis was employed to assess the independent prognostic value of p-p38 and NOXA expression levels, with adjustment for tumor stage, histological grade, and maximum tumor diameter. The proportional hazards assumption was confirmed through Global Schoenfeld residuals test, with statistical validation performed by the provider’s certified biostatisticians.

### Transmission electron microscopy (TEM)

Following fixation with 2.5% glutaraldehyde, cellular specimens were processed through three sequential steps: ultrathin sectioning (50-100 nm thickness), double staining with uranyl acetate and lead citrate, and final stabilization on copper grids. Ultrastructural morphology, particularly focusing on plasma membrane integrity and mitochondrial architecture, was then examined using a Hitachi HT7700 TEM operating at 80 kV accelerating voltage (Hitachi High-Technologies, Tokyo, Japan).

### Statistical analysis

All data were represented as means ± standard error of the mean (SEM) or standard deviation (SD) and analyzed by using Graphpad prism 9 (GraphPad Software Inc., La Jolla, CA, USA). To calculate the significance of differences between the two sample groups, we performed analysis using the Student’s *t*-*test.* One way ANOVA was utilized to calculate the significance of differences among multiple groups. Kaplan–Meier survival curves were generated and compared using the log-rank test. Differences were considered to be statistically significant at *P* < 0.05.

## Results

### RG7388 suppressed cell proliferation, induced cell death and inhibited EGFR signaling pathway in *TP53*^mutant^ NSCLC cell lines

**Fig. 1:**
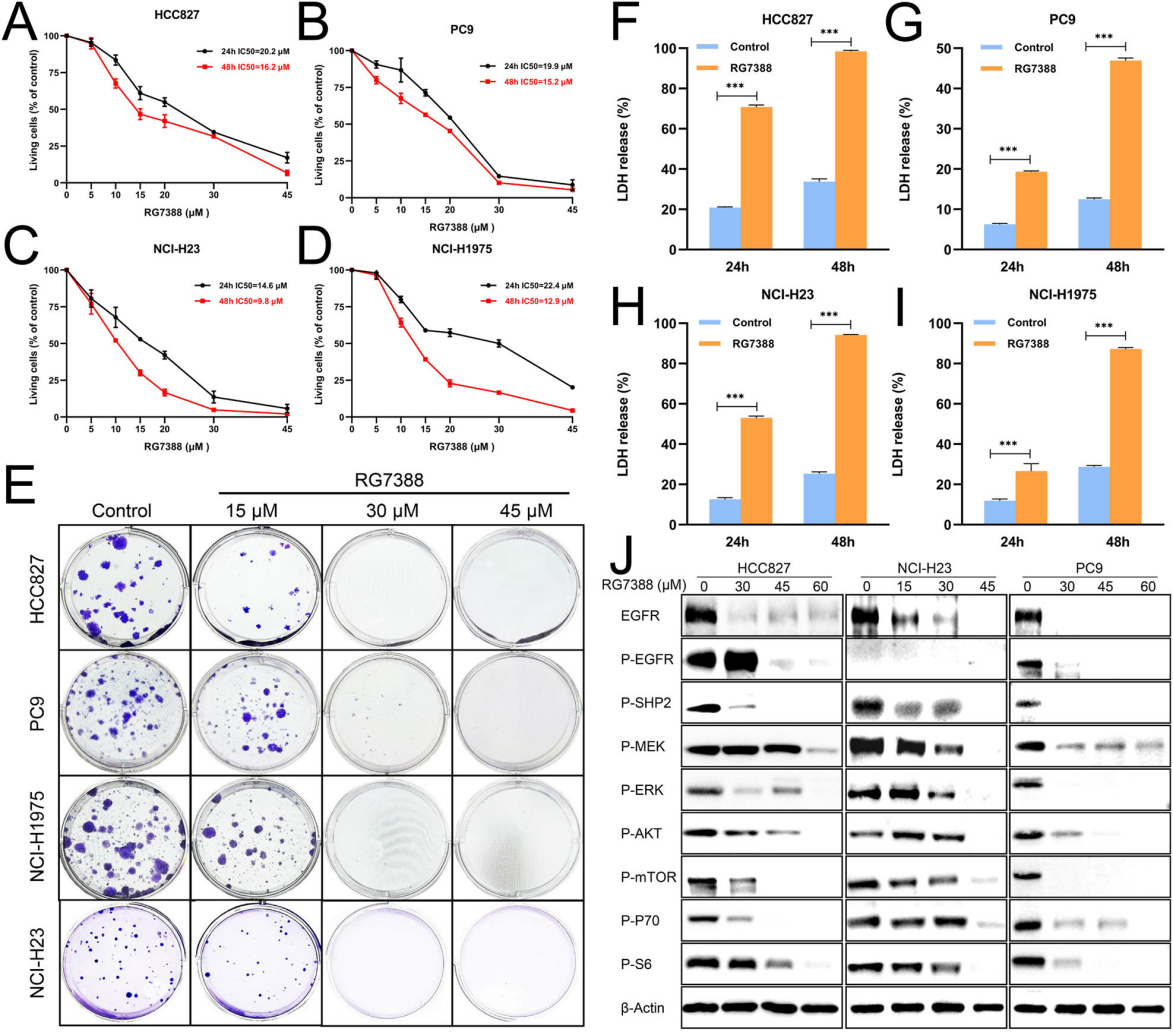
The cytotoxicity effect of RG7388 in *TP53*^mutant^ NSCLC. **A**–**D** IC50 assay for evaluating the sensitivity of a panel of *TP53*^mutant^ NSCLC cell lines to RG7388. HCC-827 (**A**), PC9 (**B**), NCI-H1975 (**C**) and NCI-H23 (**D**) cells were treated with RG7388 at the indicated concentrations for 24 or 48 hours. The percentage of cell growth was shown relative to DMSO control. Data are shown as mean ± SD. **E** *TP53*^mutant^ NSCLC cell lines (HCC-827, PC9, NCI-H1975 and NCI-H23) in 6-well plates were exposed to DMSO, 15, 30 or 45 μM RG7388 for 24 hours. After 2-3 weeks, the cells were fixed and stained. Representative staining images are shown. **F**–**I** Quantification of tumor cell death, measured by LDH release in RG7388 (60 μM) treated HCC827 (**F**), PC9 (**G**), H23 (**H**), and H1975 (**I**), was presented for the indicated time. All results are shown as the mean ± SEM (*n* = 5). Student’s *t-test* was used to analyze the data; ***p < 0.001. **J** Immunoblot analysis of the indicated EGFR signaling pathway proteins in HCC827 (*EGFR*^19del^), NCI-H23 (*EGFR*^WT^) and PC9 (*EGFR*^19del^) cells upon treatment with increasing concentrations of RG7388 for 24 hours.

In this study, we investigated the preclinical potential of RG7388 as an anti-*TP53*^mutant^ NSCLC agent. To assess its efficacy, we first performed CCK8 assays using four *TP53*^mutant^ cell lines with varying EGFR status (HCC827 (*TP53*^v218del^, *EGFR*^Ex19del^), PC9 (*TP53*^R248Q^, *EGFR*^Ex19del^), H23 (*TP53*^M246I^, *EGFR*^WT^) and H1975 (*TP53*^R273H^, *EGFR*^L858R/T790M^)). Our results demonstrated that RG7388 significantly reduced the cell viability of all four cell lines in a dose- and time-dependent manner, with detailed IC50 values at different time points presented in Fig. 1A-D. To further evaluate the anti-proliferative effects of RG7388, we treated these cell lines with increasing concentrations of RG7388 (0, 15, 30, and 45 μМ) for 24 hours and subjected them to a colony formation assay. RG7388 treatment induced a dose-dependent suppression of colony formation, highlighting its potent inhibitory effect on *TP53*^mutant^ NSCLC cell proliferation (Fig. 1E). Beyond proliferation inhibition, RG7388 triggered cell death in *TP53*^mutant^ NSCLC models. This was corroborated by lactate dehydrogenase (LDH) release assays, which showed significant cytotoxicity after 24 and 48 hours of treatment (Fig. 1F-I). Collectively, these findings suggested that RG7388 exerted dual anti-tumor effects by simultaneously inhibiting proliferation and promoting cell death in *TP53*^mutant^ NSCLC.

Epidermal growth factor receptor (EGFR) is a transmembrane glycoprotein belonging to the ERBB family of receptor tyrosine kinases (RTKs), which controls a variety of biological processes in cancer. Particularly in NSCLC, aberrant EGFR signaling is closely associated with malignant biological behaviors, including invasion and metastasis, tumor angiogenesis and chemoresistance [4, 34]. To investigate RG7388’s impact on this pathway, we performed western blot analysis in H23, HCC827, and PC9 cells. RG7388 treatment resulted in a dose-dependent reduction of EGFR protein expression and phosphorylation, accompanied by decreased activation of downstream effectors including SHP2, MEK1/2, ERK1/2, AKT, mTOR, p70(S6K1), and S6 (Fig. 1J). Notably, this suppression occurred in both EGFR-mutant (HCC827 and PC9) and EGFR wild-type (H23) cell lines, suggesting that RG7388’s ability to inhibit EGFR signaling was independent of EGFR status.

Collectively, these results demonstrated that RG7388 functioned as a multifaceted anti-tumor agent against various *TP53*^mutant^ NSCLC, exhibiting potent inhibition effects on proliferation and EGFR signaling pathways, as well as inductive effects on cell death.

### Molecular mechanisms of RG7388 induced cell death in *TP53*^mutant^ NSCLC

**Fig. 2:**
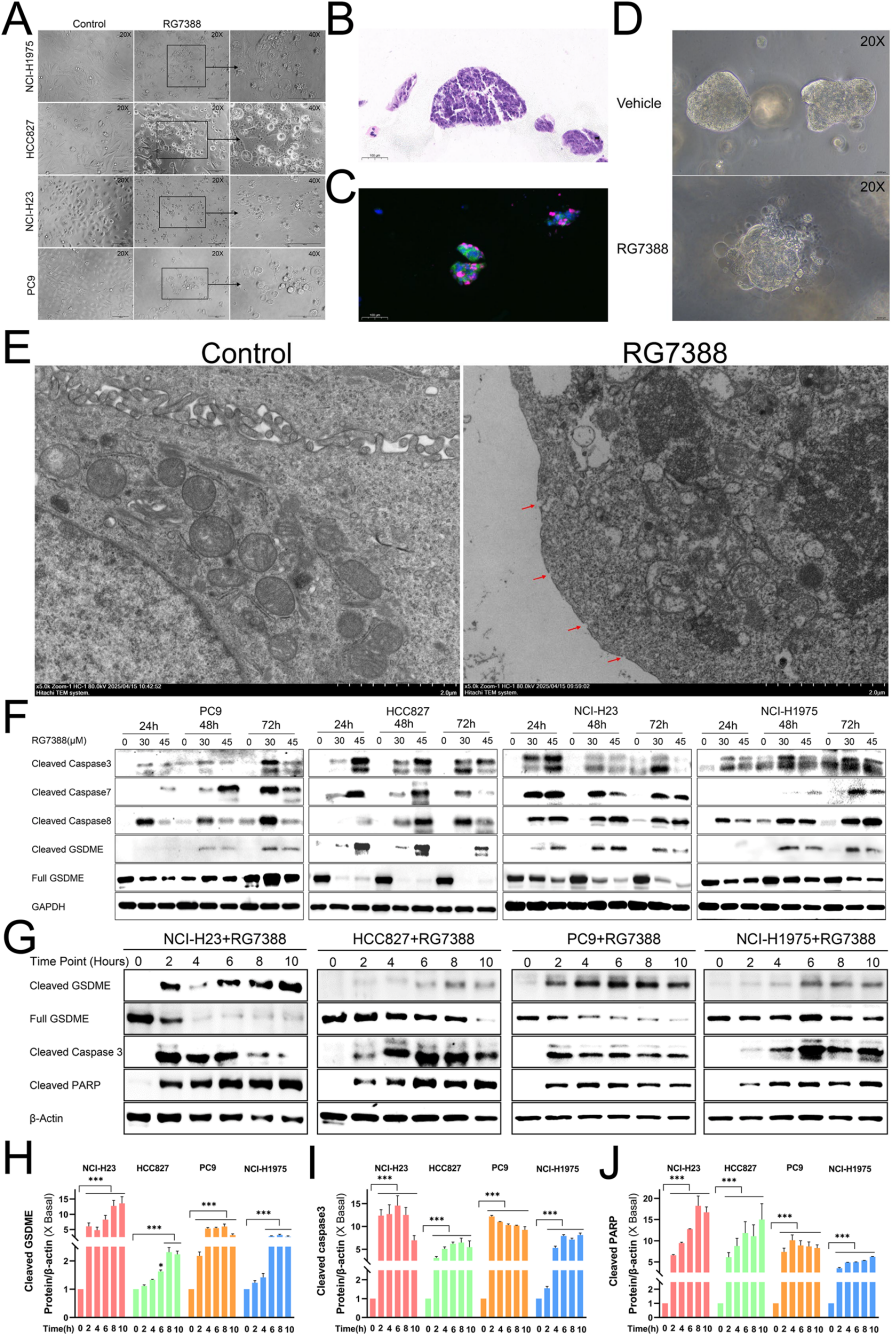
RG7388 triggered extrinsic apoptosis and pyroptosis pathways in *TP53*^mutant^ NSCLC cells. **A** Representative phase-contrast images of pyroptotic cell death induced by 60 μM RG7388 in *TP53*^mutant^ NSCLC cell lines as indicated for 6 or 24 h. **B**, **C** Representative H&E (**B**) and Immunofluorescence (IF) (**C**) staining of TTF-1 (green) and p63 (red) for *TP53*^mutant^ NSCLC organoids. Nuclei were stained with DAPI (blue). Scale bar, 100 μM (bottom panel). **D** Bright-field images of NSCLC organoids following treatment with 60 μM RG7388 or DMSO for 6 h. **E** Transmission electron microscopy observation of microstructural changes in PC9 cells treated by RG7388 (60 μM) for 6 hours. The red arrow indicated the pores on the plasma membrane. **F** Immunoblot analysis of protein abundance reflecting extrinsic apoptosis (cleaved caspase-3, cleaved caspase-7 and cleaved caspase-8) and pyroptosis (GSDME and cleaved GSDME) in RG7388 treated *TP53*^mutant^ NSCLC cell lysates collected at distinct concentrations. **G**–**J** Immunoblot analysis of protein abundance reflecting apoptosis (cleaved caspase-3 and cleaved PARP) and pyroptosis (GSDME and cleaved GSDME) in RG7388 (60 μM) treated *TP53*^mutant^ NSCLC cell lysates collected at distinct time points. The protein expression levels of cleaved GSDME (**H**), cleaved caspase-3 (**I**), and cleaved PARP (**J**) were semi-quantitatively assessed using ImageJ software. Quantitative data are presented as mean ± SD. ***p < 0.001.

Previous studies have established that RG7388 induces apoptosis in *TP53*^WT^ cancer (e.g., lung cancer and neuroblastoma) [12, 35, 36]. However, our observations revealed a distinctive feature in *TP53*^mutant^ NSCLC cells treated with RG7388. We noted that RG7388 clearly induced the characteristic morphological features of pyroptosis, as evidenced by cell swelling and the formation of membrane surface blebs, accompanied by a lack of organelles, which were not typical apoptotic features (Fig. 2A). To further determine the effect of RG7388 in preclinical models, we established patient-derived NSCLC organoids with *TP53* mutation for further in vitro analyses (Fig.2B and C). Interestingly, RG7388 induced one phenotype of cell death in the *TP53*^mutant^ NSCLC organoids similar to pyroptotic morphology in 2D-cultured *TP53*^mutant^ NSCLC cells (Fig. 2D). Ultrastructural analysis by transmission electron microscopy (TEM) further demonstrated that RG7388 treatment induced characteristic pyroptosis-associated morphology, including mitochondrial swelling with cristae disintegration and plasma membrane pore formation, accompanied by cytoplasmic swelling (Fig. 2E).

Based on the observed cellular swelling, membrane blebbing, and LDH release (Figs. 1F-I and 2A), combined with distinct ultrastructural hallmarks of apoptosis (mitochondrial cristae disorganization) and pyroptosis (gasdermin-mediated plasma membrane pores) (Fig. 2E), we hypothesized that both apoptosis and pyroptosis were involved in RG7388-induced cell death modalities. To validate this hypothesis, we examined apoptotic markers such as caspase-8, caspase-7 and caspase-3 and pyroptotic marker GSDME under 30 or 45 μM RG7388 treatment for extended durations (24 - 72 h). Concomitant cleavage of GSDME, caspase-8, caspase-7 and caspase-3 was observed in RG7388-treated PC9, HCC827 and H23 cells (Fig. 2F). Similarly, acute RG7388 treatment at a higher concentration of 60 μM could also induce the concurrent cleavage of GSDME, caspase-3 and PARP at the time points (2, 4, 6, 8, 10 h) (Fig. 2G-J). In the extrinsic apoptosis pathway, caspase-8 cleaves and thereby activates caspase-3 and caspase-7 to execute the apoptotic procedure [7]. These results verified that the extrinsic apoptosis and GSDME-mediated pyroptosis pathways were triggered in RG7388-treated *TP53*^mutant^ NSCLC cells.

### NOXA was essential for caspase-3-dependent apoptosis in RG7388-treated *TP53*^mutant^ NSCLC

**Fig. 3:**
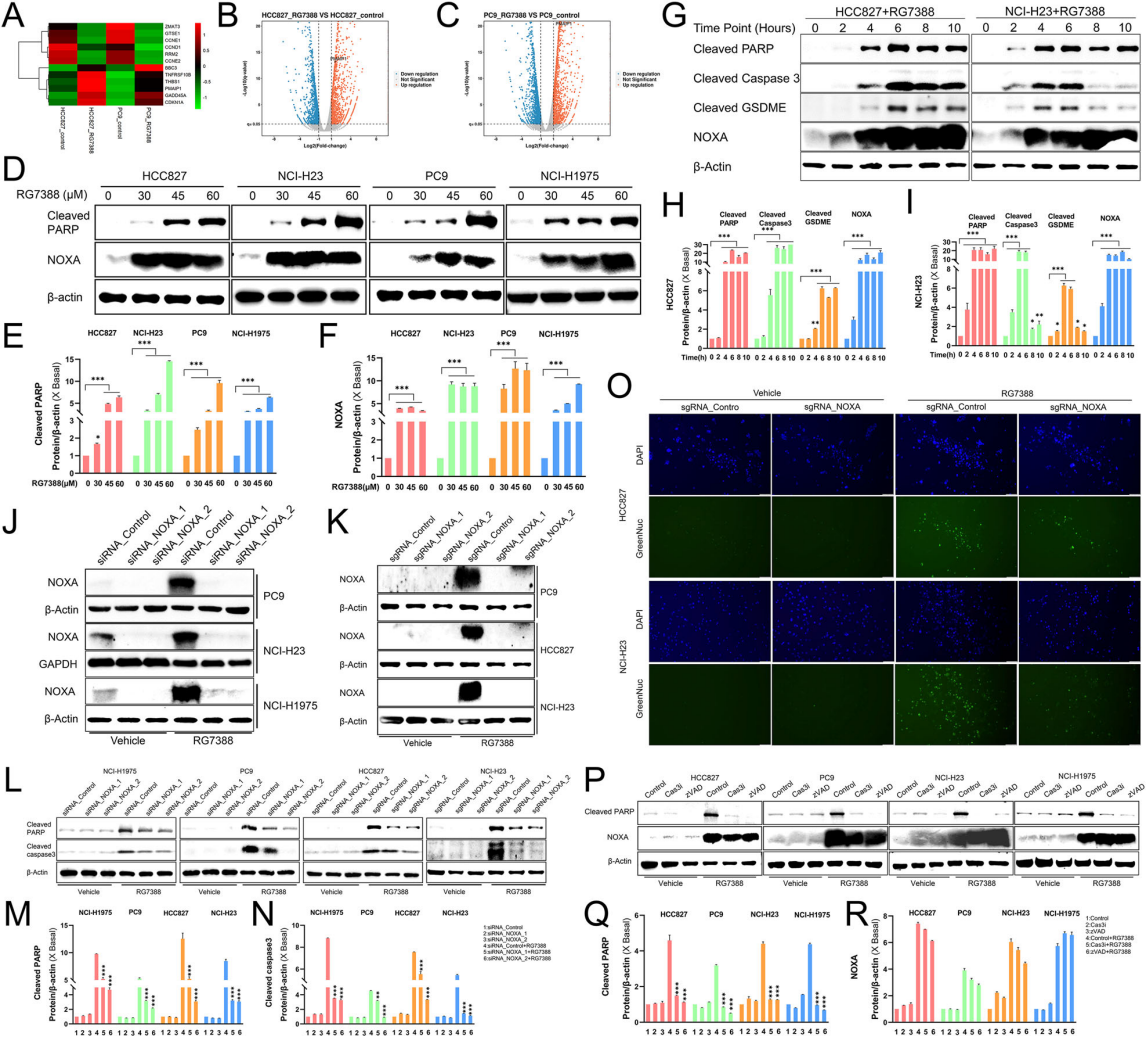
NOXA was essential for RG7388-induced apoptosis in *TP53*^mutant^ NSCLC. **A** Gene set enrichment analysis (GSEA) showed that the p53 signaling pathway was altered in RG7388 treated cells compared with control cells in HCC827 and PC9 cells. **B**, **C** The volcano plot showed the differentially expressed genes induced by RG7388 in HCC827 cells (**B**) or PC9 Cells (**C**). Blue dots indicated downregulated genes; orange dots indicated upregulated genes. **D**–**F** Immunoblot analysis of NOXA and cleaved PARP protein expression in HCC827, NCI-H23, PC9 and NCI-H1975 NSCLC cells treated with indicated concentrations of RG7388. The protein expression levels of cleaved PARP (**E**) and NOXA (**F**) were semi-quantitatively assessed using ImageJ software. Quantitative data are presented as mean ± SD. *p < 0.05, ***p < 0.001. **G**–**I** Immunoblot analysis of NOXA, cleaved PARP, cleaved caspase-3 protein, and Cleaved GSDME expression in HCC827 and NCI-H23 NSCLC cells treated with 60 μM RG7388 at indicated time course. The protein expression levels of cleaved PARP, cleaved caspase-3, cleaved GSDME and NOXA in HCC827 (**H**) or H23 (**I**) were semi-quantitatively assessed using ImageJ software. Quantitative data are presented as mean ± SD. *p < 0.05, **p < 0.01, ***p < 0.001. **J** Immunoblot analysis of the effect of NOXA(*PMAPI1*) knockdown in PC9, H23 and H1975 cells by using the RNA interference (RNAi) technique. **K** Immunoblot analysis of the effect of NOXA(*PMAPI1*) knockout in PC9, HCC827 and H23 cells by using the Crispr-Cas9 gene editing technique. **L**–**N** *TP53*^mutant^ NSCLC cells transfected with siRNA-mediated knockdown of NOXA (siRNA-NOXA), CRISPR / Cas9 mediated knock out of NOXA (sgRNA-NOXA) or control (siRNA-Control or sgRNA-Control) were treated by 60 μM RG7388 for 6 h. Immunoblot analysis of cleaved PARP and cleaved caspase-3 protein. β-Actin served as a loading control. The protein expression levels of cleaved PARP (**M**) and cleaved caspase-3 (**N**) were semi-quantitatively assessed using ImageJ software. Quantitative data are presented as mean ± SD. ***p < 0.001. **O** Representative immunofluorescence (IF) images of caspase-3 activity examined by the GreenNuc kit (Green) and cell nucleus stained by DAPI (blue) in RG7388 (60 μM) treated HCC827 or H23 with NOXA KO for 6 h. **P**–**R** Immunoblot analysis of NOXA and cleaved PARP protein expression in *TP53*^mutant^ NSCLC cells induced by RG7388 (60 μM) treated with or without total caspase inhibitor Z-VAD-FMK (zVAD, 50 μM) or caspase-3 inhibitor Z-DEVD-FMK (Cas3i, 25 μM) as indicated for 6 h. The protein expression levels of cleaved PARP (**Q**) and cleaved NOXA (**R**) were semi-quantitatively assessed using ImageJ software. Quantitative data are presented as mean ± SD. ***p < 0.001.

To explore the potential mechanisms that RG7388 induced cancer cell death in *TP53*^mutant^ NSCLC, we conducted an RNA-seq for control and RG7388-treated HCC827 and PC9 cells and found 685 differentially expressed genes (DEGs), including 311 upregulated genes and 374 downregulated genes (q < 0.05) (Supplementary Fig. 2A-C). Kyoto Encyclopedia of Genes and Genomes (KEGG) enrichment analysis of these DEGs revealed the p53 signaling pathway as one of the most affected cellular processes in RG7388-treated *TP53*^mutant^ NSCLC (Supplementary Fig. 2D and E). Among 685 DEGs, 12 genes were identified as the most significantly altered genes involved in the p53 signaling pathway (Fig. 3A). We focused our attention on the *PMAIP1* gene encoding pro-apoptotic NOXA protein because it was among the most notably upregulated genes in our RNA-seq results (Fig. 3B and C).

Consistent with the RNA-seq results, RG7388 treatment increased NOXA expression in a dose-dependent manner in HCC827, H23, PC9 and H1975 cells (Fig. 3D-F), and in a time-dependent manner in HCC827 and H23 cells (Fig. 3G-I). Of note, the potent time-dependent and dose-dependent upregulation of NOXA by RG7388 corresponded with induction of apoptosis (PARP and caspase-3 cleavage) and pyroptosis (GSDME cleavage) (Fig. 3D-I).

To further understand the role of NOXA in RG7388-induced cancer cell death, we knocked down NOXA in three *TP53*^mutant^ NSCLC cell lines by using two distinct siRNAs (Fig. 3J). In addition, we used CRISPR/Cas9 gene editing to create stable knockout (KO) of *PMAIP1* (NOXA) in three *TP53*^mutant^ NSCLC cell lines including H23, HCC827 and PC9 (Fig. 3K). Western blot results showed that NOXA knockdown (KD) or KO could significantly reverse the increased levels of cleaved PARP and cleaved caspase-3 upon the treatment of RG7388 in *TP53*^mutant^ NSCLC cells (Fig. 3L-N). Consistently, caspase-3 activity detected by the GreenNuc kit showed decreased green fluorescence signals in NOXA KO H23 or HCC827 cells under RG7388 treatment (Fig. 3O). In reverse, caspase-3 activity suppressed by pan-caspase inhibitor Z-VAD-FMK or caspase-3 inhibitor Z-DEVD-FMK did not affect the NOXA expression induced by RG7388 (Fig. 3P-R). The coordinated reduction of caspase-3 activation and PARP cleavage in NOXA-deficient cells suggested functional dependency on NOXA (Fig. 3L-R).

### RG7388 triggered GSDME-mediated pyroptosis through the NOXA/caspase-3 axis in *TP53*^mutant^ NSCLC

**Fig. 4:**
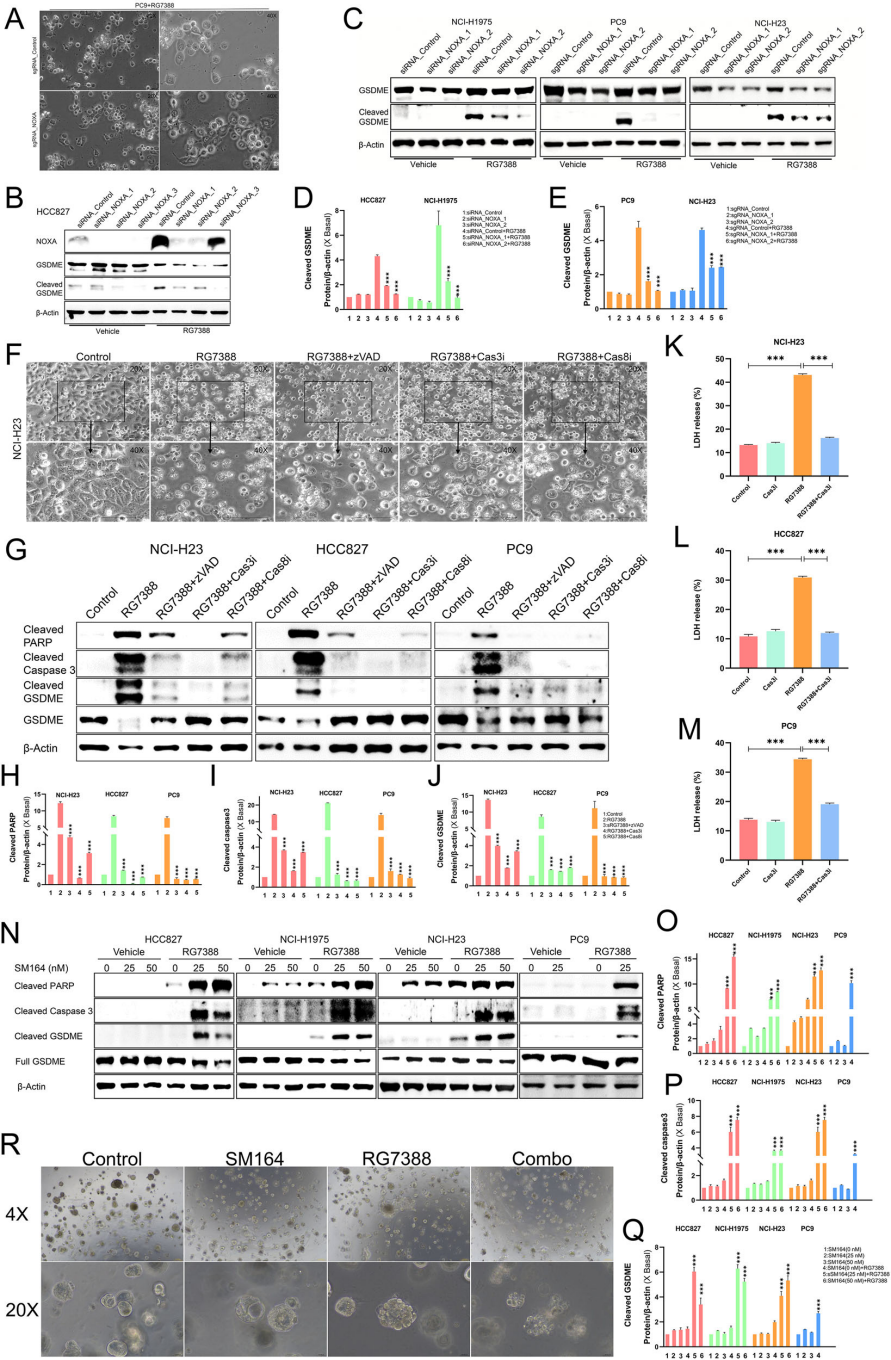
NOXA/caspase-3 axis regulated RG7388 induced pyroptosis in *TP53*^mutant^ NSCLC. **A** Representative phase-contrast images of pyroptotic cell death induced by RG7388 in PC9 control cells and NOXA knock-out cells. **B**–**E** *TP53*^mutant^ NSCLC cells transfected with siRNA-mediated knockdown of NOXA (siRNA-NOXA), CRISPR / Cas9 mediated knock out of NOXA (sgRNA-NOXA) or control (siRNA-Control or sgRNA-Control) were treated by 60 μM RG7388 for 6 hours. Immunoblot analysis of GSDME and cleaved GSDME protein. β-Actin served as a loading control. The protein expression levels of cleaved GSDME were semi-quantitatively assessed using ImageJ software. Quantitative data are presented as mean ± SD. ***p < 0.001. **F** Representative phase-contrast images of pyroptotic cell death induced by RG7388 in H23 cells pre-treated with or without pan-caspase inhibitor Z-VAD-FMK (zVAD, 50 μM), caspase-8 inhibitor Z-IETD-FMK (Cas8i, 50 μM) or caspase-3 inhibitor Z-DEVD-FMK (Cas3i, 25 μM) as indicated for 6 hours. **G**–**J** Immunoblot analysis of cleaved PARP, cleaved caspase-3, GSDME and cleaved GSDME in the indicated cells. The cells were pretreated with 50 μM zVAD, 25 μM Cas3i or 50 μM Cas8i for 1 hour and then treated with 60 μM RG7388 for 6 h. The protein expression levels of cleaved PARP (**H**), cleaved caspase-3 (**I**), and cleaved GSDME (**J**) were semi-quantitatively assessed using ImageJ software. Quantitative data are presented as mean ± SD. ***p < 0.001. **K**–**M** Cytotoxicity was detected using the LDH assay in H23 (**K**), HCC827 (**L**) or PC9 (**M**) cells induced by RG7388 (60 μM) in the presence or absence of Z-DEVD-FMK (Cas3i, 25 μM). All results are shown as the mean ± SEM (*n* = 5). ***p < 0.001 using Student’s *t-test*. **N**–**Q** Immunoblot analysis of cleaved PARP, cleaved caspase-3, GSDME, and cleaved GSDME in the indicated cells treated with RG7388 (15 μM), SM164 (25 or 50 nM) or the combination of RG7388 and SM164 for 6 or 24 h. The protein expression levels of cleaved PARP (**O**), cleaved caspase-3 (**P**), and cleaved GSDME (**Q**) were semi-quantitatively assessed using ImageJ software. Quantitative data are presented as mean ± SD. ***p < 0.001. **R** Bright-field microscopic images of *TP53*^mutant^ NSCLC organoids treated with RG7388 (15 μM), SM164 (25 nM), or the combination of RG7388 and SM164 for 24 h.

Since NOXA was essential in RG7388-induced apoptosis in *TP53*^mutant^ NSCLC cells (Fig. 3), we then investigated whether NOXA was involved in RG7388-induced pyroptosis. Morphological changes in cells, an indicator of pyroptosis, were first observed by microscopic examination. As expected, NOXA KO reversed the phenotype of the RG7388-treated PC9 cells from pyroptosis to a normal state (Fig. 4A). Mechanistically, western blot analysis demonstrated that genetic disruption of NOXA (via either RNAi-mediated knockdown or CRISPR/Cas9 knockout) significantly attenuated RG7388-induced proteolytic cleavage of GSDME into its pore-forming N-terminal fragment in *TP53*^mutant^ NSCLC cells (Fig. 4B-E). This molecular suppression of GSDME activation directly correlated with the observed inhibition of pyroptosis.

Similar to apoptosis, pyroptosis is regulated by caspases [21]. caspase-3, the executioner caspase activated by cleavage after Caspase-8 activation in apoptosis, was shown to cleave and activate GSDME resulting in pyroptosis [16]. To further clarify the role of the NOXA/caspase-3 axis in RG7388-induced pyroptosis, we conducted co-incubation of RG7388 with Z-VAD-FMK, an apoptotic inhibitor, which reversed the phenotype of the RG7388-treated H23 cells from pyroptosis to normal state and prevented RG7388-induced cleavage of caspase-3, PARP and GSDME in H23, HCC827 and PC9 cells (Fig. 4F-J). Similarly, Z-DEVD-FMK (a caspase-3 inhibitor) and Z-IETD-FMK (a caspase-8 inhibitor) were also effective in reversing the pyroptotic morphology and suppressing RG7388-induced cleavage of apoptotic and pyroptotic markers (Fig. 4F-J). Consistently, LDH release in response to RG7388 treatment was substantially attenuated by pretreatment with Z-DEVD-FMK in H23, HCC827 and PC9 cells (Fig. 4K-M). These results indicated that inhibition of extrinsic apoptosis by inhibitors of the caspase-8/caspase-3 axis prevented *TP53*^mutant^ NSCLC cells from proptosis induced by RG7388.

X-linked inhibitor of apoptosis protein (XIAP) as one E3 ubiquitin ligase can directly bind and potently suppress the activity of caspase-3 which is a critical effector of apoptosis [37, 38]. SM164, developed as one small-molecule second mitochondria-derived activator of caspases (Smac) mimetics, can effectively antagonize XIAP to remove inhibition of caspase-3 [39]. To further investigate the role of caspase-3 in regulating RG7388-induced apoptosis and pyroptosis, we selected SM164 to activate caspase-3 cleavage. Treatment with SM164 and RG7388 strongly induced cleavage of caspase-3 and its downstream target PARP (Fig. 4N-Q), which are critical hallmarks of apoptosis. Simultaneously, we synergistically used SM164 and RG7388 to confirm that direct activation of caspase-3 enhanced the efficiency of GSDME cleavage, releasing an N-terminal fragment that oligomerizes and forms pores in membranes (Fig. 4N-Q). Furthermore, we tested the efficacy of the combination therapy in the *TP53*^mutant^ NSCLC organoid model. Consistent with the trend for cleaved PARP, cleaved caspase-3 and cleaved GSDME, optical microscopy revealed significant rupture and disintegration in the *TP53*^mutant^ NSCLC organoid after treatment of the combination (Fig. 4R). Taken together, these results showed that SM164 synergistically enhanced RG7388-induced apoptosis and pyroptosis via activating caspase-3 cleavage.

Eukaryotic cells exposed to different types of environmental stress such as oxidative stress rapidly form stress granules (SGs) structures [40]. SGs are cytoplasmic condensates composed of RNA and RNA-binding proteins including GTPase-activating protein (SH3 domain)-binding proteins 1 and 2 (G3BP1 and G3BP2) [41]. As core components of (SGs), G3BP1 or G3BP2 functions to protect RNAs from harmful conditions [42]. When facing anti-tumor therapies, cancer cells also assemble SGs which is involved in cancer cell death [43]. Interestingly, G3BP1 and G3BP2 exhibit differential activity and functions in cancer [43]. To understand the impact of G3BP1 or G3BP2 on the RG7388-induced NOXA/Caspase-3 axis-mediated cancer cell death, we utilized siRNA technology to knock down G3BP1 and G3BP2, respectively. Successful siRNA-mediated silencing of G3BP1 or G3BP2 protein expression was verified in HCC827 cells using Western blot analysis (Supplementary Fig. 2A). G3BP2 KD, but not G3BP1 KD, significantly alleviated RG7388-induced concurrent cleavage of GSMDE, caspase-3 and PARP in HCC827 cells (Supplementary Fig. 2B). As expected, G3BP2 KD also suppressed RG7388-induced NOXA upregulation whereas G3BP1 KD did not affect NOXA expression (Supplementary Fig. 2C). It indicated that NOXA was capable to significantly reduce caspase-3 activity and subsequent cleavage of PARP and GSDME in G3BP2 KD HCC827 cells upon RG7388 treatment. To further validate the key role of the NOXA/caspase-3 axis in G3BP2-regulated pyroptosis, we subsequently created stable G3BP1 and G3BP2 KO cell lines in H23 using CRISPR-Cas9 technology (Supplementary Fig. 2D) and exposed them to RG7388. Our findings also indicated that it was G3BP2 KO, not G3BP1 KO, that decreased the sensitivity of H23 cells to pyroptosis via suppressing the NOXA/caspase-3 axis (Supplementary Fig. 2E). Morphologically, the pyroptotic features characterized by cellular swelling and membrane blebbing in RG7388 treated H1975 cells were also mitigated following G3BP2 KO (Supplementary Fig. 2F). These experiments further confirmed that GSDME-mediated pyroptosis was the secondary mechanism of cell death induced by RG7388 which triggered the primary apoptosis via the NOXA/caspase-3 axis.

Collectively, our data suggested that the NOXA/caspase-3 axis was the key determinant of RG7388-induced cancer cell death in *TP53*^mutant^ NSCLC.

### Mitochondria-derived ROS activated p38 signaling to upregulate NOXA in *TP53*^mutant^ NSCLC

Although RG7388 induced a concomitant decrease of mutant p53 expression with an increase of cleaved PARP and cleaved Caspase-3 in a dose-dependent manner (Supplementary Fig. 3A and B), *TP53* KD did not cause significant change in the expression of NOXA in *TP53*^mutant^ NSCLC cells (Supplementary Fig. 3C). These data suggested that RG7388 induced NOXA upregulation and subsequent caspase-3 activation in a mutant p53-independent manner.

**Fig. 5:**
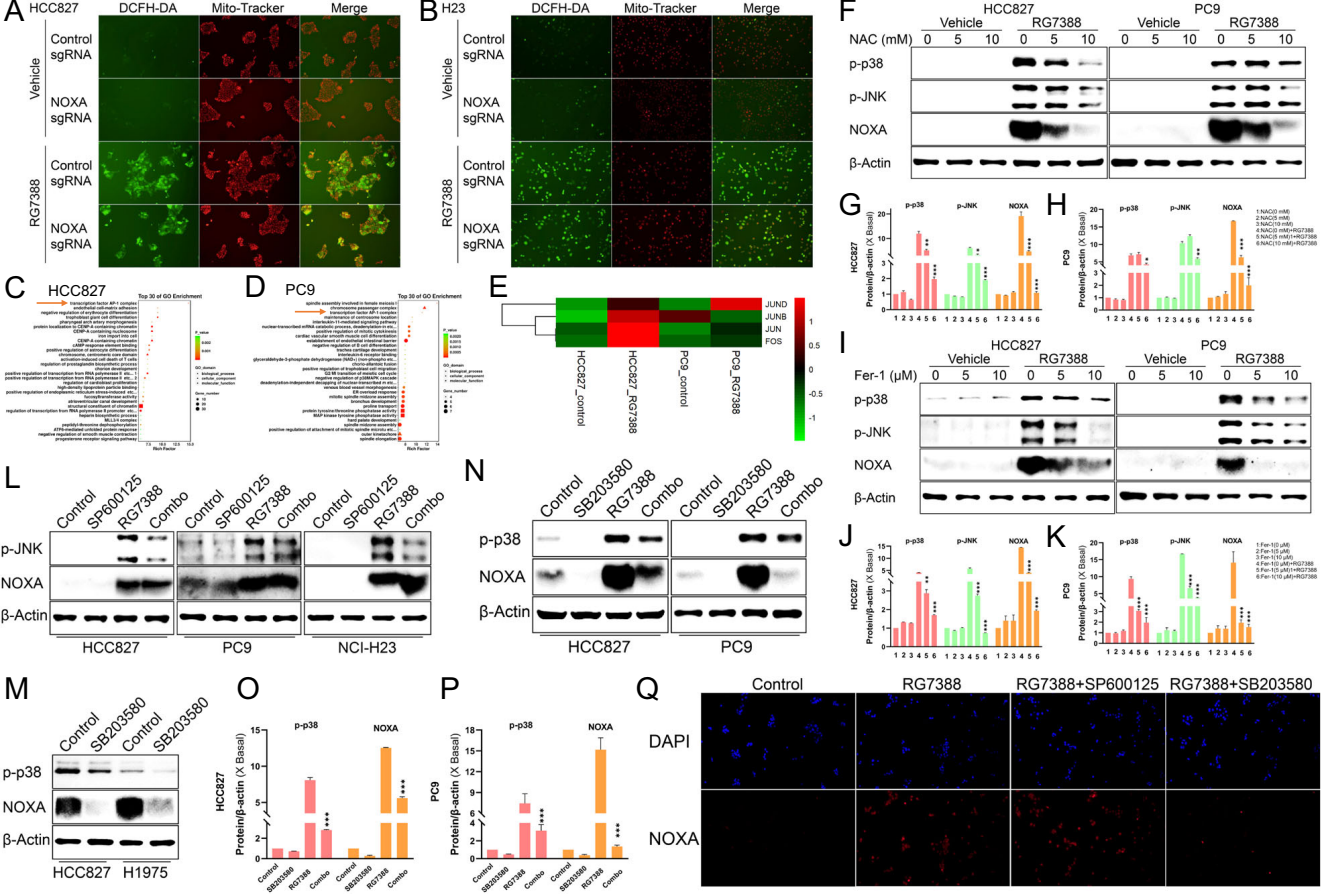
Mitochondrial ROS induced by RG7388 upregulated NOXA via p38 pathway. **A**, **B** Detection of ROS by immunofluorescence of HCC827 (**A**) or H23 (**B**) cells stably transfected with non-targeting sgRNA or sgRNA-NOXA. ROS staining (green) and mitochondrial staining (red) are shown. **C**–**E** Gene ontology (GO) enrichment analysis using differentially expressed genes showed strong clustering of transcription factor AP-1 complex genes (*JUND, JUNB, JUN* and *FOS*) which were altered in RG7388 treated cells compared with control cells in HCC827 and PC9 cells. **F**–**K** Immunoblot analysis of p-p38, p-JNK, NOXA and β-actin expression in HCC827 or PC9 cells pre-treated with total ROS inhibitor NAC (**F**) or lipid ROS inhibitor Fer-1 (**I**) at the indicated concentration for 1 hour and then treated with 60 μM RG7388 for 6 hours. The protein expression levels of p-p38, p-JNK, and NOXA in HCC827 (**G**, **J**) or PC9 (**H**, **K**) were semi-quantitatively assessed using ImageJ software. Quantitative data are presented as mean ± SD. *p < 0.05, **p < 0.01, ***p < 0.001. **L** Immunoblot analysis of p-JNK, NOXA and β-actin expression in HCC827, PC9 or H23 cells pre-treated with 40 μM JNK inhibitor SP600125 for 1 hour and then treated with 60 μM RG7388 for 6 hours. **M** Immunoblot analysis of p-p38, NOXA and β-actin expression in HCC827 or H1975 cells treated with p38 inhibitor SB203580 (40 μM) for 6 h. **N**–**P** Immunoblot analysis of p-p38, NOXA and β-actin expression in HCC827 or PC9 cells pre-treated with 40 μM SB203580 for 1 hour and then treated with 60 μM RG7388 for 6 h. The protein expression levels of p-p38 and NOXA in HCC827 (**O**) or PC9 (**P**) were semi-quantitatively assessed using ImageJ software. Quantitative data are presented as mean ± SD. ***p < 0.001. **Q** Representative IF images of NOXA intensity (Red) and nucleus stained with DAPI (blue) in HCC827 cells pre-treated with 40 μM SP600125 or SB203580 for 1 hour and then treated with 60 μM RG7388 for 6 h.

Excessive ROS have been regarded as an executioner of cell death and determines cell death modalities including apoptosis and pyroptosis [23, 44–46]. To investigate ROS generation and derivation in RG7388-treated *TP53*^mutant^ NSCLC cells, we initially examined intracellular ROS levels using double staining with DCFH-DA and Mito-Tracker Red. Over-productive ROS, as indicated by stronger DCFH-DA signals, were observed in RG7388-treated HCC827 or H23 cells. Although it was reported that NOXA induced ROS accumulation [47], our current research showed that NOXA KO could not reverse RG7388-induced ROS overproduction which meant that ROS might be the upstream regulator of NOXA. In addition, the green fluorescence from DCFH-DA exhibited significant overlaps with the Mito-Tracker Red in the cytoplasmic region, indicating that the mitochondrion was the source of RG7388-induced ROS generation (Fig. 5A and B).

To further delve into the key signaling pathway in upregulating NOXA in *TP53*^mutant^ NSCLC, we employed global RNA-seq analysis to pinpoint the pathways influencing NOXA. GSEA analysis validated that Gene Ontology terms associated with the “transcription factor activator protein 1 (AP-1) complex” signature was strongly enriched in RG7388-treated cells compared to vehicle-treated cells in HCC827 and PC9 cells (Fig. 5C and D). The significantly altered AP-1 complex genes (*JUND, JUNB, JUN* and *FOS*) (Fig. 5E) are transcription factors (TFs) which are mainly regulated by mitogen-activated protein kinases (MAPKs) of p38 and JUN amino-terminal kinase (JNK) signaling pathways [48, 49]. Moreover, western blotting results in our study showed RG7388 induced a concomitant activation of JNK and p38 with upregulation of NOXA in HCC827 or PC9 cells (Fig. 5F-K). Accordingly, NOXA might be associated with the MAPK signaling pathways. Furthermore, a substantial number of studies have demonstrated that ROS is an important intracellular antitumorigenic signaling including MAPK pathways and then trigger oxidative stress-induced cancer cell death [45, 46, 49–51]. Therefore, we hypothesized that RG7388 might induce NOXA upregulation via ROS-activated JNK and/or p38 pathways.

To test this hypothesis, we first employed inhibition experiments by utilizing N-Acetyl-L-cysteine (NAC), a ROS scavenger. The co-treatment with NAC effectively inhibited phosphorylation of JNK and p38 and NOXA upregulation induced by RG7388 in a dose-dependent manner, respectively (Fig. 5F-H). Ferrostatin-1 (Fer-1) as a synthetic antioxidant can also alleviate ROS production [52]. As expected, Fer-1 exhibited a reverse effect similar to NAC (Fig. 5I-K). It indicated that phosphorylation of JNK or p38 further correlated with NOXA upregulation in RG7388-treated *TP53*^mutant^ NSCLC cell lines.

To determine whether activation of both MAPK pathways was mechanistically linked to the upregulation of NOXA, we used SP600125 or SB203580 to inhibit the JNK or p38 signaling pathway. Western blot analysis revealed that SP600125 and SB203580 suppressed the phosphorylation of JNK and p38 induced by RG7388, respectively (Fig. 5L-N). SP600125 treatment had no significant effect on the expression of NOXA induced by RG7388 (Fig. 5L). It suggested that the JNK pathway was not essential for NOXA induction by RG7388. However, after treatment with SB203580 for 6 h in HCC827 or H1975 cells, it was found that the protein level of endogenous NOXA could be significantly downregulated (Fig. 5M). Further, pharmacological interference with the p38 signaling could suppress RG7388-induced NOXA upregulation in HCC827 and PC9 cells (Fig. 5N-K). Immunofluorescence validation in HCC827 cells further demonstrated p38-specific regulation, where SB203580, but not SP600125, suppressed NOXA upregulation (Fig. 5Q). Collectively, these findings established that RG7388-induced ROS activated p38 signaling to drive NOXA expression in *TP53*^mutant^ NSCLC cells.

### Pharmacological inhibition of p38 signaling attenuated RG7388-induced cell death in *TP53*^mutant^ NSCLC

**Fig. 6:**
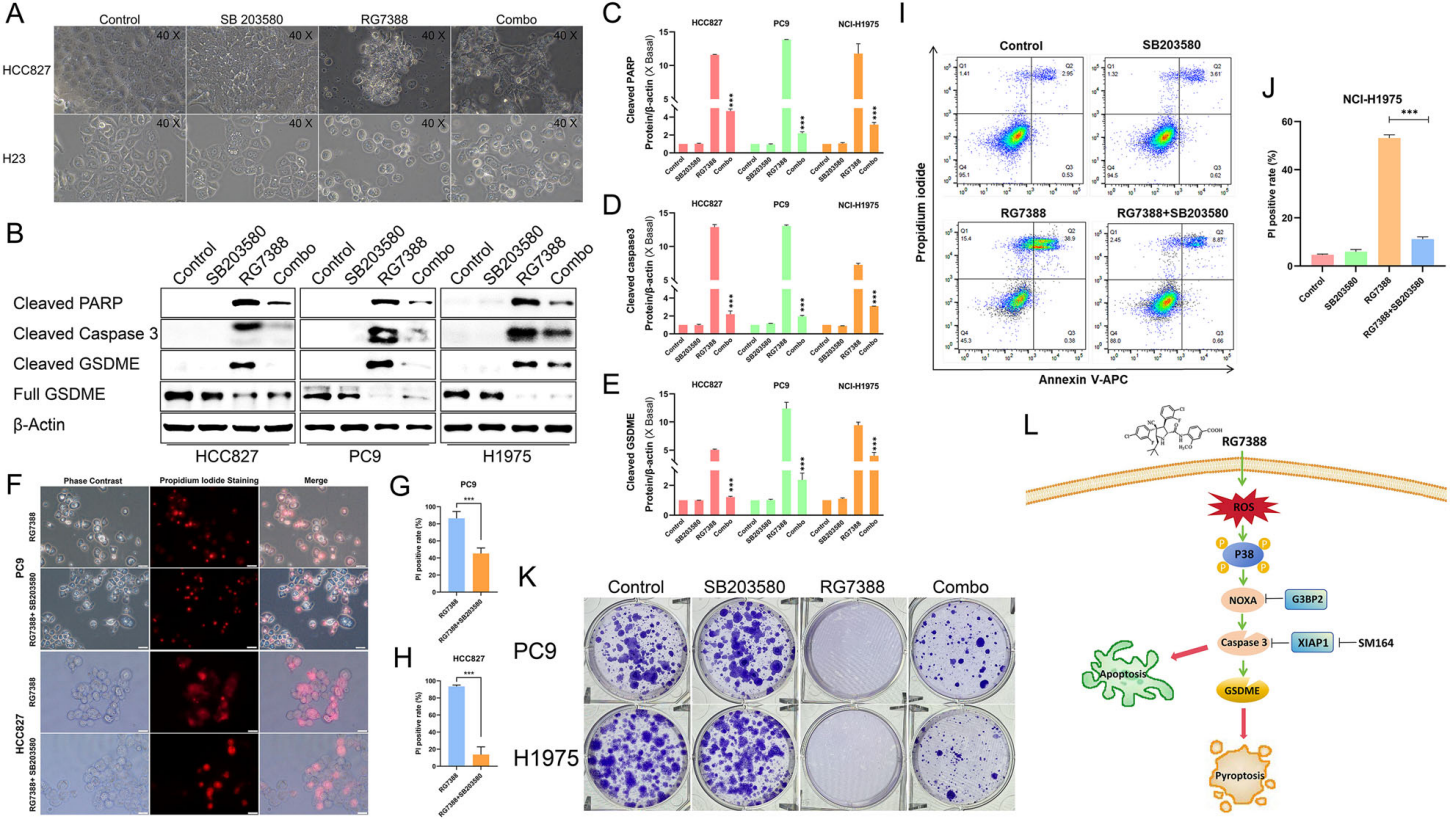
Pharmacological inhibition of the p38 pathway prevented *TP53*^mutant^ NSCLC cells from RG7388-induced cell death. **A** Representative phase-contrast images of pyroptotic cell death in HCC827 or H23 cells treated by RG7388 (60 μM), SB203580 (40 μM) or the combination of RG7388 and SB203580 for 6 hours. **B**–**E** Immunoblot analysis of cleaved PARP, cleaved caspase-3, GSDME, and cleaved GSDME in the indicated cells treated with RG7388 (60 μM), SB203580 (40 μM) or the combination of RG7388 and SB203580 for 6 hours. The protein expression levels of cleaved PARP (**C**), cleaved caspase-3 (**D**), and cleaved GSDME (**E**) were semi-quantitatively assessed using ImageJ software. Quantitative data are presented as mean ± SD. ***p < 0.001. **F**–**H** PC9 and HCC827 cells were treated with RG7388 or the combination of RG7388 and SB203580 for 6 hours. The dead cells were detected by Propidium Iodide (PI) staining. Representative images were shown (**F**). The percentage of PI-positive cells was counted (mean ± SE, *n* = 5 randomly selected microscope fields) (**G**, **H**). Student’s *t-test* was used to analyze the data; ***P < 0.001. **I**, **J** Annexin V/PI flow cytometry analysis of H1975 cells under RG7388, SB203580 or combined treatment for 6 h (**I**). The percentage of PI-positive cells was counted (mean ± SE, *n* = 3) (**J**). ***P < 0.001. **K** PC9 or H1975 cells in 6-well plates were exposed to DMSO, RG7388, SB203580 or the combination of RG7388 and SB203580 for 6 hours. After 3-4 weeks, the cells were fixed and stained. Representative staining images are shown. **L** Schematic illustration of ROS/p-p38/NOXA/caspase-3 axis in RG7388 induced *TP53*^mutant^ NSCLC cell death.

To determine whether cancer cell death caused by NOXA upregulation was due to activation of p38 signaling, we first treated the HCC827 and H23 with RG7388 in the presence of p38 MAPK inhibitor SB203580. The combination of RG7388 and SB203580 caused HCC827 and H23 cells to retain an almost normal morphology in comparison to RG7388 alone (Fig. 6A). Further analysis using a western blot assay revealed that inhibition of p38 signaling could significantly alleviate RG7388-induced concurrent cleavage of GSDME, caspase-3 and PARP in *TP53*^mutant^ NSCLC cell lines (Fig. 6B-E). Subsequently, reduced RG7388 cytotoxicity under p38 signaling inhibition was confirmed by using propidium iodide (PI) staining in PC9 and HCC827 cells (Fig. 6F-H). Moreover, the addition of SB203580 to RG7388 induced less cell death according to PI/Annexin-V staining using flow cytometry (FACS) analysis compared to either agent alone in H1975 cells (Fig. 6I and J). More strikingly, in a colony formation assay (Fig. 6K), SB203580 treatment alone had little effect but was able to effectively rescue the RG7388-induced severe reduction in colony number in PC9 and H1975 cells. Taken together, these findings demonstrated that ROS-driven p38 activation mediated RG7388-induced cell death via NOXA upregulation (Fig. 6L).

### Clinical relevance of p-p38 MAPK/NOXA axis in NSCLC

**Fig. 7:**
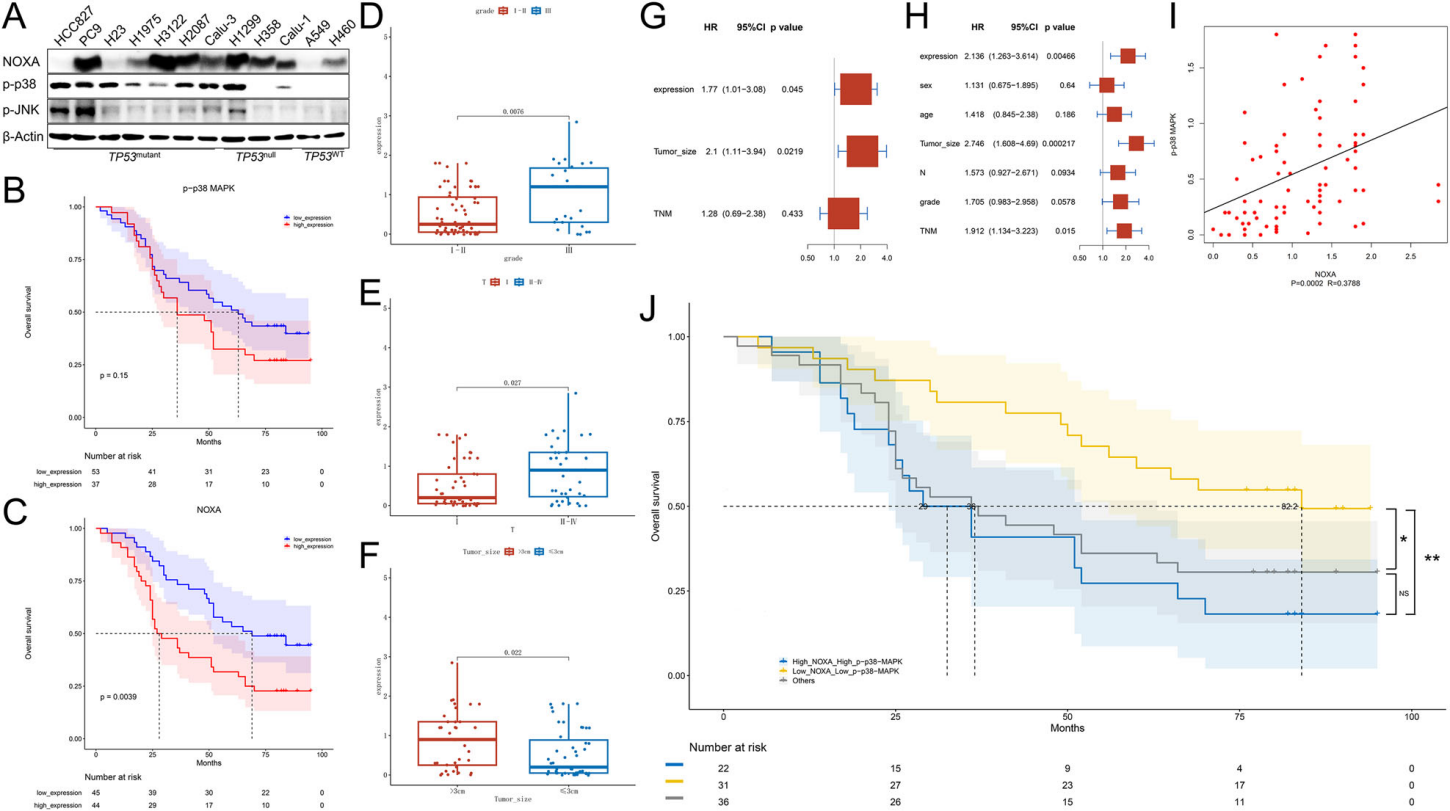
Survival analysis of p-p38/NOXA axis in NSCLC patients. **A** Immunoblot analysis was performed to evaluate the expression of NOXA, p-p38 and p-JNK in *TP53*^mutant^ (HCC827, PC9, H23, H1975, H3122, H2087 and Calu-3), *TP53*^null^ (H1299, H358 and Calu-1) and *TP53*^wt^ (A549 and H460) cell lines. **B**, **C** Overall survival curves in NSCLC patients with differential expression of p-p38 MAPK (**B**) or NOXA (**C**) were calculated by Kaplan-Meier analysis and compared with the Log-rank test. All results are shown as the mean ± SEM (*n* = 5). One- or two-way ANOVA was used to analyze the data; *p < 0.05, **p < 0.01, ***p < 0.001. **D**–**F** The associations between NOXA expression and the clinicopathological parameters of NSCLC patients including histological grade (**D**), T stage (**E**) and tumor size (**F**) were analyzed. **G**, **H** Univariate (**G**) and multivariate (**H**) analyses of the overall survival rate of NSCLC patients with the Cox proportional hazards model. **I** Pearson correlation coefficients of p-p38 and NOXA in tumor tissue chips from NSCLC patients and the *p* values are shown. **J** Overall survival curves in NSCLC patients with differential expression of combinations of p-p38 MAPK and NOXA were calculated by Kaplan–Meier analysis and compared with the Log-rank test. One- or two-way ANOVA was used to analyze the data; *p < 0.05, **p < 0.01.

We first evaluated the expression of p-p38, NOXA and p-JNK in *TP53*^mutant^, *TP53*^null^ and *TP53*^WT^ human NSCLC cell lines. Although we did not observe a positive correlation between p-p38 and NOXA levels across a panel of NSCLC cell lines, the expression levels of p-p38 or NOXA compared to that of p-JNK were higher in the majority of the panel of NSCLC cell lines (Fig. 7A). Subsequently, to explore the expression of NOXA and p-p38 in clinical NSCLC patients’ tissues, immunohistochemical (IHC) staining and scoring of p-p38 and NOXA was performed in NSCLC tissue microarray (TMA) chips containing 90 pairs of NSCLC and adjacent tissues (Supplementary Fig. 4A and C). Consistent with the results in Fig. 7A, the protein expression level of p-p38 or NOXA in NSCLC tumor tissues was found to be significantly higher than in adjacent tissues (Supplementary Fig. 4B and D).

We then analyzed the correlation between p-p38 or NOXA expression level and clinical pathological features of NSCLC patients. Although the clinical significance of p-p38 expression level was not statistically significant (*P* = 0.15) (Fig. 7B), Kaplan–Meier survival analysis showed that patients with high expression of NOXA (*n* = 44) had shorter overall survival (OS) than those with low expression (*n* = 45) (*P* = 0.0039) (Fig. 7C). Moreover, the correlation between high expression of NOXA and adverse clinical pathological features including histological grade (*P* = 0.0076), T stage (*P* = 0.027) and tumor size (*P* = 0.022) were confirmed by statistical analysis (Fig. 7D-F). In accordance, univariate and multivariate Cox regression analysis showed that NOXA expression, tumor size and TNM stage were independent prognostic factors for OS (Fig. 7G and H). These results determined NOXA to be an independent predictor of OS in NSCLC. Similar results were found in The Cancer Genome Atlas (TCGA) database of NSCLC. The expression of *PMAIP1* (NOXA) was significantly higher in the tumor tissues compared to normal tissues, as demonstrated by TCGA analysis (Supplementary Fig. 4E). Moreover, *PMAIP1* (NOXA) overexpression was associated with shorter overall survival in the TCGA databases (Supplementary Fig. 4F). Together, these results suggested that high NOXA expression was an independent indicator of poor prognosis for NSCLC patients.

Since the p-p38/NOXA axis was confirmed in our current study (Figs. 6 and 7), we then analyzed the relationship between p-p38 and NOXA in the protein expression level in human NSCLC tissues and found that p-p38 expression was positively correlated with NOXA expression in NSCLC (R = 0.3788, *P* = 0.0002) (Fig. 7I). Considering that NOXA could be an independent predictor of OS in NSCLC patients (Fig. 7C-H), we further sought to determine the clinical significance of the p-p38/NOXA axis. Based on the positive expression levels of p-p38 and NOXA, we divided the patient cohort into three groups, where NOXA^high^ and p-p38^high^ group (red) had high p-p38 and high NOXA, NOXA^low^ and p-p38^low^ (green) group had low p-p38 and low NOXA and others group (blue) had the remaining patients. A survival analysis showed that patients in the NOXA^low^ and p-p38^low^ group had the best prognoses (Fig. 7J). In conclusion, our results suggested that the p-p38/NOXA axis might be an effective indicator for predicting overall survival in NSCLC patients, which could improve the prognostic precision indicated by single NOXA expression. These findings also suggested that NSCLC cells might be already primed towards a proapoptotic state through higher basal expression of p-p38 and NOXA, which render them more sensitive to primary apoptosis and secondary pyroptosis upon RG7388 treatment.

### RG7388 induced the remodeling of the tumor environment in vitro

**Fig. 8:**
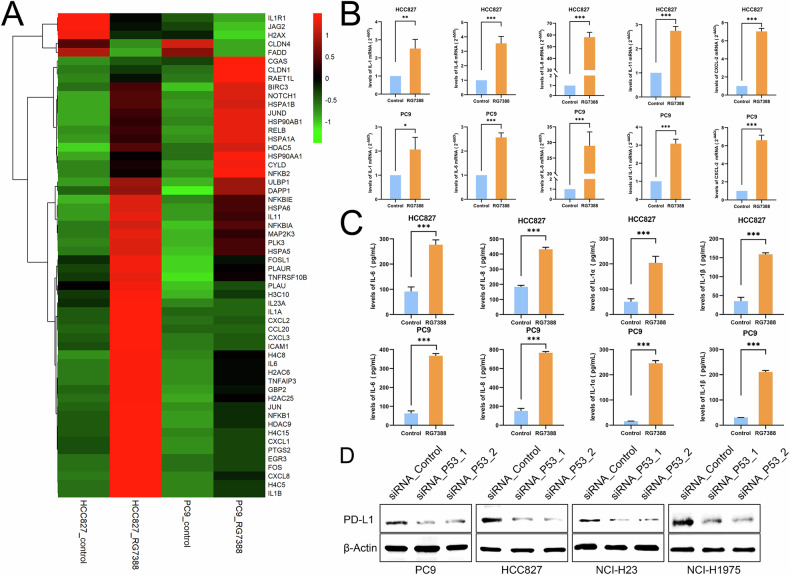
RG7388 remodeled TME in *TP53*^mutant^ NSCLC. **A** The heatmap displayed the expression ratios of differentially expressed immune system-related genes in RG7388-treated HCC827 or PC9 cells. **B** Real-time PCR analysis for the relative expression levels of the immune-related genes in HCC827 or PC9 cells treated with 60 μM RG7388 or DMSO for 6 h. Data were presented as mean ± SEM from three independent experiments. Student’s *t-test* was used to analyze the data; *p < 0.05, **p < 0.01, ***p < 0.001. **C** HCC827 or PC9 cells were treated with 60 μM RG7388 or DMSO for 6 h. Supernatants were collected and assayed for IL-6, IL-8, IL-1α and IL-1β levels by ELISA. Data were represented as means ± SEM from three independent experiments. Student’s *t-test* was used to analyze the data; ***p < 0.001. **D** Protein levels of PD-L1 in *TP53* knockdown *TP53*^mutant^ NSCLC cells were determined by immunoblot analysis.

Emerging evidence suggests that pyroptosis is a novel pro-inflammatory form of lytic cell death which is distinct from apoptosis and may modulate the tumor microenvironment (TME) [53]. Our studies demonstrated that RG7388-induced pyroptosis was characterized by increased expression of cleaved GSDME (Fig. 2F and G), which can trigger cancer immune response [17, 20, 53]. To assess the impact of RG7388-induced pyroptosis on immune response in *TP53*^mutant^ lung cancer cells, we treated two *TP53*^mutant^ driven cell lines (HCC827 and PC9) with RG7388 and analyzed changes in immune-related gene expression by collecting RNA. RG7388 strongly affected global gene expression in both cell lines compared to control. KEGG classification analysis of these DEGs revealed the immune system as one of the most affected cellular processes in RG7388 treated *TP53*^mutant^ NSCLC (Supplementary Fig. 1F and G). The heatmap revealed the significant difference involved in immune system-related genes (Fig. 8A). The altered genes (including *IL-1*, *IL-6*, *IL-8*, *IL-11* and *CXCL2*) in the immune system category were validated by qRT-PCR in RG7388-treated HCC827 and PC9 cells (Fig. 8B). In line with GSEA analysis and qRT-PCR results, ELISA results demonstrated that higher levels of IL-6, IL-8, IL-1α and IL-1β were secreted by HCC827 and PC9 cells under RG7388 treatment (Fig. 8C). Briefly, GSDME mediated pyroptosis could alter TME by releasing cytokines in response to RG7388 treatment. In addition, RG7388 also decreased Programmed death ligand-1 (PD-L1) expression via the degradation of mutant p53 (Fig. 8D and Supplementary Fig. 3C). PD-L1 has been shown to play a well-characterized role in inhibiting anti-tumor immunity [54]. Taken together, RG7388 remodeled TME by the release of cytokines and the decrease in PD-L1 expression in *TP53*^mutant^ NSCLC.

## Discussion

In this study, we initially investigated the effects of RG7388 on *TP53*^mutant^ NSCLC, focusing on its impact on cell proliferation, cell death and the EGFR pathway modulation (Fig. 1). Our results demonstrated that RG7388 could serve as a potent anti-tumor agent against *TP53*^mutant^ NSCLC. Notably, RG7388-treated *TP53*^mutant^ NSCLC cells exhibited pyroptotic hallmarks including cellular swelling and membrane blebbing (Fig. 2A), which were ultrastructurally defined by TEM as plasma membrane pore formation (Fig. 2E). This pyroptotic morphology coincided with significant LDH release (Fig. 1F-I) and was molecularly corroborated by GSDME cleavage and caspase activation in western blot analyses (Fig. 2F-J). These findings collectively established RG7388 as a robust inducer of pyroptosis in this context. Mechanistically, RNA-seq analysis revealed NOXA as a key mediator of RG7388-induced cell death (Fig. 3A-C, Supplementary Fig. 1). To delineate NOXA’s functional role, we generated NOXA-deficient models and observed its essentiality in regulating both caspase-3-dependent apoptosis (primary cell death) and subsequent GSDME-mediated pyroptosis (secondary cell death) (Figs. 3 and 4 and Supplementary Fig. 2). Further investigation identified mitochondrial ROS (mtROS) accumulation as the upstream trigger for the JNK and p38 signaling pathways activation and NOXA upregulation. Intriguingly, while both JNK and p38 were activated by mtROS, only the p38 signaling mediated the mtROS-NOXA regulatory axis (Fig. 5). This critical role of the p38 signaling was functionally validated through pharmacological inhibition experiments, which significantly attenuated RG7388-induced apoptosis and pyroptosis (Fig. 6). Therefore, our results confirmed the key role of p-p38/NOXA axis as a major signaling pathway in RG7388 induced *TP53*^mutant^ NSCLC cell death. More importantly, the conserved correlation between NOXA and p-p38 verified in the NSCLC tissues (Fig. 7I), coupled with survival data linking their co-expression to poor prognosis (Fig. 7J), identified the p-p38/NOXA axis as a clinically actionable biomarker. These findings bridged molecular mechanism and clinical relevance, positioning the p-p38/NOXA axis as a therapeutically exploitable node.

The p53 tumor suppressor, recognized as “the guardian of the genome”, orchestrates apoptosis and chemotherapeutic responses through its transcriptional regulatory network [5]. Mouse double minute 2 homolog (MDM2) is the most well-known negative regulator of p53, which binds to p53 directly through its N-termini and inhibits p53 function by promoting proteasomal degradation of p53 or promoting export of p53 out of the cell nucleus [5, 7]. While both RG7388 and Nutlin3A act as MDM2 inhibitors that activate wild-type p53 in *TP53*^WT^ cancer [11, 35, 36], our study revealed critical functional divergence in *TP53*^mutant^ contexts. Strikingly, RG7388 reduced mutant p53 levels and induced cytotoxicity, whereas Nutlin3A paradoxically stabilized mutant p53 without triggering cell death (Supplementary Fig. 3A and B). This functional dichotomy aligned with one recent finding demonstrating RG7388’s efficacy in *TP53*^mutant^ pancreatic cancer cells [55], and is further substantiated by the preserved NOXA induction in *TP53* knockdown models (Supplementary Fig. 3C), confirming mutant p53-independent regulation. Beyond canonical p53 regulation, MDM2 exhibits pleiotropic functions, which include ubiquitination of non-p53 substrates, transcriptional modulation, DNA repair coordination, and regulation of mitochondrial respiration [7, 56]. Therefore, we proposed that RG7388’s distinct chemical architecture (Supplementary Fig. 4D and E) enabled dual mechanisms: (i) MDM2 inhibition-mediated respiratory complex I disruption leading to mtROS accumulation, and (ii) structural feature-dependent multi-target engagement. This poly-pharmacological property, shared by clinically successful kinase inhibitors [57–59], explained RG7388’s unique ability to bypass mutant p53 dependency – a critical advantage over Nutlin-3A’s singular wild-type p53-centric mechanism.

EGFR has been identified as a proto-oncogene that is mutationally activated in various human cancers. Notably, *EGFR* mutations are among the most common oncogenic drivers in NSCLC [4]. *EGFR*^mutant^ NSCLC is also a pivotal area of research in the field of precision oncology currently [60]. Over the past decade, multi-generations of tyrosine kinase inhibitors (TKIs) targeting wild-type or mutant EGFR have been approved for the treatment of patients with advanced NSCLC [34, 60, 61]. However, all NSCLC patients who initially benefit from EGFR-targeted therapies eventually develop TKIs resistance mainly due to genetically secondary *EGFR* mutations or activation of bypass survival tracks [62, 63]. This highlights the critical need for new treatment approaches to circumvent drug resistance for NSCLC patients with *EGFR* mutation refractory disease. Our research unveiled that RG7388 could specifically decrease EGFR protein expression and inhibit its downstream MEK/ERK and AKT/mTOR signaling independent of EGFR status (Fig. 1J). Results from preclinical or clinical studies demonstrate the potential of RG7388 as a novel therapy for solid and hematologic malignancies [64], which is now undergoing one new clinical testing (NCT04589845). Our research also highlighted the promising clinical utility of RG7388 as one potent anti-cancer agent dually targeting mutant p53 and mutant EGFR, which could attenuate TKIs resistance. However, the EGFR pathway or mutant p53 pathway was not the main regulating mechanism for the cytotoxic response to RG7388 in *TP53*^mutant^ NSCLC harboring wild-type or mutant EGFR. Co-mutations in *EGFR* and *TP53* occur with high frequencies, which reduce the effectiveness of TKIs and results in drug resistance by promoting genomic instability [4]. Elucidating mutant p53 and EGFR-independent cancer cell death pathways may yield useful information for the development of treatment strategies for chemo-refractory patients with *TP53* and *EGFR* co-mutations. Our mechanistic focus on p53/EGFR-independent death pathways addressed this critical knowledge gap. By elucidating RG7388’s ability to bypass conventional resistance mechanisms, this work provided a framework for targeting over 60% of *EGFR*^mutant^ NSCLC cases harboring concurrent *TP53* mutations [4].

Excessive ROS production causes oxidative damage to DNA, proteins, and lipids [65]. ROS-induced damage, if severe and/or persistent, can activate programmed cell death (PCD) through diverse modalities such as ferroptosis, cuproptosis, pyroptosis, necroptosis and apoptosis [24, 66]. As a major source of intracellular ROS, mtROS generation during chemotherapeutic responses primarily originates from electron leakage in the mitochondrial respiratory chain [24, 67]. However, the precise mechanisms linking mtROS to specific cell death mechanisms remain controversial, particularly regarding signaling cascade regulation. To dissect these pathways, we employed two established antioxidants including Fer-1 and NAC, both of which can alleviate ROS production and then inhibit ROS-triggered pyroptosis [52, 68–72]. In our research, increased mtROS levels and the NOXA/caspase-3 axis-mediated pyroptotic cell death were observed in *TP53*^mutant^ NSCLC cells after RG7388 treatment (Figs. 2–4 and Fig. 5A and B). ROS-mediated oxidative damage is considered to be an important part of the cytotoxicity of anti-cancer agents [66, 73]. It indicated that RG7388 might serve as one anti-cancer agent inducing mtROS generation and hold the potential to be used in target therapy for eradicating *TP53*^mutant^ NSCLC. However, the underlying mechanisms involved in mtROS activating the NOXA/caspase-3 axis have not yet been fully elucidated so far. Emerging evidence has demonstrated that ROS can act as secondary messengers and initiate various signaling cascades involved in the regulation and induction of both physiological and pathological cellular pathways [45, 46, 49–51, 65, 73]. Therefore, both NAC and Fer-1 as ROS scavengers were administrated to explore the signaling regulation mechanisms in the NOXA/caspase-3 axis-mediated cancer cell death. Our study demonstrated that both Fer-1 and NAC could reverse mtROS activated p-p38/NOXA axis which subsequently triggered pyroptosis in RG7388 treated *TP53*^mutant^ NSCLC cells (Figs. 5 and 6). These findings not only elucidated RG7388’s anti-tumor mechanisms but also suggested combinatorial strategies using ROS modulators to enhance therapeutic efficacy in *TP53*^mutant^ NSCLC.

Mitogen-activated protein kinases (MAPKs) are signal-transducing enzymes that serve as important mediators of the cellular response to various extracellular and intracellular signals including cytokines, growth factors, and various stresses [45]. Three main subgroups of the MAPK family have been identified: ERK, JNK and p38 [45]. Our study revealed that RG7388-induced mtROS activated both JNK and p38 pathways (Fig. 5F-K). However, it was only the p38 pathway, not the JNK pathway, that was required for the upregulation of NOXA in RG7388-treated *TP53*^mutant^ NSCLC cells (Fig. 5L-Q). Accordingly, inhibition of the p38 pathway rescued the cells from the NOXA/Caspase-3 axis-mediated cancer cell death (Fig. 6). These findings emphasize the crucial role of mtROS/p-p38 pathway in NOXA-mediated apoptosis and pyroptosis in *TP53*^mutant^ NSCLC cells when exposed to RG7388. Consistent with our results, the p38 signaling is known to be important in transducing oxidative stress signals [45, 46]. However, the mechanisms involved in the regulation of NOXA by p-p38 were not elucidated in the current study. As shown in Fig. 5C–E, RG7388 induced substantial upregulation of gene sets related to the AP-1 signaling including *JUND*, *JUNB*, *JUN* and *FOS*, which are downstream targets of MAPKs of p38 and JNK signaling pathways [45]. We then analyzed *PMAIP1* (NOXA) gene promoters by using the online JASPAR resource and found multiple putative FOS or JUND transcriptional binding sites in the promoter. Moreover, the *PMAIP1* gene encoding NOXA protein was significantly upregulated by RG7388 treatment according to our RNA-seq results (Fig. 3B and C). It has been reported that ROS accumulation results in the upregulation of NOXA primarily on a transcriptional level [44]. Our findings, combined with the known mtROS/p-p38 pathway activating the AP-1 complex, suggested a working model wherein the mtROS/p-p38/AP-1 complex might transcriptionally activate *PMAIP1* gene expression. While our data establish the p-p38/AP-1/NOXA axis as central to RG7388’s action, two key questions require resolution: First, how the p38 signaling specifically coordinates with AP-1 components to transactivate *PMAIP1*, given that both JNK and p38 can regulate AP-1 activity [45]. Second, whether post-transcriptional mechanisms (e.g., NOXA protein stabilization) contribute to pathway amplification. Future studies employing chromatin immunoprecipitation (ChIP) and AP-1 dominant-negative mutants could delineate these regulatory interactions.

Furthermore, to delineate the essential role of caspase-3 in GSDME cleavage in RG7388-induced pyroptosis, we manipulated the activity of caspase-3 by administering pan-caspase inhibitor Z-VAD-FMK, caspase-8 inhibitor Z-IETD-FMK, caspase-3 inhibitor Z-DEVD-FMK or caspase-3 activator SM164. Consistent with canonical pyroptosis mechanism, inhibitor treatments (Z-VAD-FMK, Z-IETD-FMK or Z-DEVD-FMK) attenuated RG7388-induced GSDME cleavage (Fig. 4G-J), whereas SM164 potentiated these effects (Fig. 4N-Q). RG7388-induced LDH release was also suppressed after Z-DEVD-FMK treatment (Fig. 4K-M). In addition, pharmacological inhibition of p38 pathway or G3BP2 KO could efficiently attenuate the cleavage of GSMDE by inhibiting the NOXA/caspase-3 axis, further conforming activated caspase-3 as a key executor in the cleavage process of GSDME (Fig. 6 and Supplementary Fig. 2). In the classical pyroptosis process, GSDME has long been regarded as one substrate that can be cleaved by activated caspase-3, which is in consistent with our results (Fig. 4 and Supplementary Fig. 2). It has been documented that mtROS can activate GSDME-mediated pyroptosis [23]. However, the underlying mechanisms remain elusive. Here, we were the first to report the novel mechanism that mtROS could activate the cleavage of GSDME via the p-p38/NOXA/Caspase-3 pathway in RG7388-treated *TP53*^mutant^ NSCLC cells. This discovery extended NOXA’s role beyond apoptosis, positioning it as a pyroptosis regulator in p53-deficient contexts (Fig. 6L). These results suggested that NOXA might be targeted in *TP53*^mutant^ NSCLC to develop new personalized treatment strategies for NSCLC patients.

Finally, while our mechanistic studies focused on *TP53*^mutant^ NSCLC models, the clinical prognostic significance of the p-p38/NOXA axis was observed in an unstratified NSCLC cohort. This apparent paradox yielded two non-exclusive interpretations: First, the conserved correlation between p-p38 and NOXA protein levels across clinical specimens (Fig. 7I) strongly supported the biological relevance of this axis in human NSCLC. Second, the survival association (Fig. 7J) might arise from either (1) predominant contribution of the *TP53*^mutant^ subgroup (representing >50% of cases [6]), or (2) p53-independent NOXA regulation mechanisms operative in *TP53*^WT^ contexts [6, 27, 28, 32]. Notably, *TP53*^mutant^ tumors’ characteristic oxidative stress [6, 28] - the key driver of ROS/p-p38/NOXA activation - likely amplified prognostic power in this subgroup, consistent with our experimental models showing mutant-specific RG7388 sensitivity. Nevertheless, the absence of *TP53* status annotation in clinical datasets currently precluded definitive stratification. Future investigations should: (1) Validate the axis in *TP53*-stratified cohorts using multi-center data, and (2) Map NOXA regulatory networks in *TP53*^WT^ NSCLC through single-cell omics.

In summary, our study established three key advances in *TP53*^mutant^ NSCLC therapy: First, RG7388 induced dual-phase cell death through sequential apoptosis and pyroptosis, mediated by the ROS/p-p38/NOXA/caspase-3/GSDME axis. Second, combinatorial treatment with SM164 enhanced RG7388’s efficacy via caspase-3/GSDME activation, representing a novel EGFR-status-independent therapeutic strategy. Third, RG7388 demonstrated multi-modal anti-tumor activity by: (1) degrading mutant p53 and EGFR, (2) remodeling the inflammatory TME through pyroptosis-induced immunomodulation [20, 53] and (3) activating oxidative stress-dependent death pathways. These findings carried important clinical implications: The ROS/p-p38/NOXA axis emerged as a druggable target for *TP53*^mutant^ NSCLC, particularly relevant given the high prevalence of *TP53* mutations in NSCLC [4]. Moreover, the RG7388/SM164 combination addressed a critical need for therapies overcoming EGFR-TKIs resistance in *TP53*-co-mutant cases. Notably, pyroptosis-mediated TME remodeling suggested potential synergy with immunotherapies - a hypothesis warranting investigation through PD-1/PD-L1 combination trials. Future studies should prioritize: (1) Characterization of pyroptosis-induced cytokine profiles and their impact on immune cell infiltration, (2) In vivo validation of TME remodeling using humanized mouse models, and (3) Biomarker development for patient stratification based on p-p38/NOXA activation status.

## Supplementary information

Supplementary Table

Supplementary Figure legends

Supplementary Figure 1.

Supplementary Figure 2.

Supplementary Figure 3.

Supplementary Figure 4.

Original Data

## Data Availability

Complete RNA sequencing (RNA-seq) data have been deposited in the Gene Expression Omnibus (GEO) database (accession no. GSE287938). All other data that support the findings of this study are available from the corresponding author upon reasonable request.
