# Supplementary material for: Repurposing MDM2 inhibitor RG7388 for TP53-mutant NSCLC: a p53-independent pyroptotic mechanism via ROS/p-p38/NOXA/caspase-3/GSDME axis
Source: Cell Death Dis. 2025 Jun 17;16(1):452. doi: 10.1038/s41419-025-07770-2 (PMC12170848; doi:10.1038/s41419-025-07770-2)
Supplement: Supplementary file 1 — Supplementary Table [file 41419_2025_7770_MOESM1_ESM.docx]

Table S1. Primer Sequence.

| Gene | Forward primer (5′-3′) | Reverse primer (5′-3′) |
| --- | --- | --- |
| *IL-1* | AACCTCTTCGAGGCACAAGG | AGATTCGTAGCTGGATGCCG |
| *IL-6* | ACATAGACGGATCACAGTGC | AAAATGGGGGAGGATGGCTG |
| *IL-8* | GAAGTTTTTGAAGAGGGCTGAGA | ACCAAGGCACAGTGGAACAA |
| *IL-11* | CAGATACAGCTGTCGCCCCT | AGGTAGGACAGTAGGTCCGC |
| *CXCL-2* | AGATCAATGTGACGGCAGGG | TCTCTGCTCTAACACAGAGGGA |
| *GAPDH* | AATGGGCAGCCGTTAGGAAA | GCGCCCAATACGACCAAATC |
