# Supplementary material for: Repurposing MDM2 inhibitor RG7388 for TP53-mutant NSCLC: a p53-independent pyroptotic mechanism via ROS/p-p38/NOXA/caspase-3/GSDME axis
Source: Cell Death Dis. 2025 Jun 17;16(1):452. doi: 10.1038/s41419-025-07770-2 (PMC12170848; doi:10.1038/s41419-025-07770-2)
Supplement: Supplementary file 2 — Supplementary Figure legends [file 41419_2025_7770_MOESM2_ESM.docx]

Supplementary Fig. 1. RNA-seq and bioinformatic analysis in RG7388 treated HCC827 and PC9 cells.

(A-C) Heat map representing the most significantly regulated genes detected in RNA-seq analysis with control and RG7388 treated HCC827 and PC9 cells. Gene expressions were normalized with row Z-score. The upregulated (B) and downregulated (C) DEGs were also presented as a Venn Diagram.

(D and E) Gene set enrichment analysis (GSEA) using KEGG terms was performed between HCC827 (D) or PC9 (E) cells treated by RG7388 compared to control cells, and the top significant hits are shown.

(F and G) KEGG pathway enrichment analysis was performed between HCC827 (F) or PC9 (G) cells treated by RG7388 compared to control cells.

Supplementary Fig. 2. G3BP2 depleting reversed cancer cell death via inhibiting NOXA/Caspase-3 axis.

(A) HCC827 cells were transfected with siRNAs against *G3BP1* or *G3BP2* genes. The protein levels of both genes were detected by Immunoblot.

(B) HCC827 cells transfected with siRNA mediated knockdown of G3BP1 (siRNA-G3BP1), G3BP2 (siRNA-G3BP2) or control (siRNA-Control) were treated by 60 μM RG7388 for 6 h. Immunoblot analysis of cleaved PARP, cleaved caspase-3, GSDME and cleaved GSDME protein. β-Actin or GAPDH served as a loading control.

(C) HCC827 cells transfected with siRNA mediated knockdown of G3BP1 (siRNA-G3BP1), G3BP2 (siRNA-G3BP2) or control (siRNA-Control) were treated by 60 μM RG7388 for 6 h. Immunoblot analysis of NOXA protein. β-Actin served as a loading control.

(D) Immunoblot analysis of the effect of G3BP1 or G3BP2 knockout in H23 cells by using the Crispr-Cas9 gene editing technique.

(E) H23 cells were stably transfected with CRISPR / Cas9 mediated knock out of G3BP1 (sgRNA-G3BP1), G3BP2 (sgRNA-G3BP2) or control (sgRNA-Control) were treated by 60 μM RG7388 for 6 hours. Immunoblot analysis of cleaved PARP, cleaved caspase-3, cleaved GSDME and NOXA proteins. β-Actin served as a loading control.

(F) Cell morphology via optical microscopy in G3BP2 KO H1975 cells induced by RG7388 (60 μM) for 6 h.

Supplementary Fig. 3. Specifical downregulation of Mutant p53 by RG7388 did not affect the NOXA expression.

(A) Immunoblot analysis of p53, cleaved PARP and cleaved Caspase-3 proteins in HCC827 cells upon treatment with increasing concentrations of RG7388 or Nutlin3A for 24 hours.

(B) Immunoblot analysis of p53, cleaved PARP and cleaved Caspase-3 proteins in PC9 and H23 cells upon treatment with increasing concentrations of RG7388 or Nutlin3A for 24 hours.

(C) Protein levels of p53 and NOXA in *TP53* knockdown *TP53*^mutant^ NSCLC cells were determined by immunoblot analysis.

(D and E) The chemical structure of RG7388 (D) or Nutlin-3A (E).

Supplementary Fig. 4. The expression of p-p38 MAPK and NOXA in clinical NSCLC patients’ tissues.

(A and B) Immunohistochemical staining and scoring of p-p38 MAPK in tumor tissues (T) and paired noncancerous normal tissues (N) from NSCLC patients (n = 90) using tissue microarray (A). NSCLC tissue sections were quantitatively scored according to the percentage of positive cells and staining intensity (B). Scale bar: 5000 µm.

(C and D) Immunohistochemical staining and scoring of NOXA in tumor tissues (T) and paired noncancerous normal tissues (N) from NSCLC patients (n = 90) using tissue microarray (C). NSCLC tissue sections were quantitatively scored according to the percentage of positive cells and staining intensity (D). Scale bar: 5000 µm.

(E) The expression profile of NOXA in NSLCL tumors and normal tissues (T: n = 483, N: n = 347). One-way ANOVA (*p < 0.05).

(F) Kaplan Meier analysis of overall survival in NSCLC patients (n = 478) from TCGA database based on *PMAIP1* (NOXA) gene expression. The data were analyzed by log-rank test, the value of hazard ratio (HR) and log-rank p-value are shown.
