# Supplementary figures and images for: Repurposing MDM2 inhibitor RG7388 for TP53-mutant NSCLC: a p53-independent pyroptotic mechanism via ROS/p-p38/NOXA/caspase-3/GSDME axis
Source: Cell Death Dis. 2025 Jun 17;16(1):452. doi: 10.1038/s41419-025-07770-2 (PMC12170848; doi:10.1038/s41419-025-07770-2)

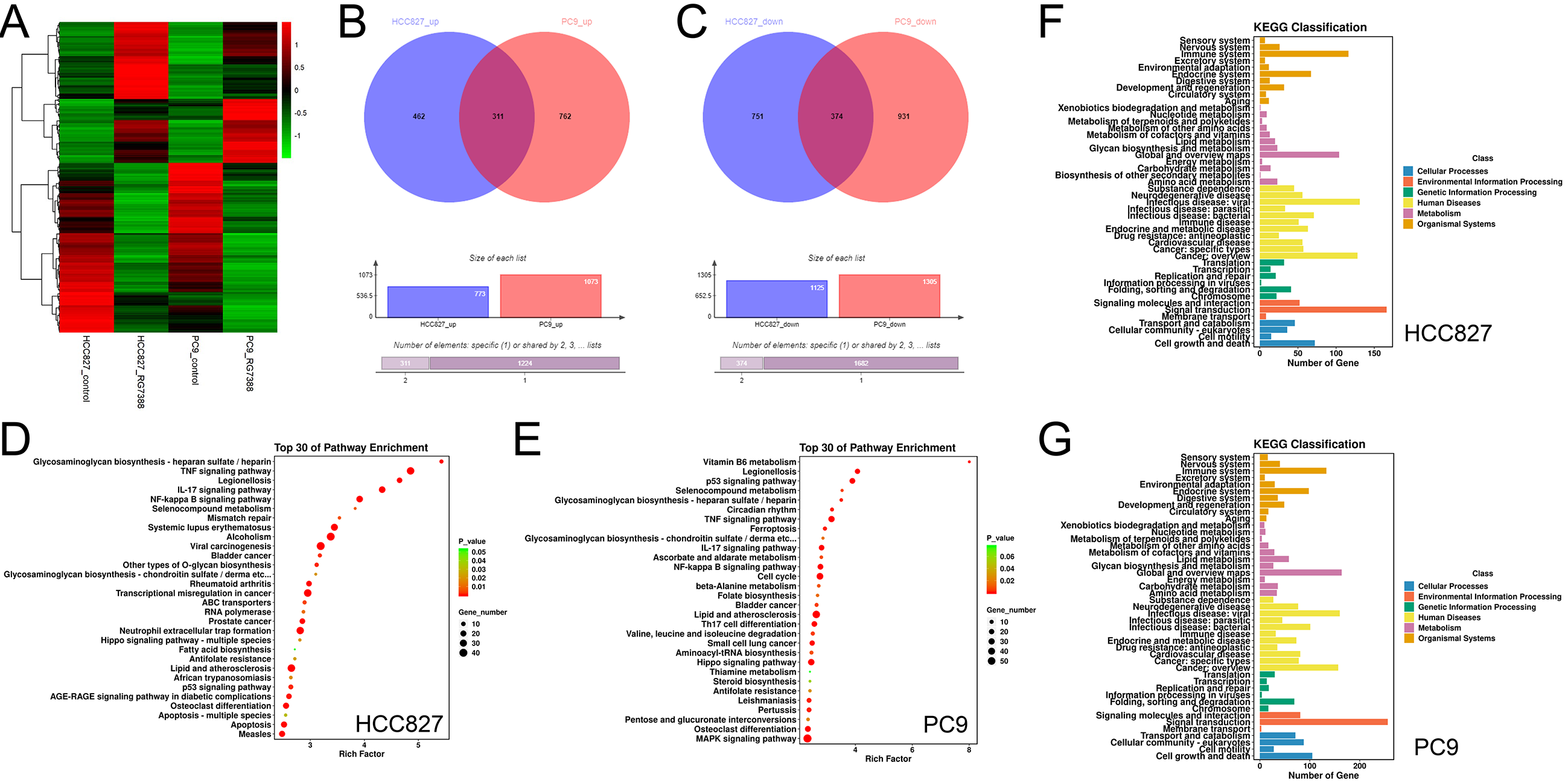

Supplement: Supplementary file 3 — Supplementary Figure 1. [file 41419_2025_7770_MOESM3_ESM.tif]

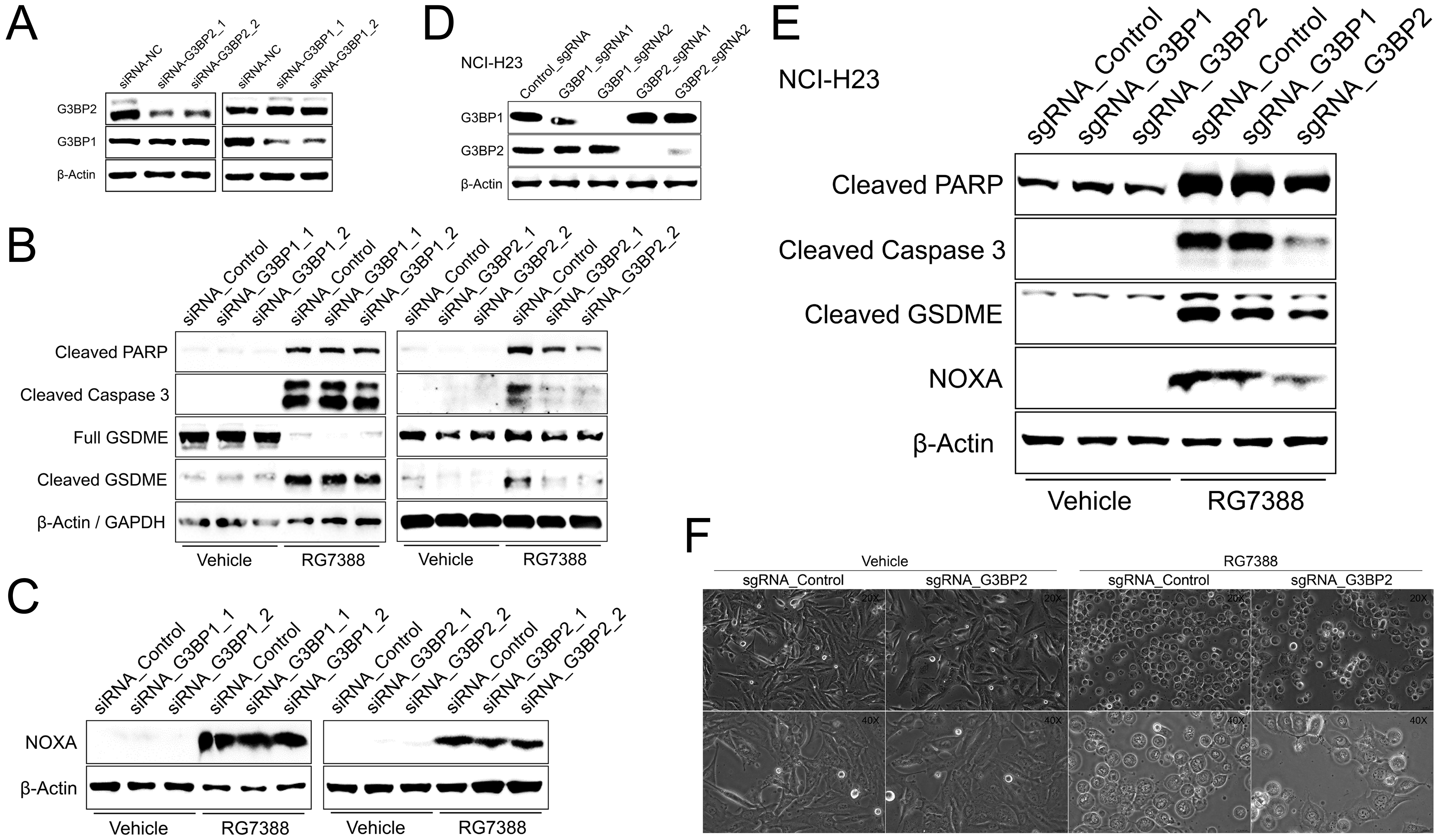

Supplement: Supplementary file 4 — Supplementary Figure 2. [file 41419_2025_7770_MOESM4_ESM.tif]

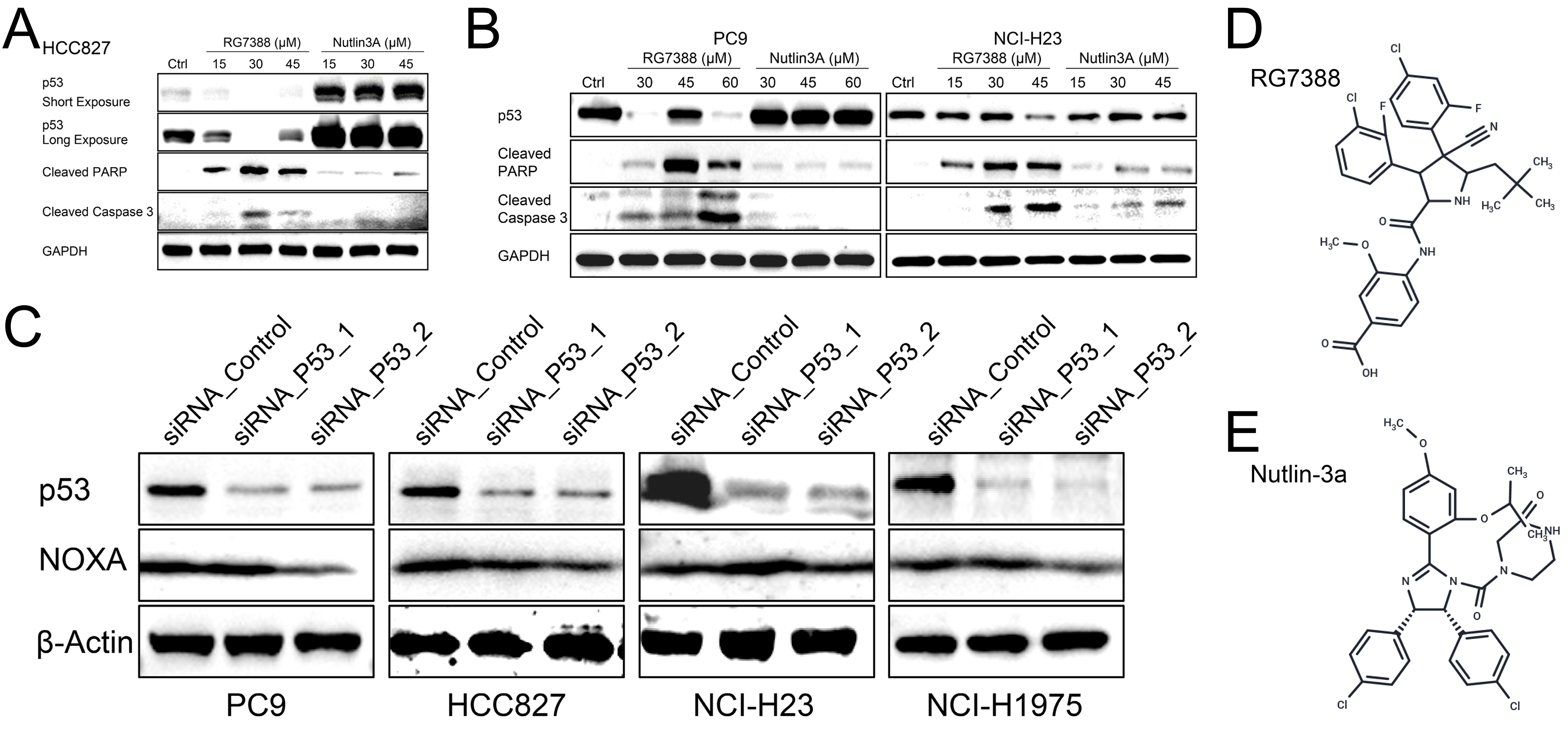

Supplement: Supplementary file 5 — Supplementary Figure 3. [file 41419_2025_7770_MOESM5_ESM.tif]

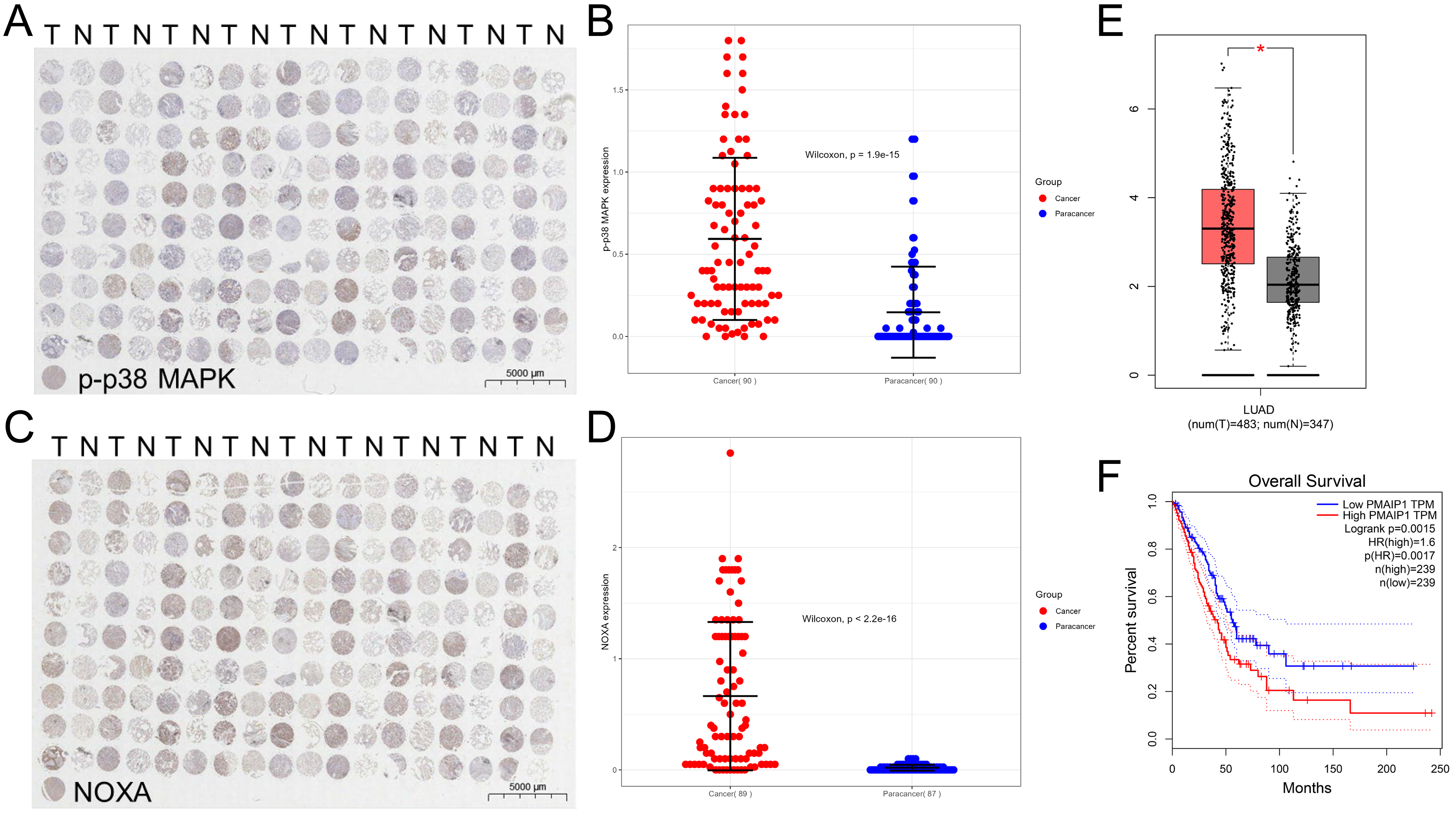

Supplement: Supplementary file 6 — Supplementary Figure 4. [file 41419_2025_7770_MOESM6_ESM.tif]
