# Supplementary material for: Repurposing MDM2 inhibitor RG7388 for TP53-mutant NSCLC: a p53-independent pyroptotic mechanism via ROS/p-p38/NOXA/caspase-3/GSDME axis
Source: Cell Death Dis. 2025 Jun 17;16(1):452. doi: 10.1038/s41419-025-07770-2 (PMC12170848; doi:10.1038/s41419-025-07770-2)
Supplement: Supplementary file 7 — Original Data [file 41419_2025_7770_MOESM7_ESM.pdf]

Fig.1J

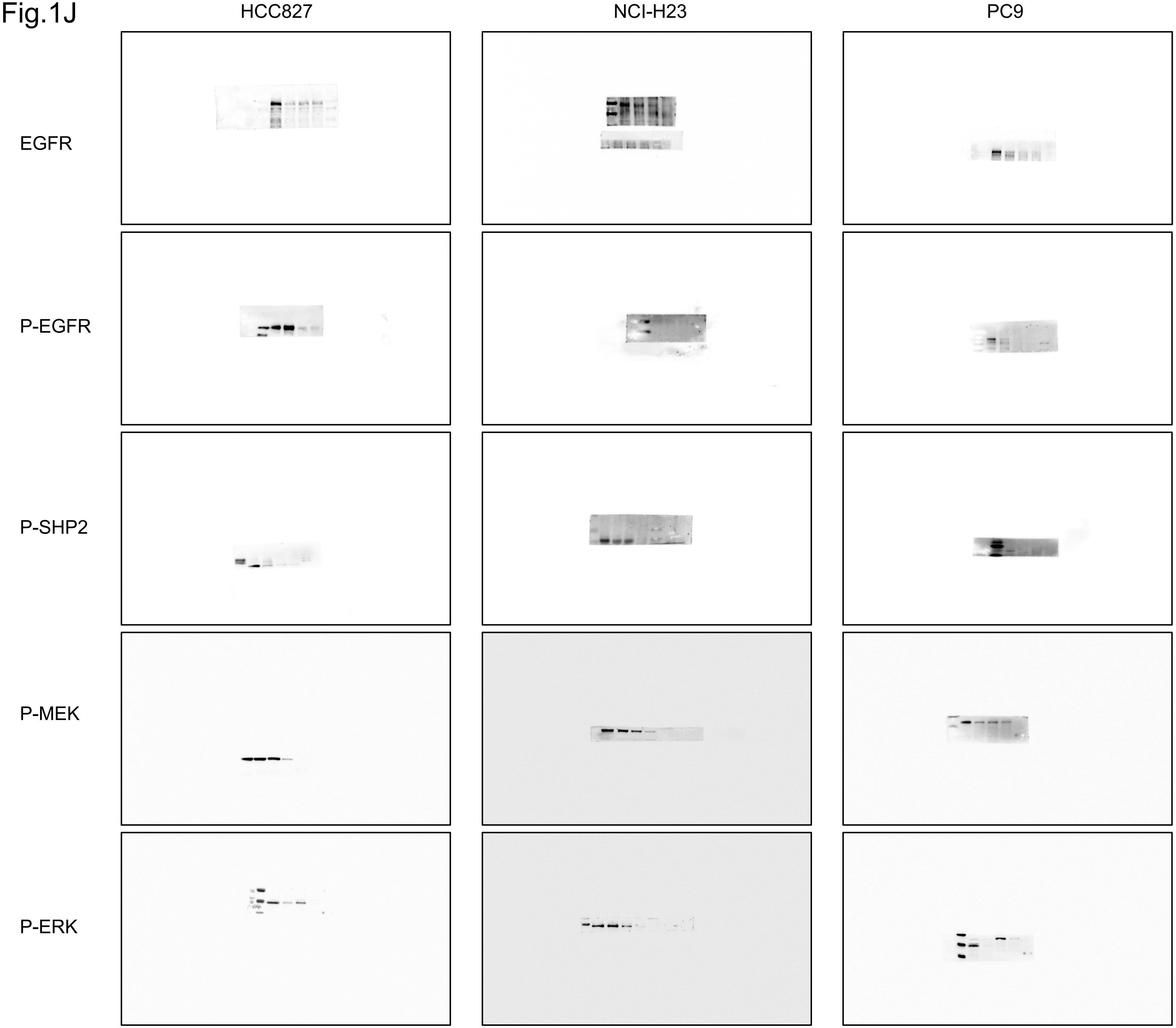

Fig.1J

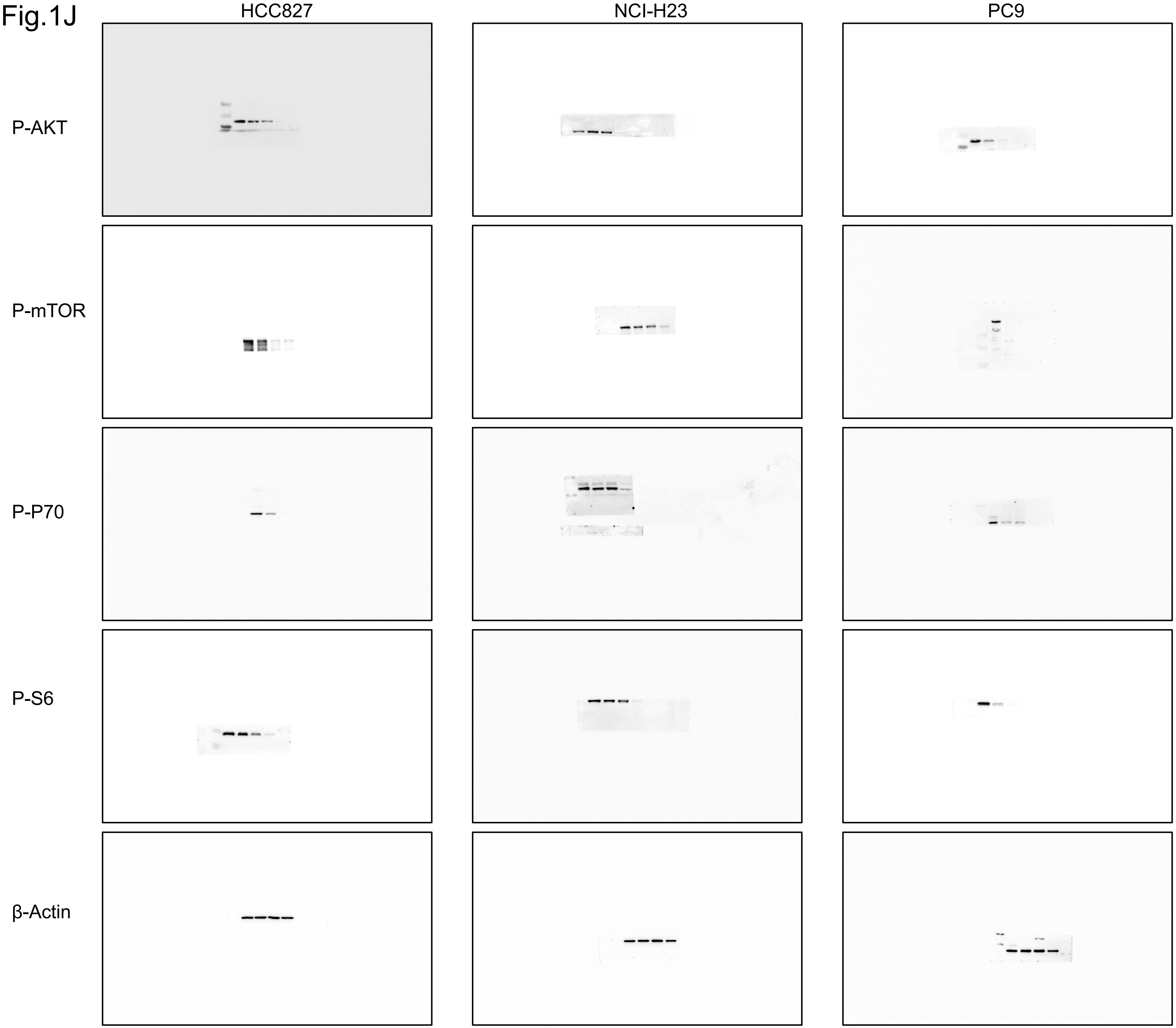

Fig.2F

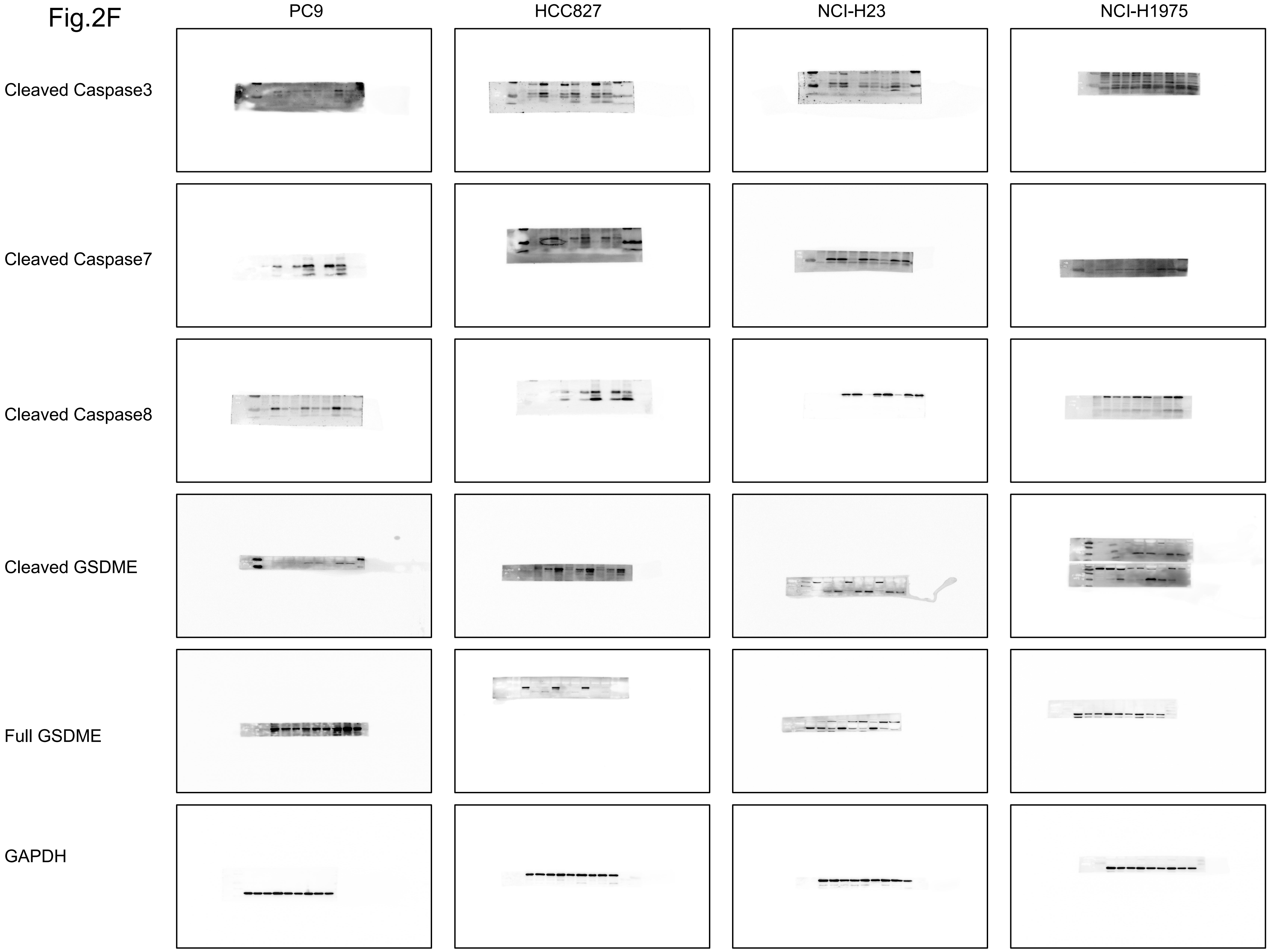
PC9
HCC827
NCI-H23
NCI-H1975
Cleaved Caspase3
Cleaved Caspase7
Cleaved Caspase8
Cleaved GSDME
Full GSDME
GAPDH

Fig.2G

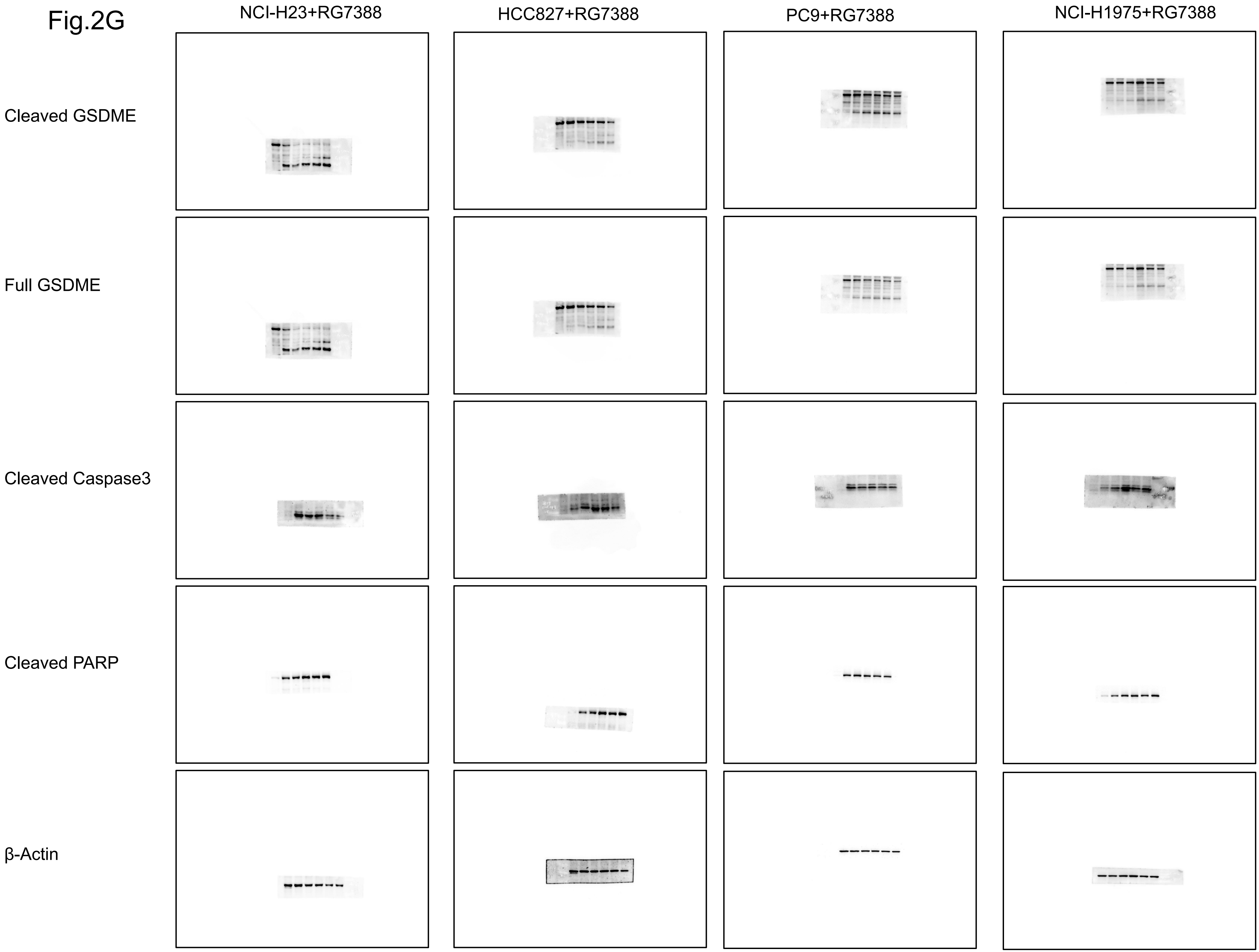
NCI-H23+RG7388
HCC827+RG7388
PC9+RG7388
NCI-H1975+RG7388
Cleaved GSDME
Full GSDME
Cleaved Caspase3
Cleaved PARP
β-Actin

Fig.3D

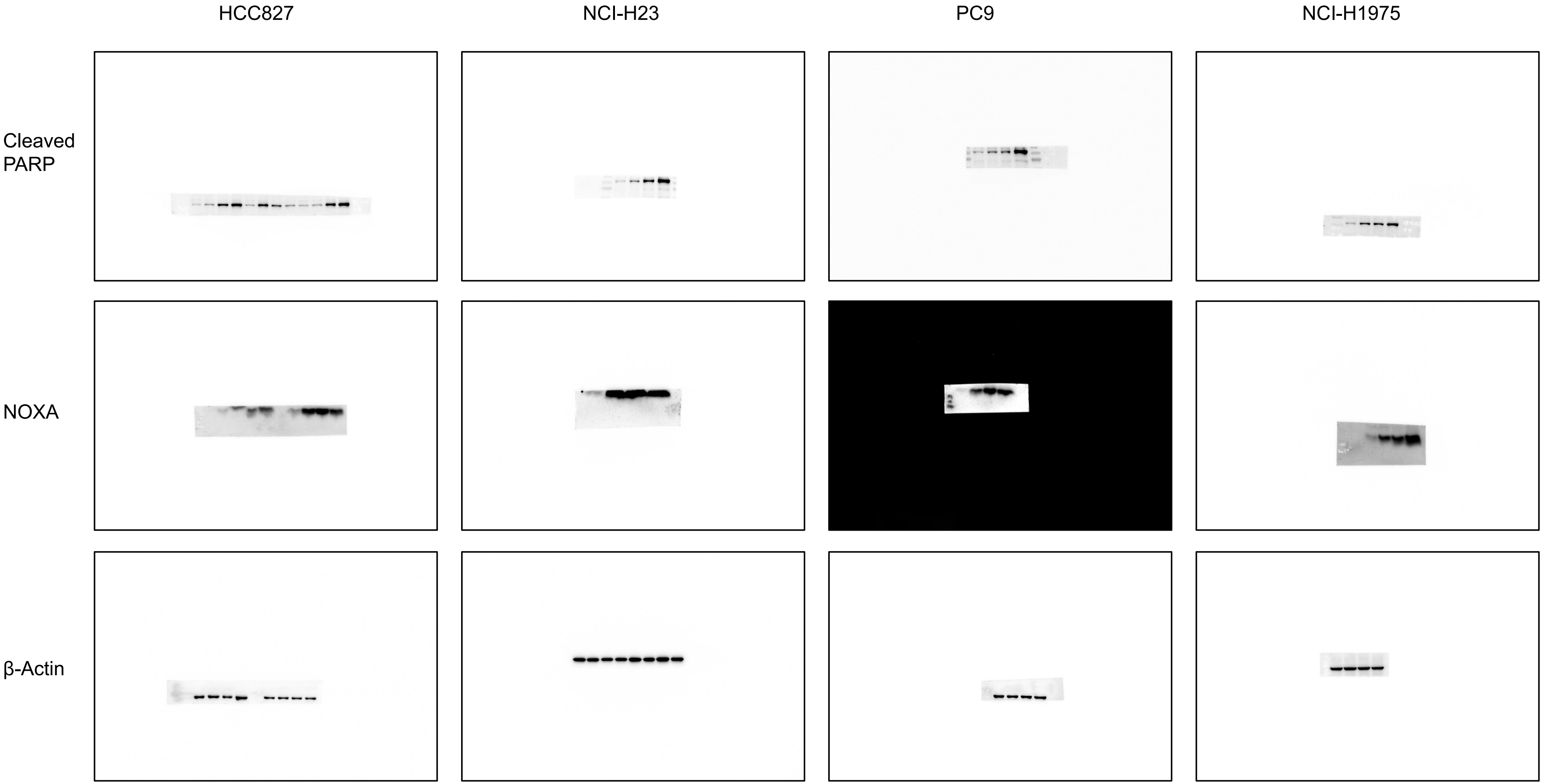

Fig.3G

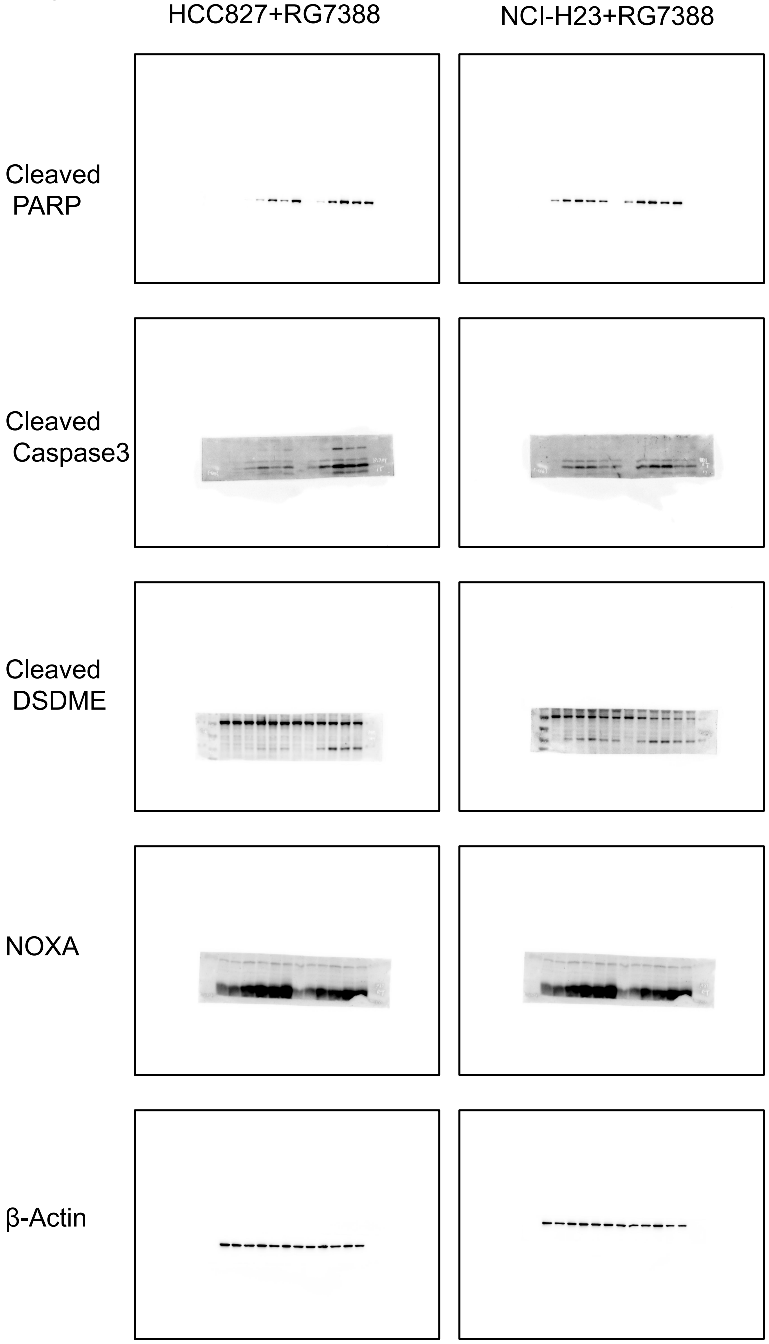

Fig.3J

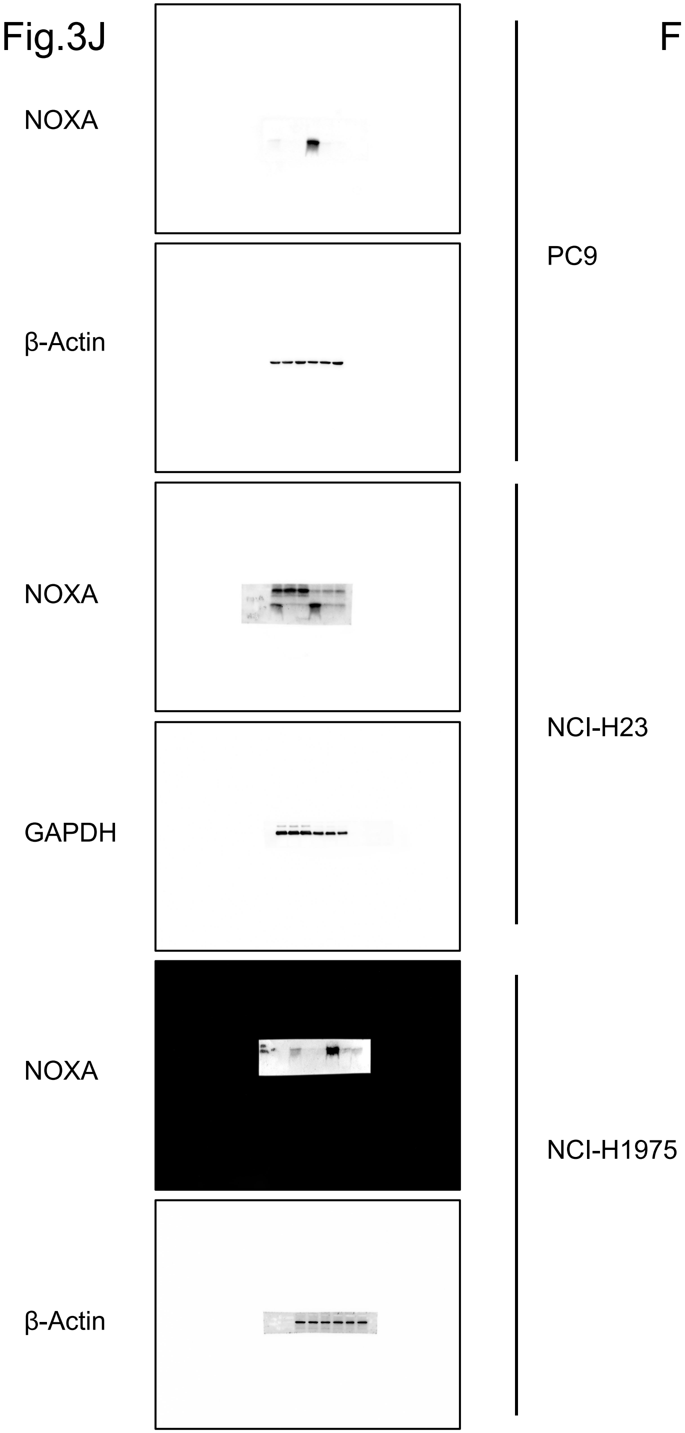

Fig.3K

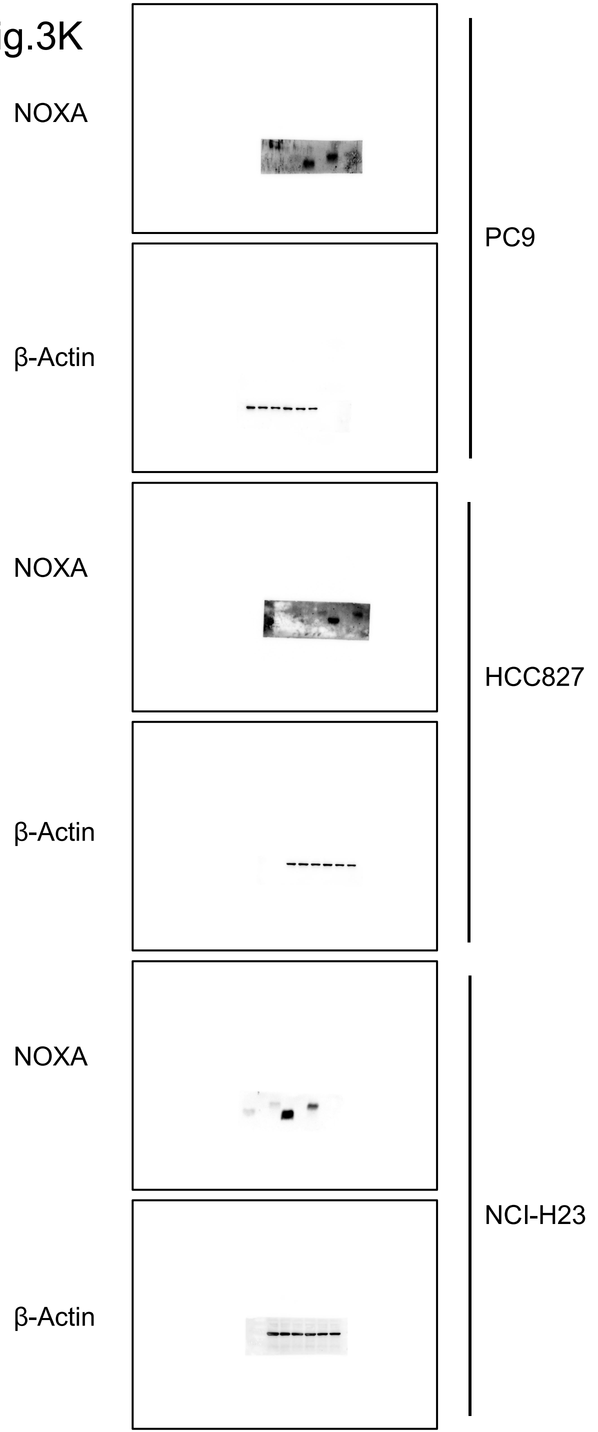

Fig.3L

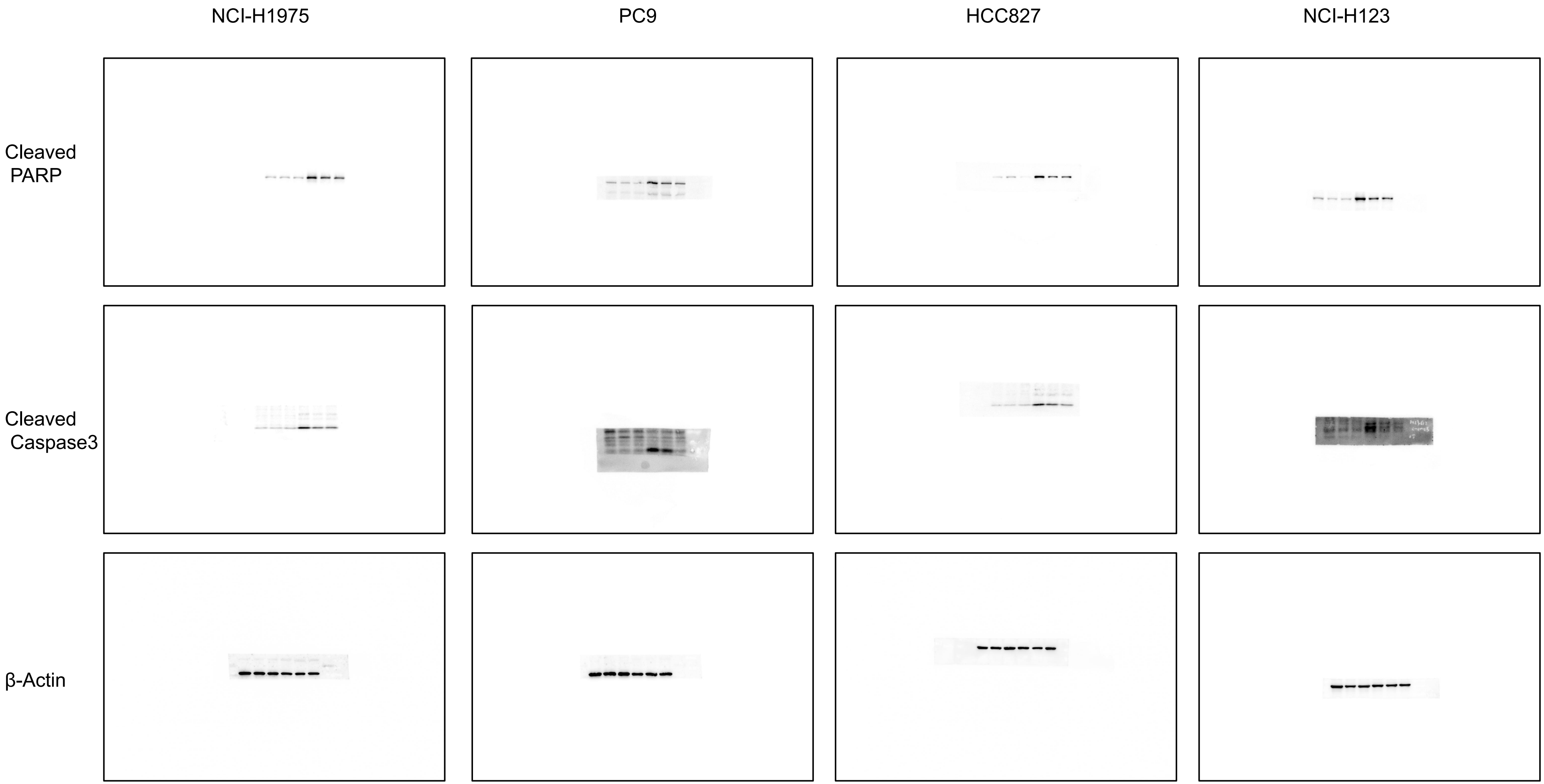
NCI-H1975
PC9
HCC827
NCI-H123
Cleaved PARP
Cleaved Caspase3
β-Actin

Fig.3P

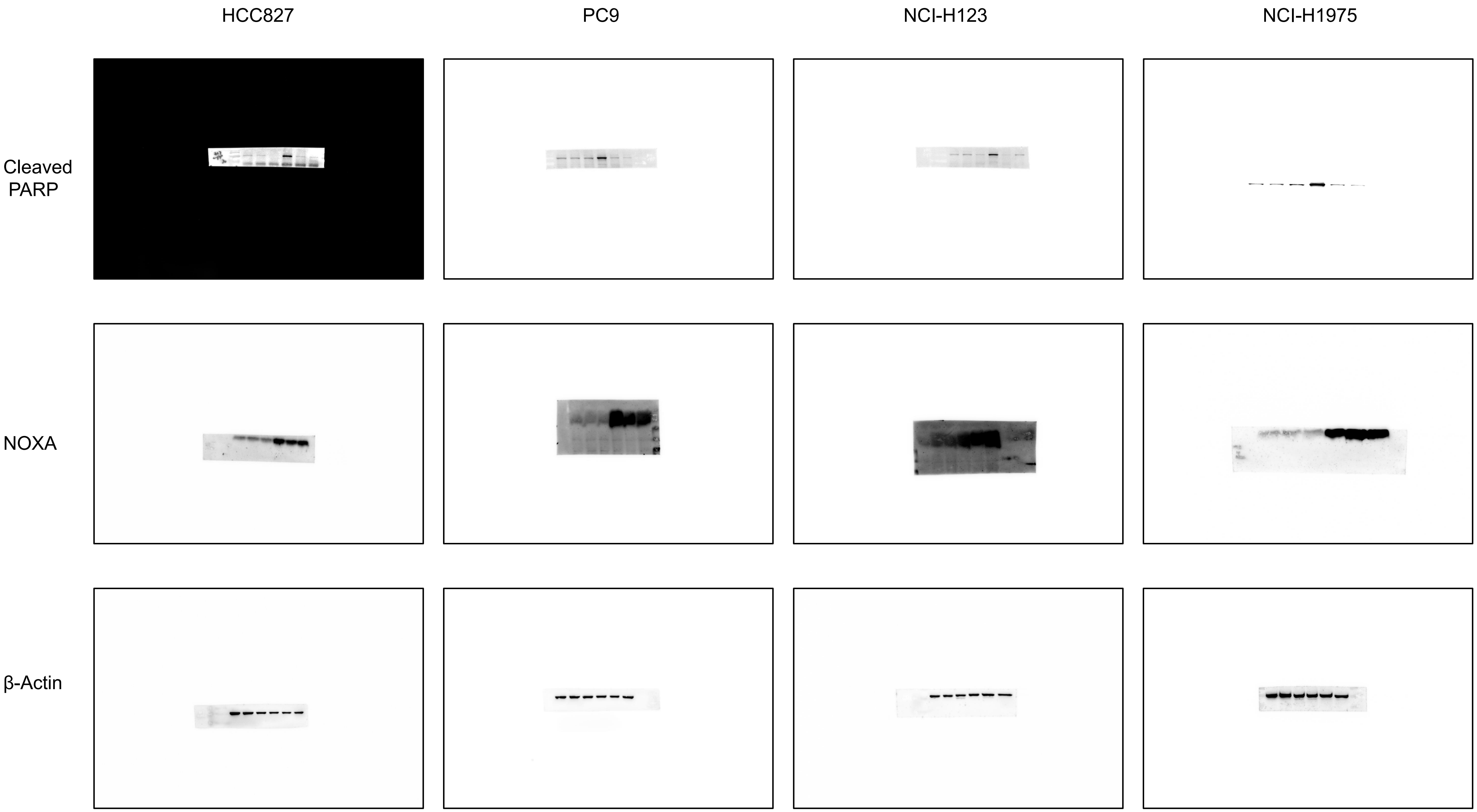

Fig.4B

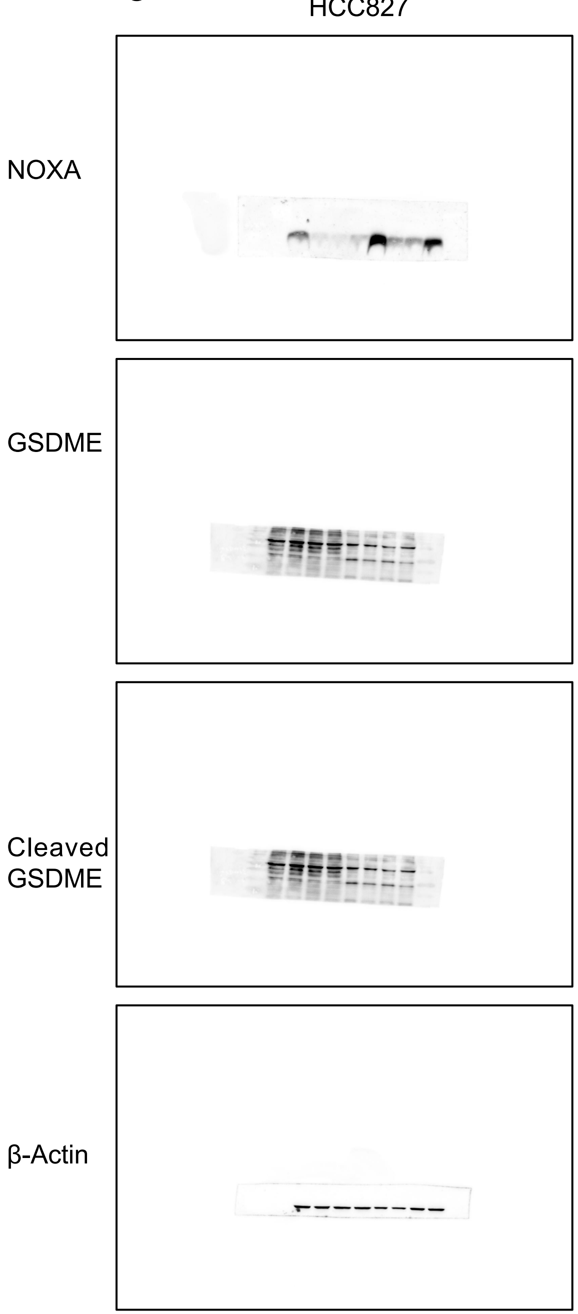
HCC827
NOXA
GSDME
Cleaved GSDME
β-Actin

Fig.4C

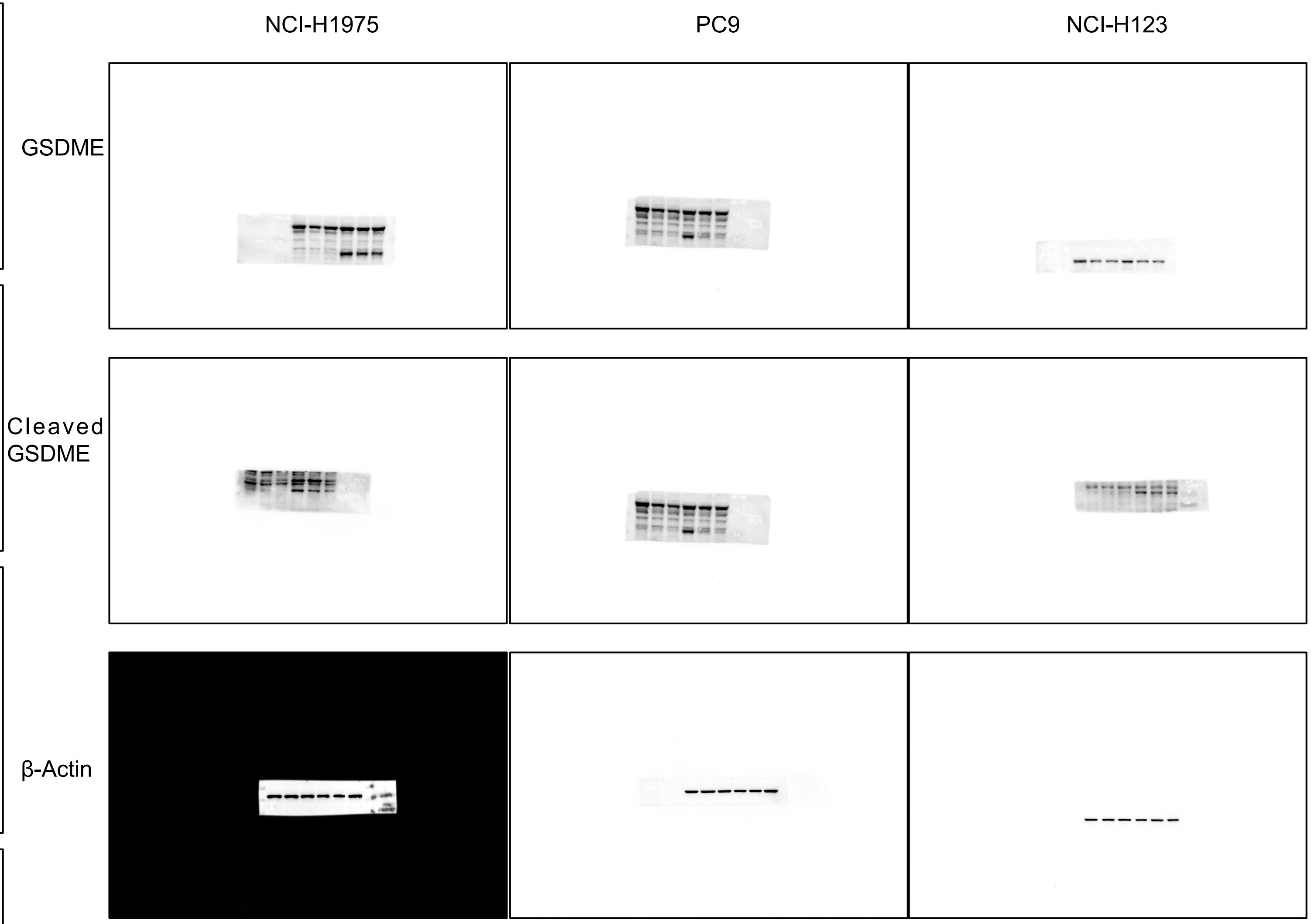
NCI-H1975
PC9
NCI-H123
GSDME
Cleaved GSDME
β-Actin

Fig.4G

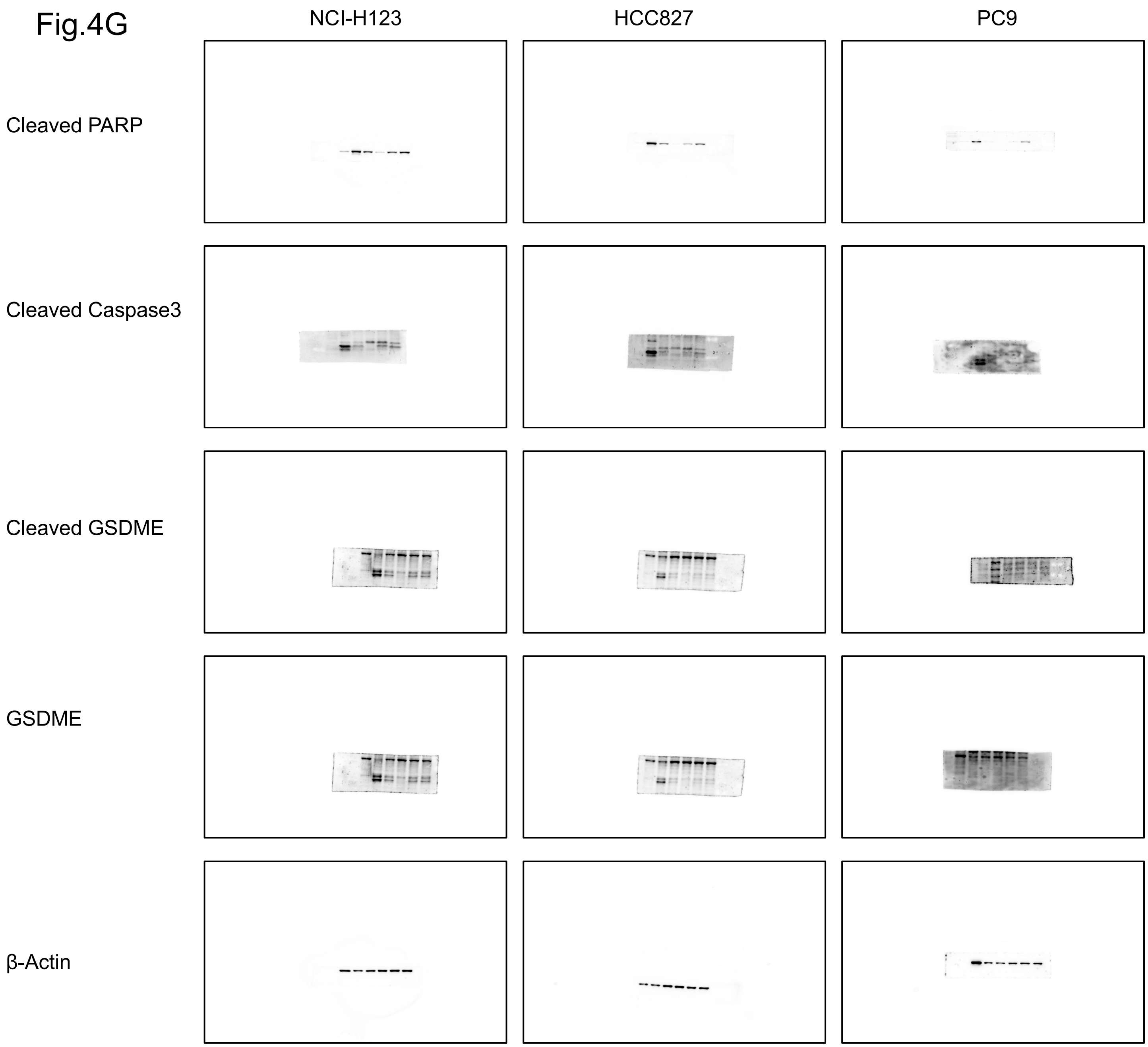
NCI-H123
HCC827
PC9
Cleaved PARP
Cleaved Caspase3
Cleaved GSDME
GSDME
β-Actin

Fig.4N

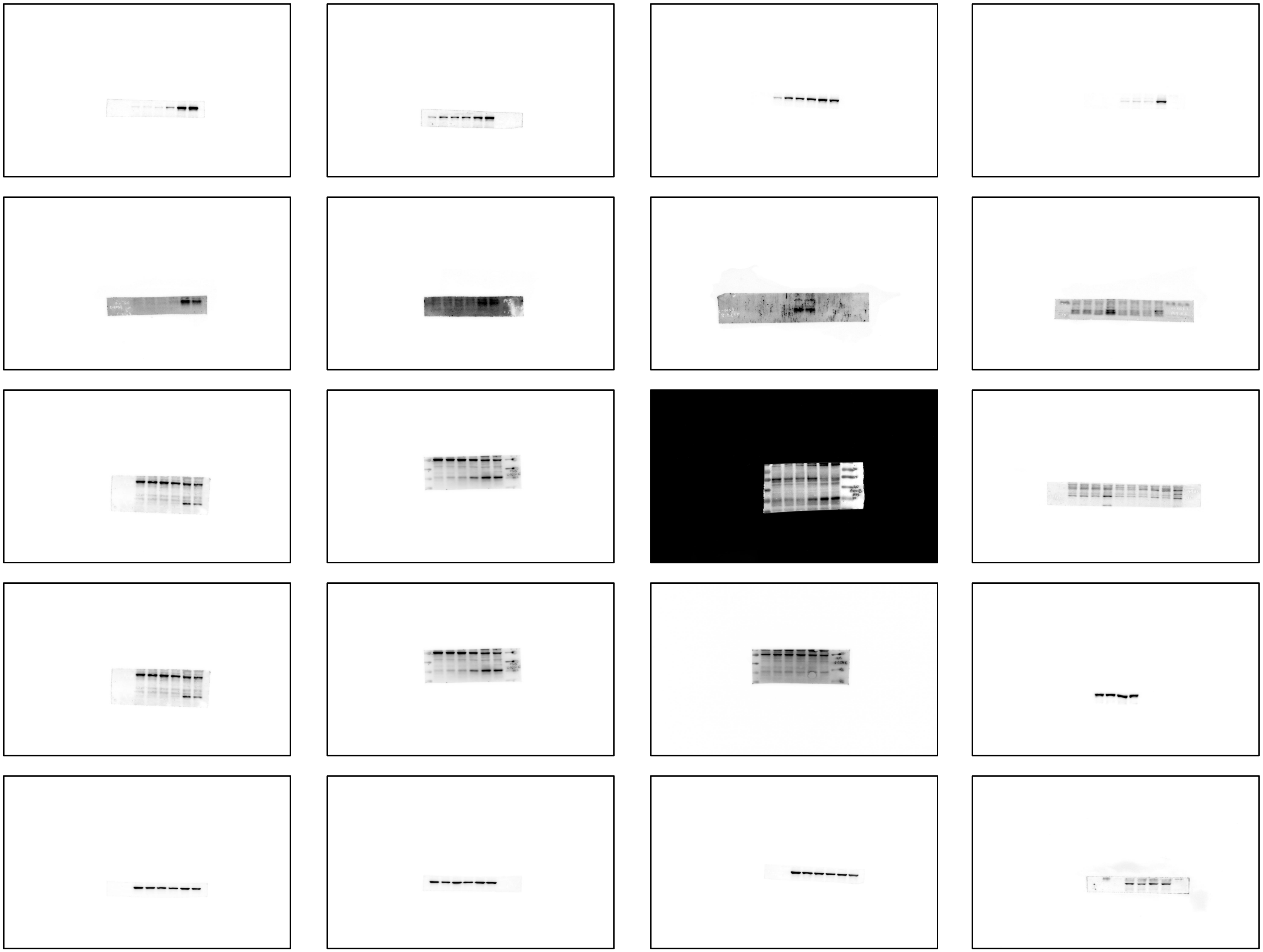
HCC827
NCI-H1975
NCI-H123
PC9
Cleaved PARP
Cleaved Caspase3
Cleaved GSDME
Full GSDME
β-Actin

Fig.5F

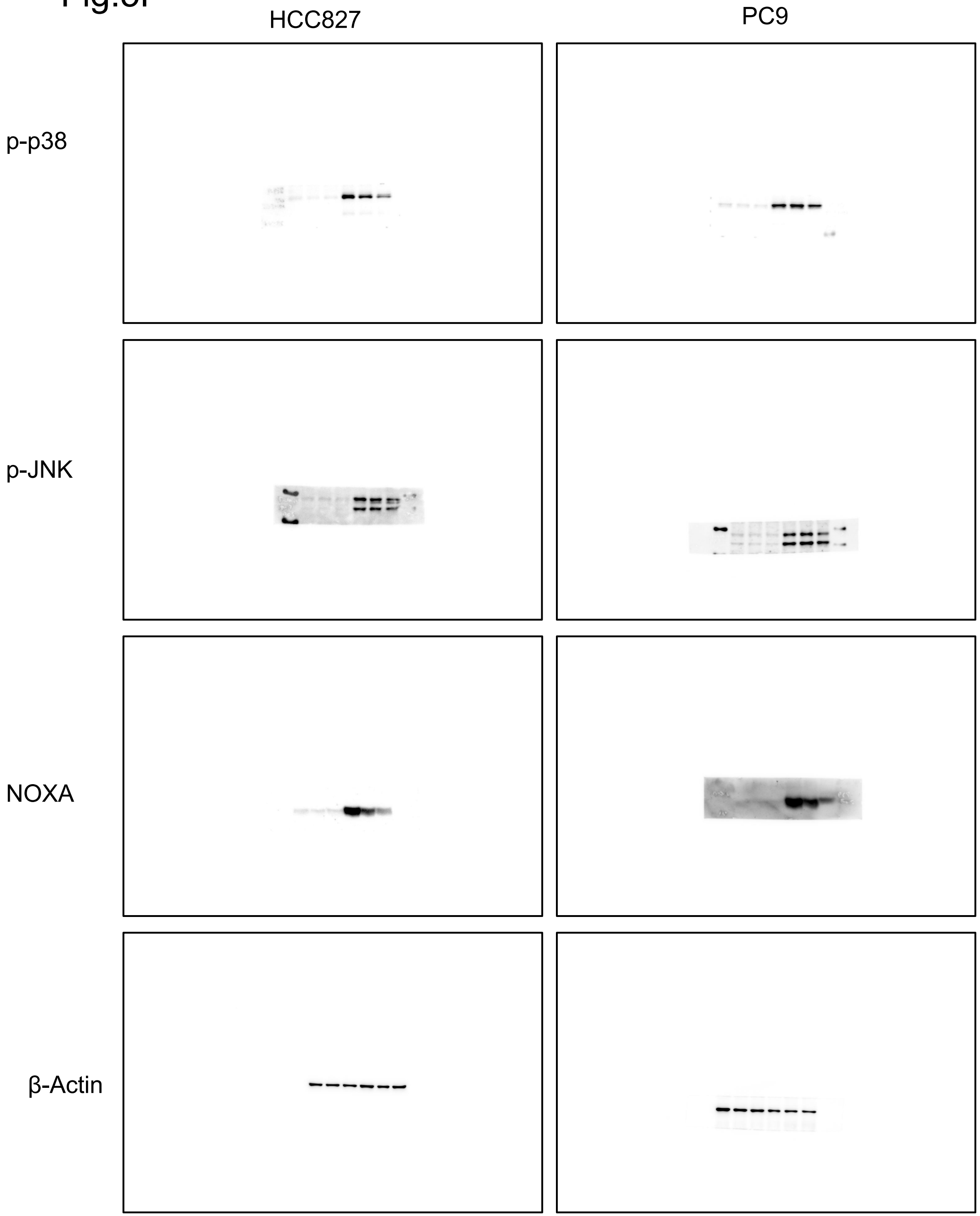

Fig.5I

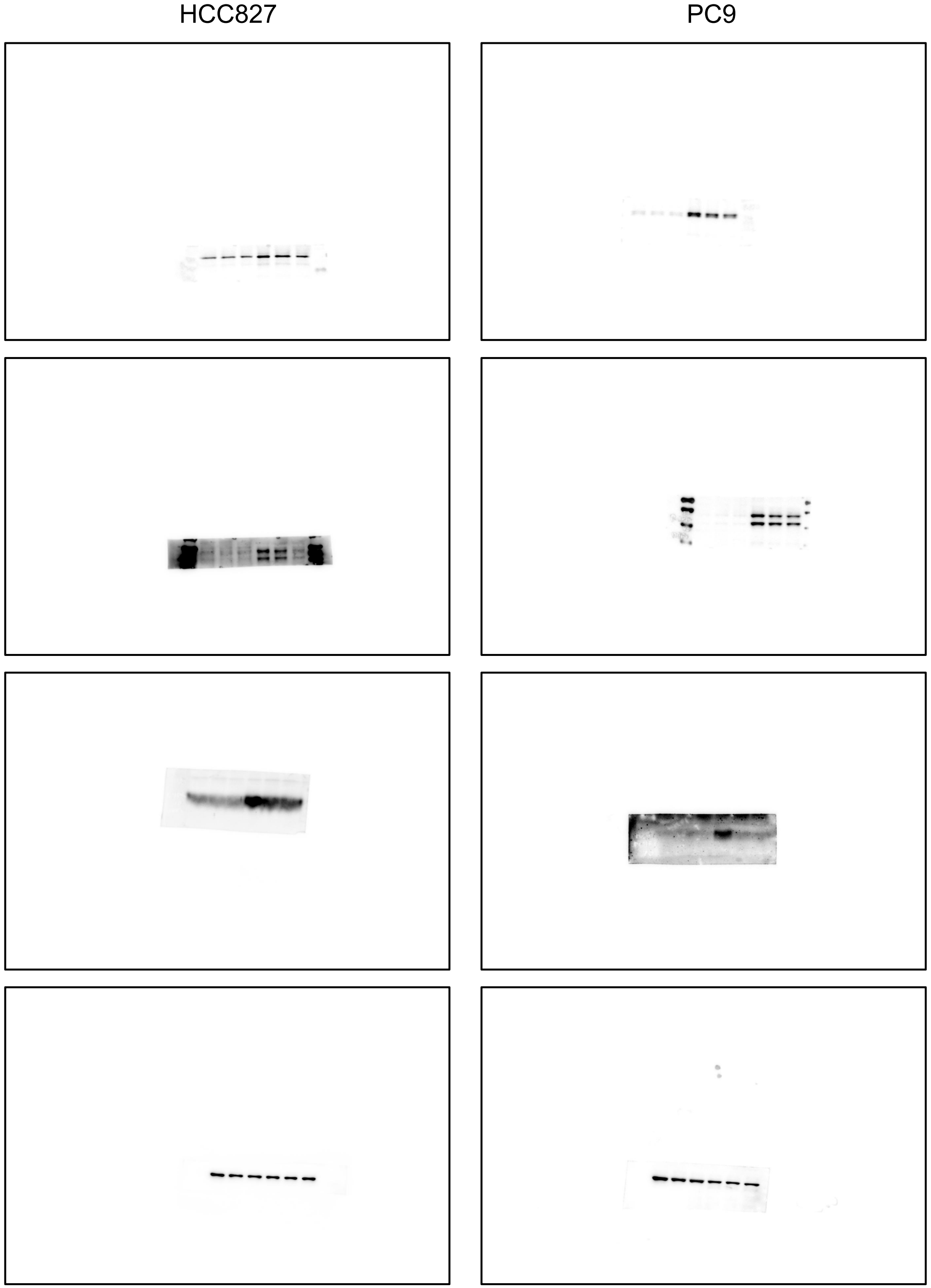

Fig.5L

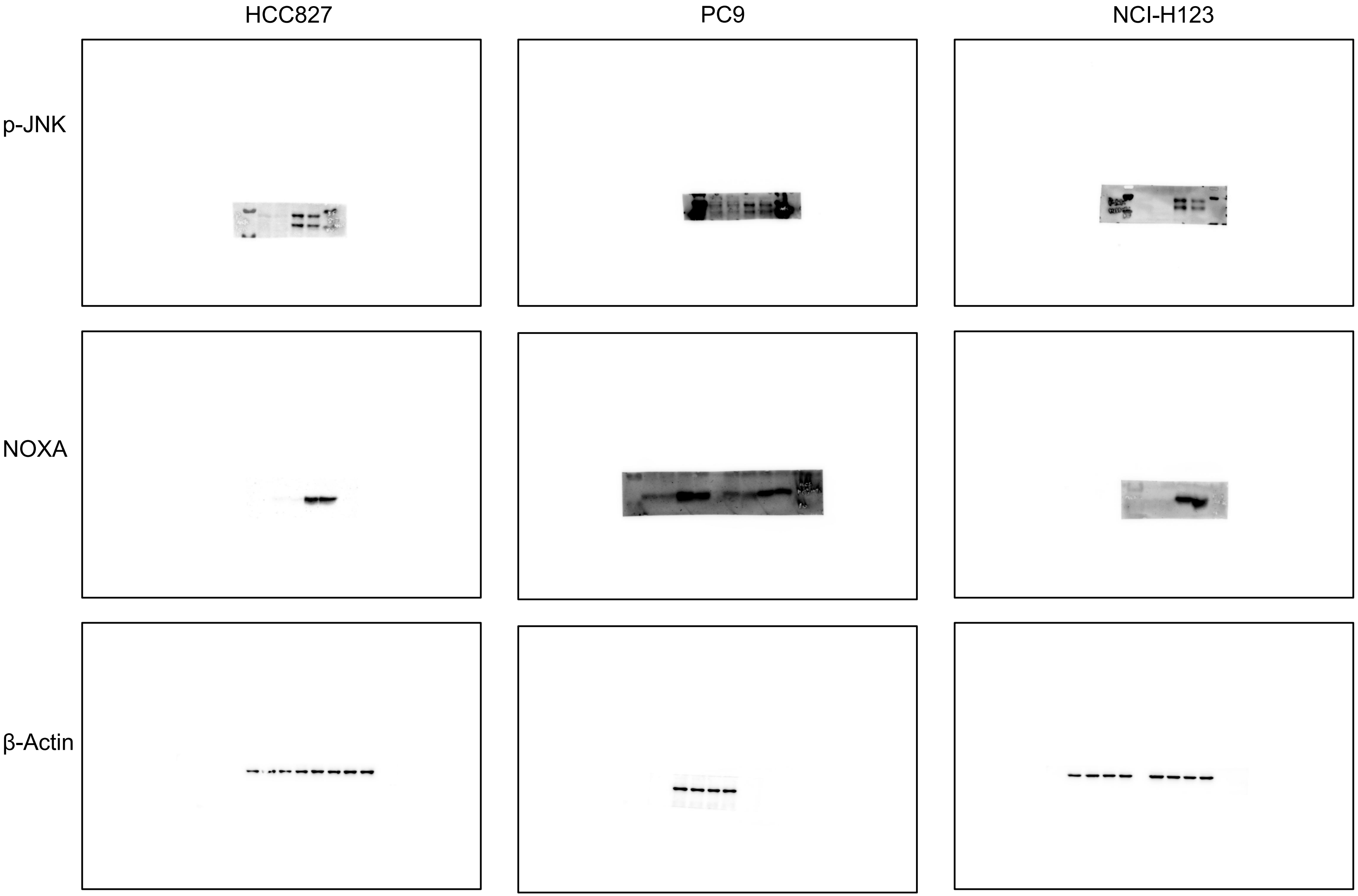

Fig.5M

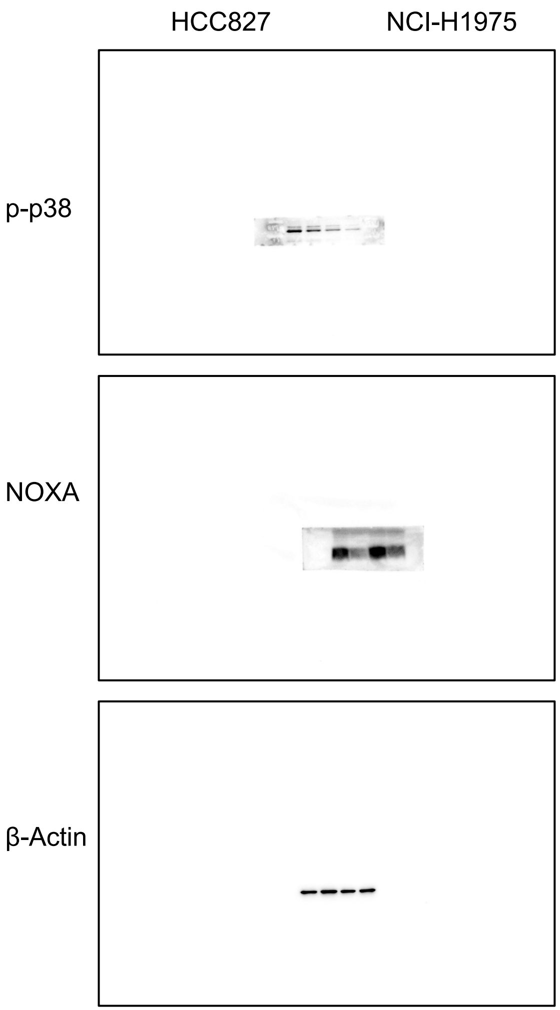
HCC827
NCI-H1975
p-p38
NOXA
β-Actin

Fig.5N

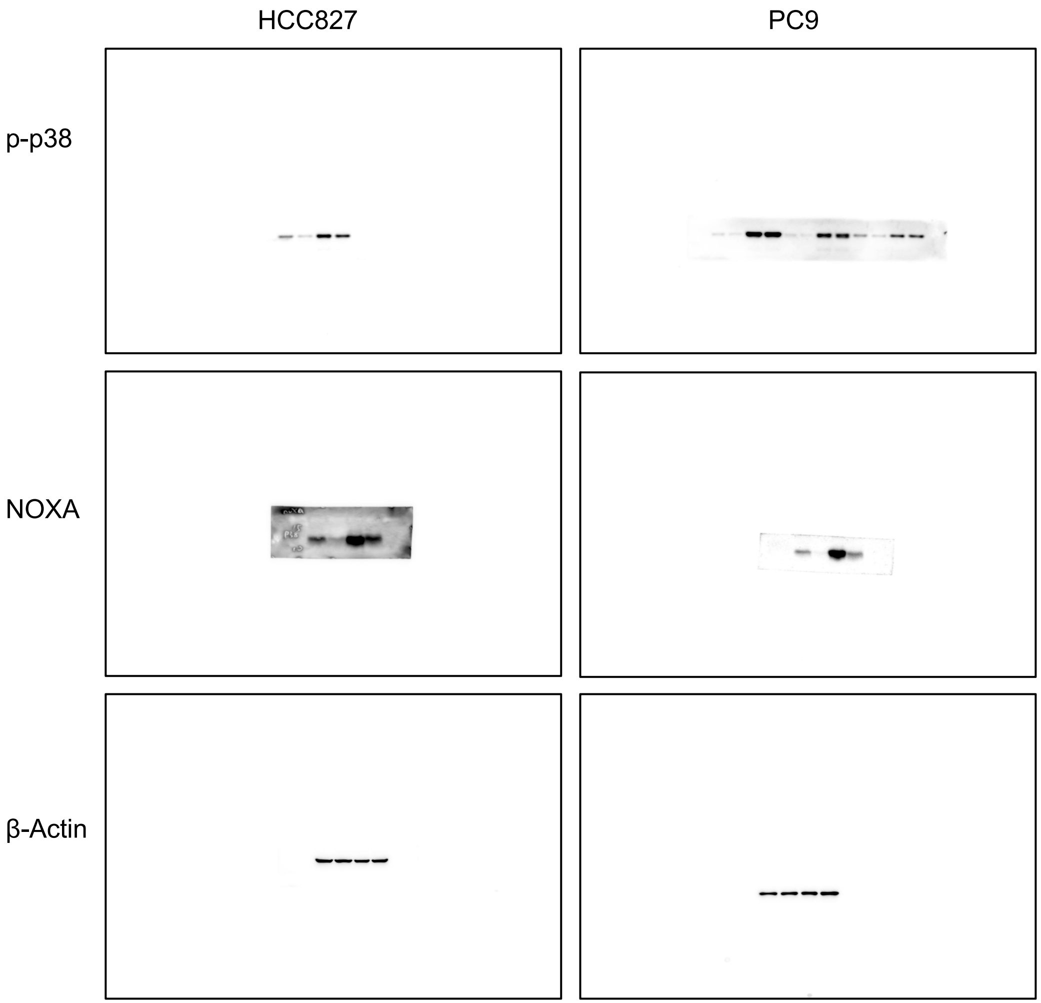
HCC827
PC9
p-p38
NOXA
β-Actin

Fig.6B

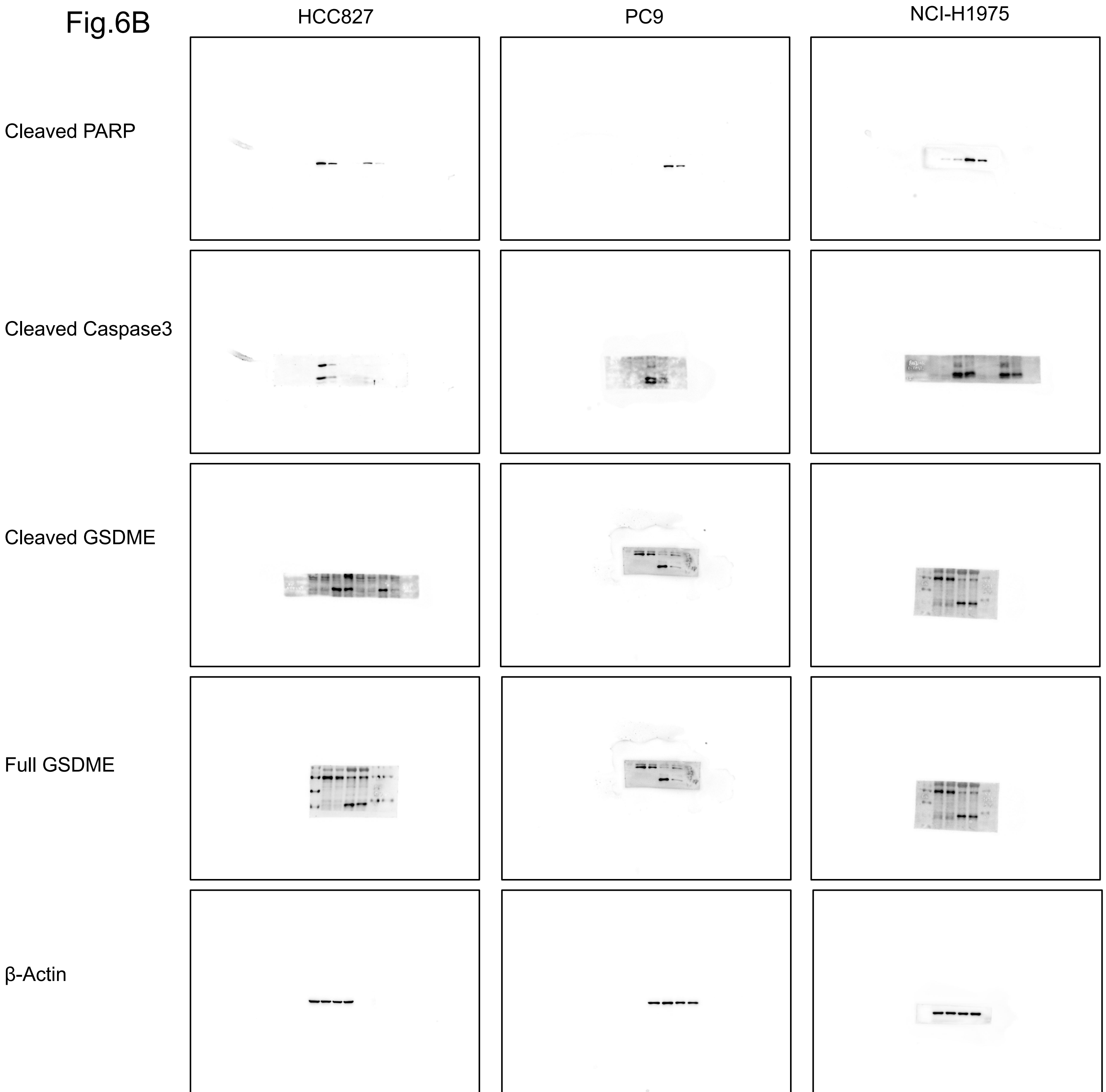

HCC827
PC9
NCI-H1975
Cleaved PARP
Cleaved Caspase3
Cleaved GSDME
Full GSDME
β-Actin

Fig.7A

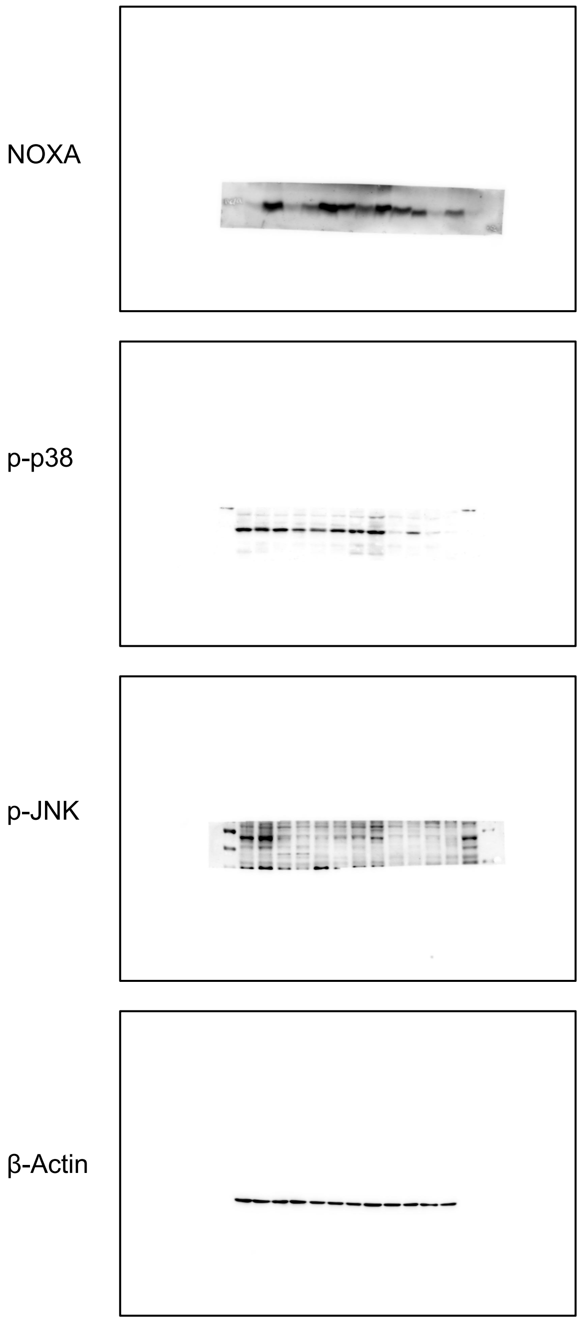

NOXA
p-p38
p-JNK
β-Actin

Fig.8D

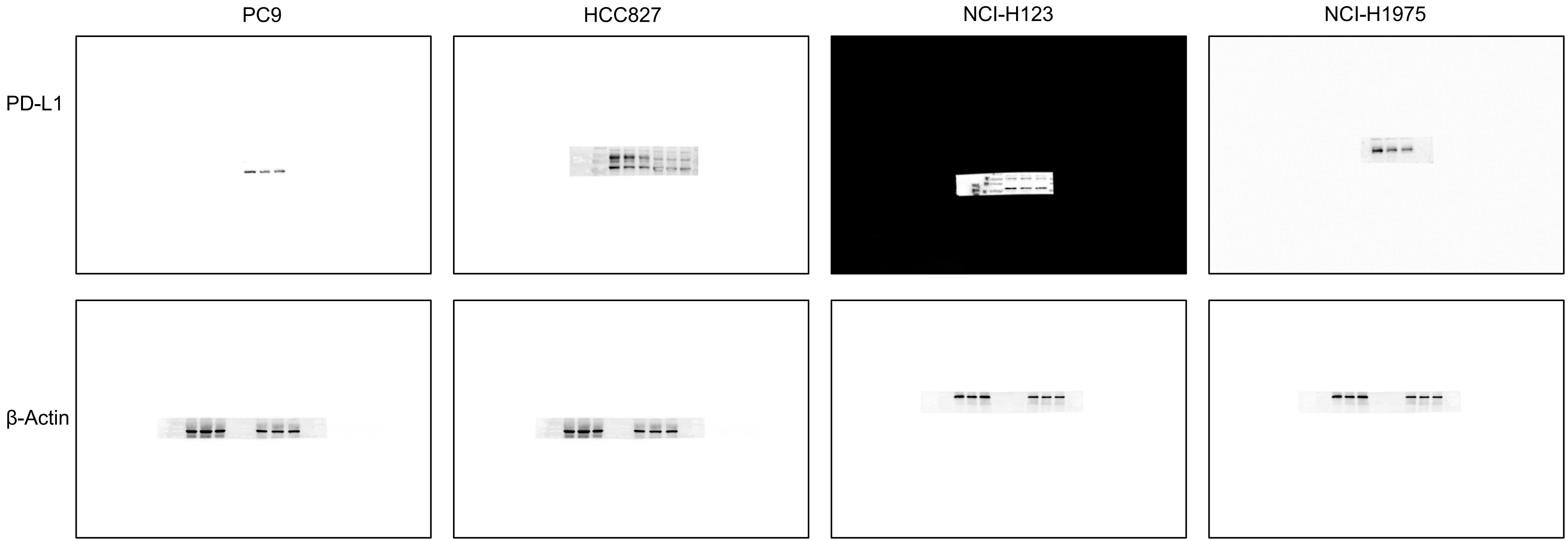

Supplementary Fig.2A

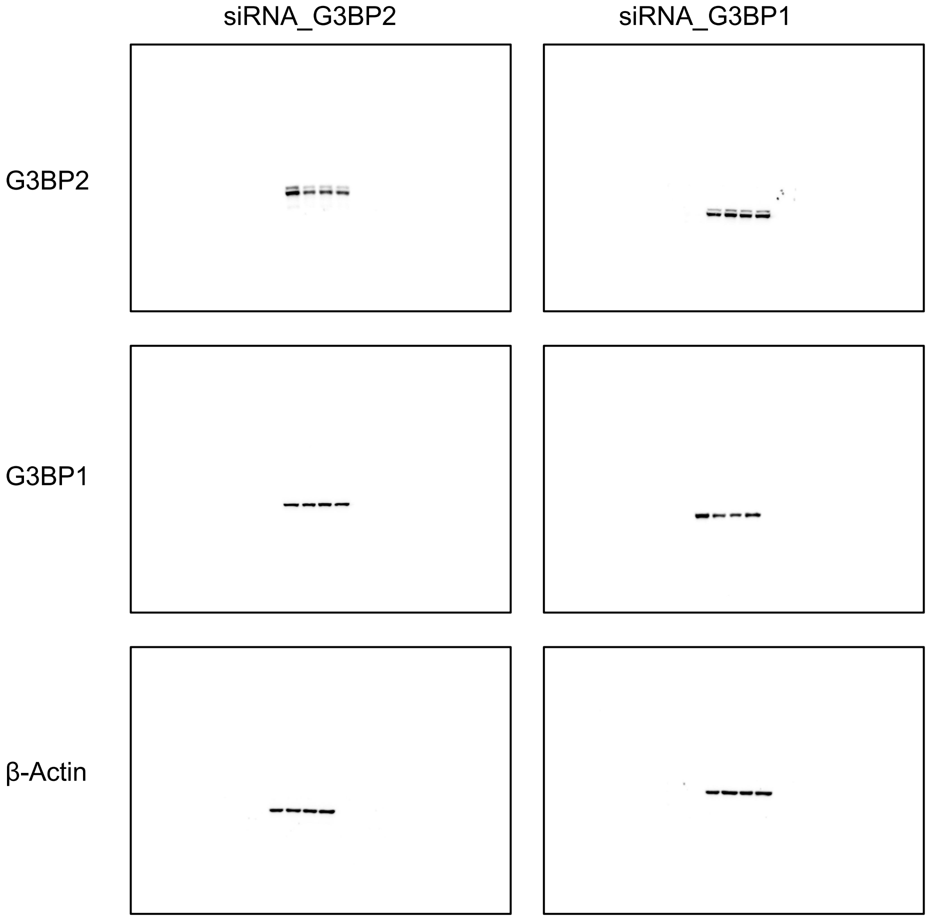

Supplementary Fig.2B

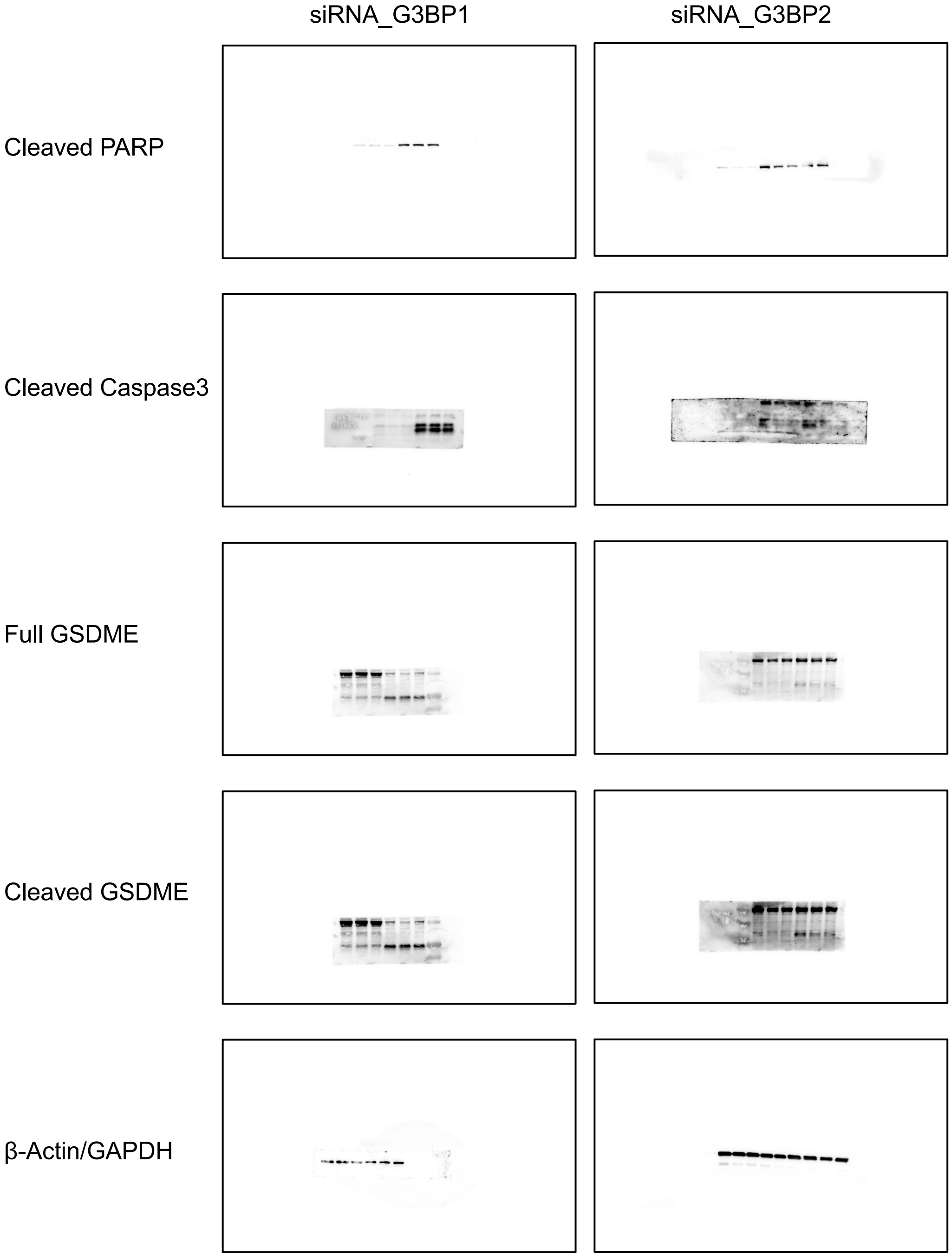

Supplementary Fig.2C

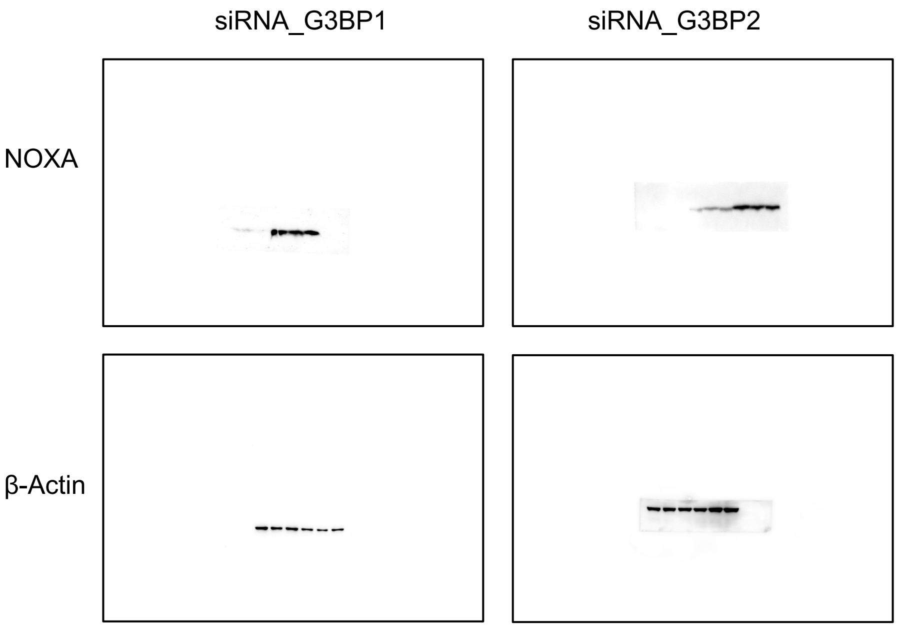

Supplementary Fig.2D

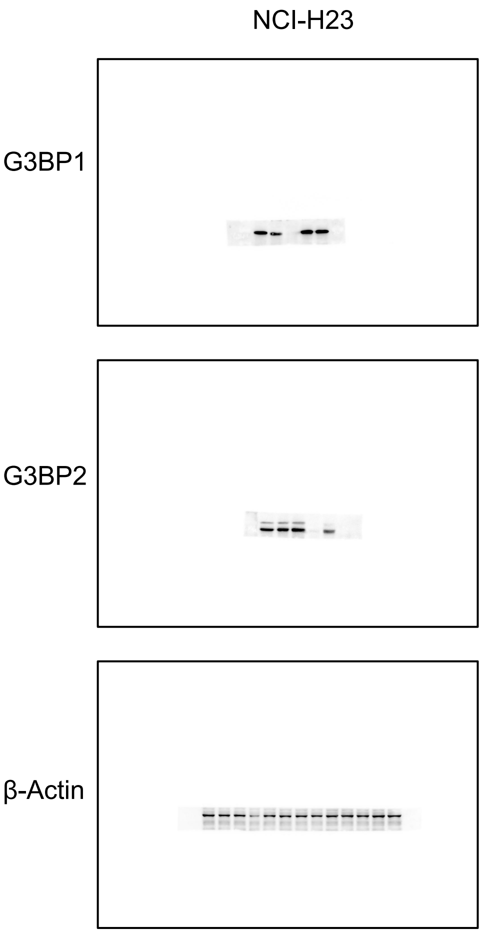

Supplementary Fig.2E

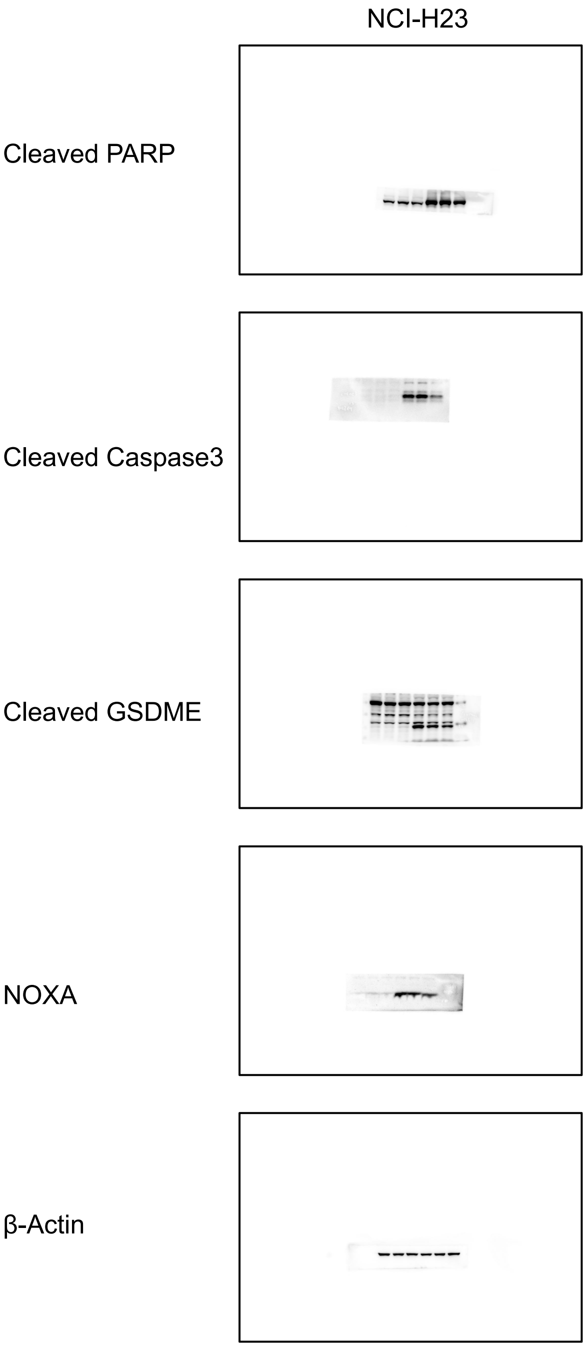

Supplementary Fig.3A

HCC827

p53
Short Exposure

p53
Long Exposure

Cleaved PARP

Cleaved Caspase3

GAPDH

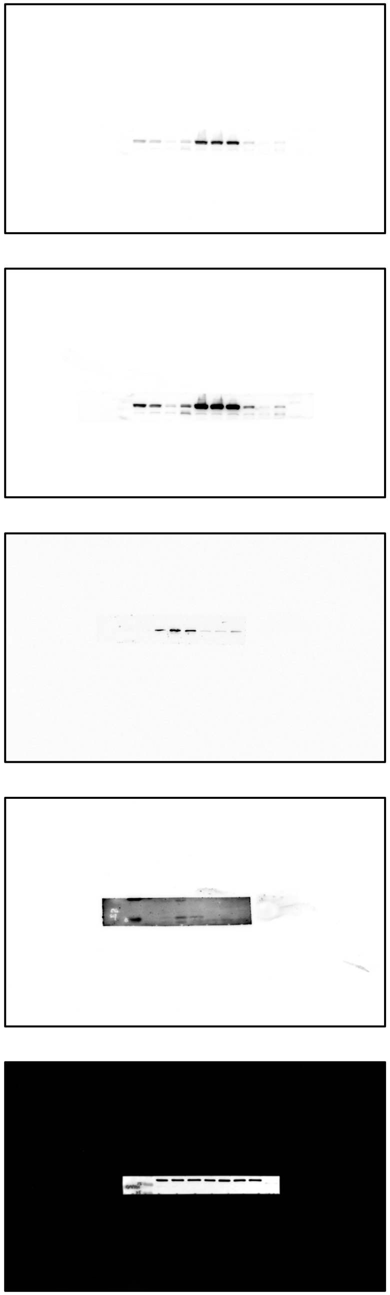

Supplementary Fig.3B

PC9

NCI-H23

p53

Cleaved PARP

Cleaved Caspase3

GAPDH

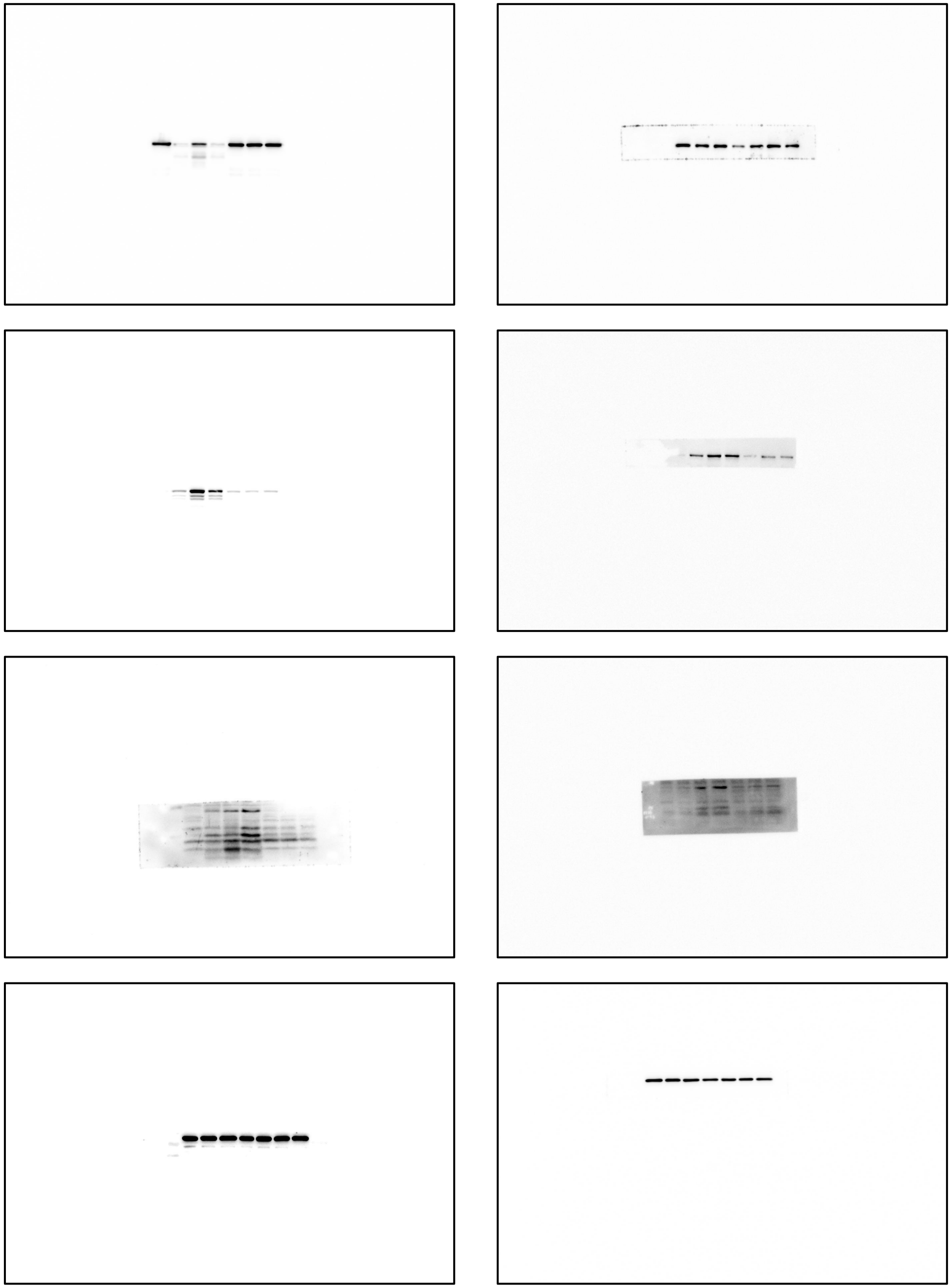

Supplementary Fig.3C

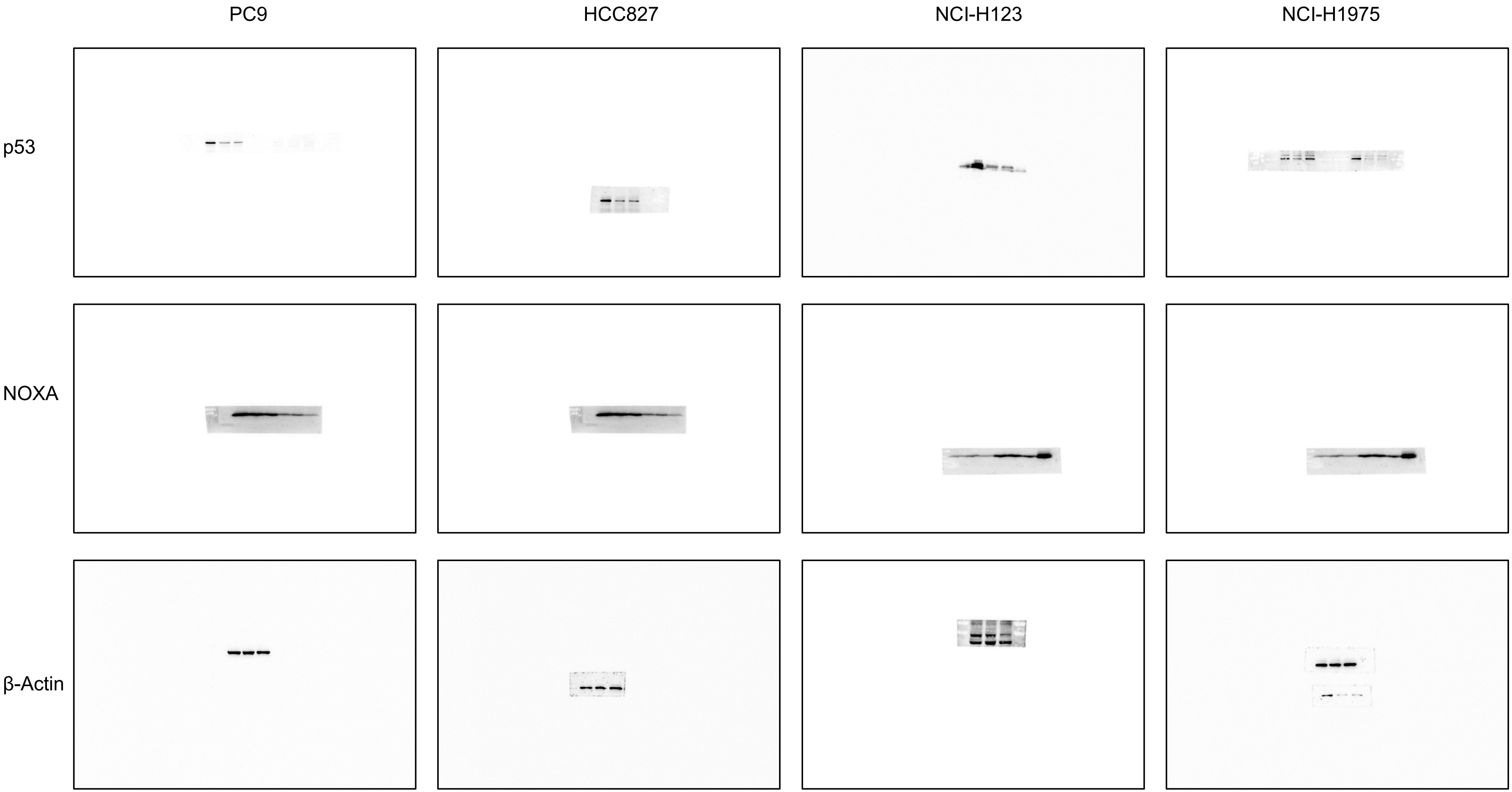

Fig.8B

HCC827 IL-1

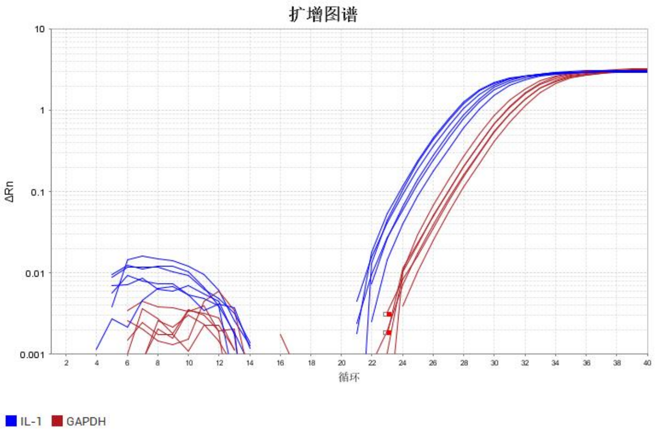

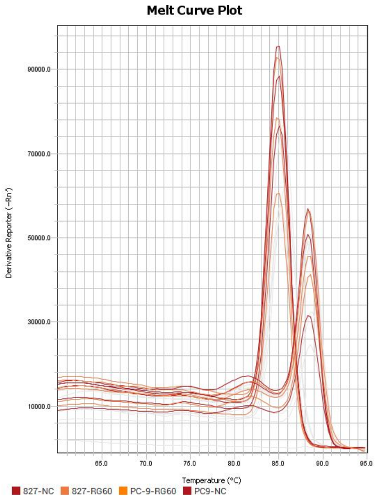

#	Omit	Sample	Target	Cт Mean
5		827-NC	IL-1	26.525
6		827-NC	GAPDH	28.71
7		827-RG60	IL-1	25.602
8		827-RG60	GAPDH	29.419

HCC827 IL-6

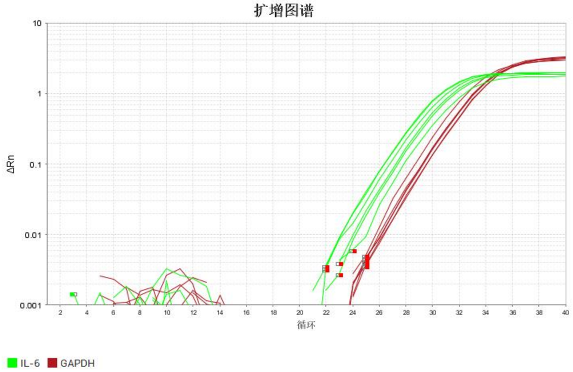

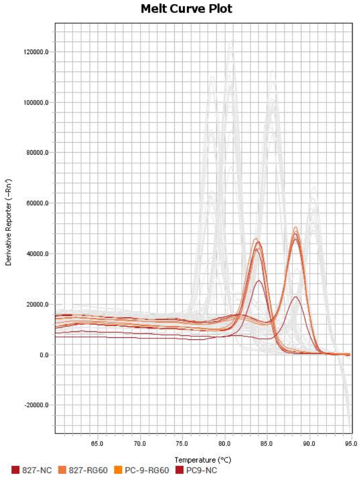

#	Omit	Sample	Target	Cт Mean
1		827-NC	IL-1	28.44
2		827-RG60	IL-1	26.026
3		PC9-NC	IL-1	31.766
4		PC-9-RG60	IL-1	30.089
5		827-NC	IL-6	28.647
6		827-RG60	IL-6	27.665
21		827-NC	GAPDH	31.127
22		827-RG60	GAPDH	31.529

Fig.8B

HCC827 IL-8

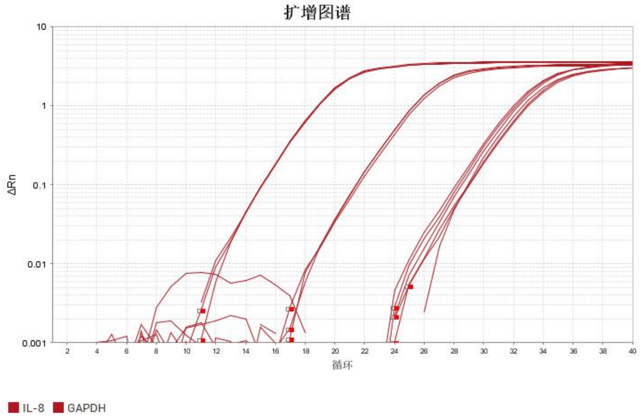

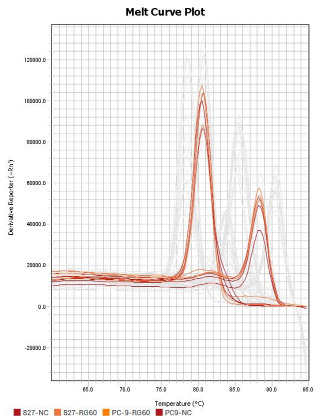

#	Omit	Sample	Target	Cт Mean
7		PC9-NC	IL-6	32.243
8		PC-9-RG60	IL-6	31.401
9		827-NC	IL-8	23.65
10		827-RG60	IL-8	17.162
21		827-NC	GAPDH	30.954
22		827-RG60	GAPDH	30.325

HCC827 IL-11

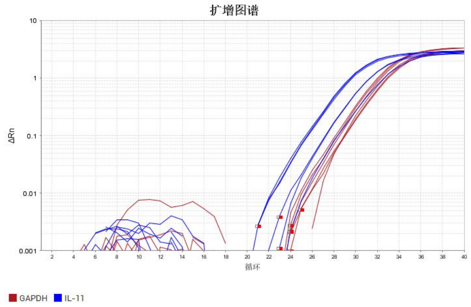

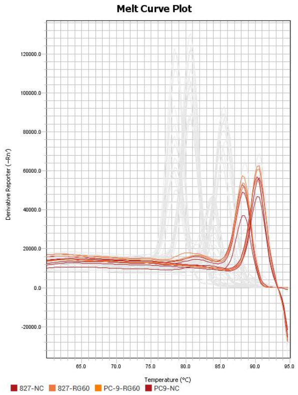

#	Omit	Sample	Target	Cт Mean
8		PC-9-RG60	IL-6	31.401
9		827-NC	IL-8	23.65
10		827-RG60	IL-8	17.162
11		PC9-NC	IL-8	26.412
12		PC-9-RG60	IL-8	21.581
13		827-NC	IL-11	29.498
14		827-RG60	IL-11	27.415
21		827-NC	GAPDH	30.954
22		827-RG60	GAPDH	30.325

Fig.8B

HCC827 CXCL2

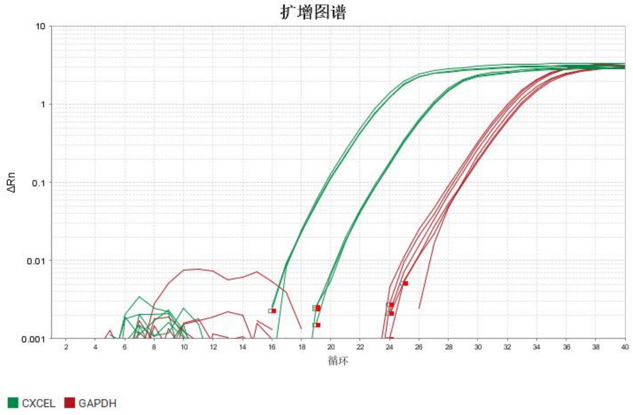

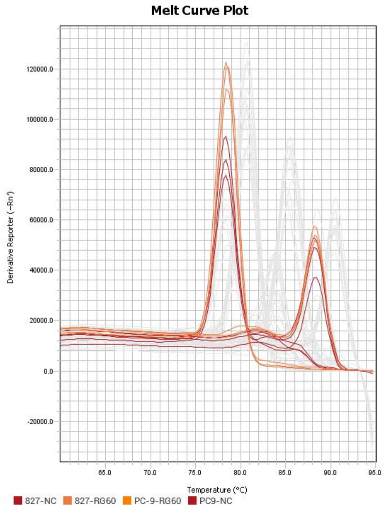

#	Omit	Sample	Target	CT Mean
17		827-NC	CXCEL	24.931
18		827-RG60	CXCEL	21.489
19		PC9-NC	CXCEL	30.972
20		PC-9-RG60	CXCEL	28.084
21		827-NC	GAPDH	30.954
22		827-RG60	GAPDH	30.325

PC9 IL-1

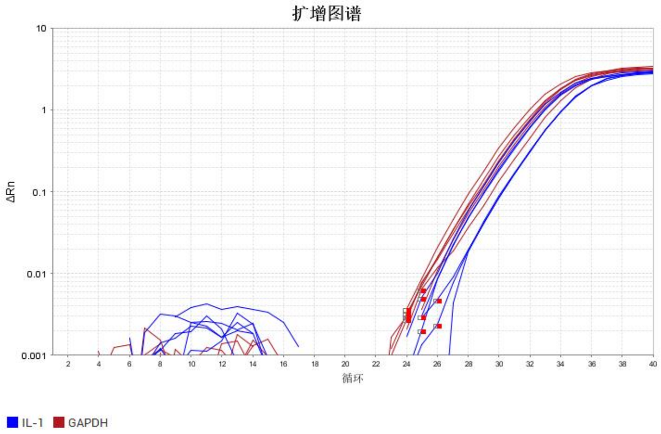

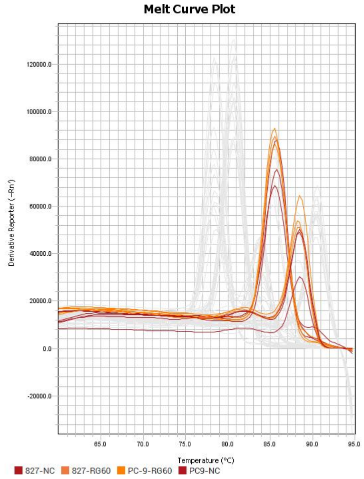

#	Omit	Sample	Target	CT Mean
1		827-NC	IL-1	29.74
2		827-RG60	IL-1	26.975
3		PC9-NC	IL-1	32.158
4		PC-9-RG60	IL-1	30.904
23		PC9-NC	GAPDH	31.011
24		PC-9-RG60	GAPDH	30.516

Fig.8B

PC9 IL-6

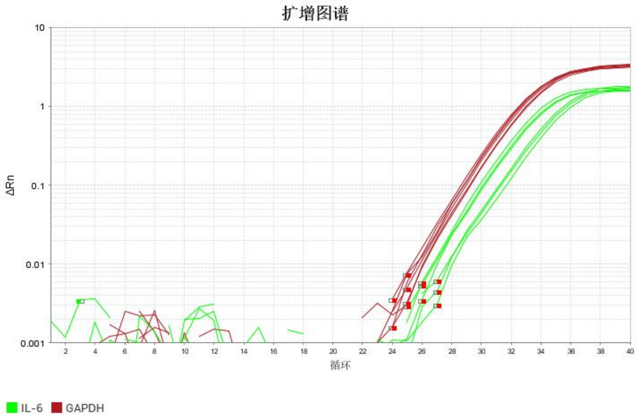

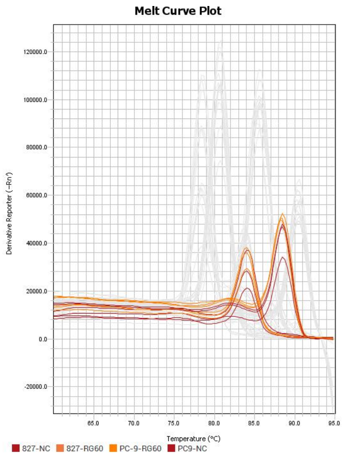

#	Omit	Sample	Target	Cт Mean
1		827-NC	IL-1	28.44
2		827-RG60	IL-1	26.026
3		PC9-NC	IL-1	31.766
4		PC-9-RG60	IL-1	30.089
5		827-NC	IL-6	28.647
6		827-RG60	IL-6	27.665
7		PC9-NC	IL-6	32.587
8		PC-9-RG60	IL-6	31.238
23		PC9-NC	GAPDH	30.974
24		PC-9-RG60	GAPDH	30.982

PC9 IL-8

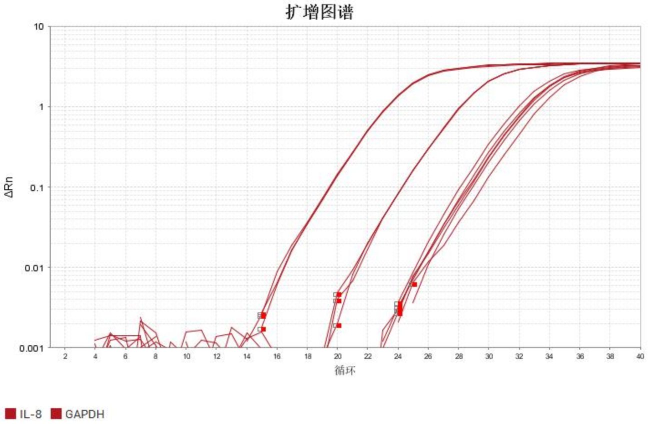

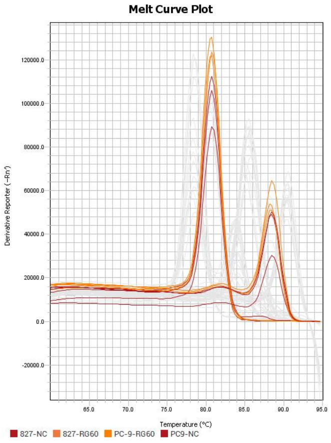

#	Omit	Sample	Target	Cт Mean
7		PC9-NC	IL-6	32.243
8		PC-9-RG60	IL-6	31.401
9		827-NC	IL-8	23.65
10		827-RG60	IL-8	17.162
11		PC9-NC	IL-8	26.412
12		PC-9-RG60	IL-8	21.581
23		PC9-NC	GAPDH	31.011
24		PC-9-RG60	GAPDH	30.516

Fig.8B

PC9 IL-11

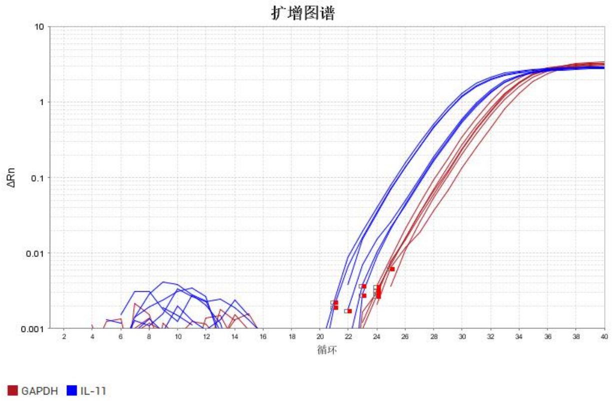

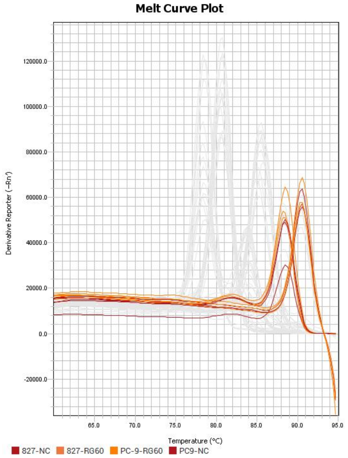

#	Omit	Sample	Target	Cт Mean
7		PC9-NC	IL-6	32.243
8		PC-9-RG60	IL-6	31.401
9		827-NC	IL-8	23.65
10		827-RG60	IL-8	17.162
11		PC9-NC	IL-8	26.412
12		PC-9-RG60	IL-8	21.581
13		827-NC	IL-11	29.498
14		827-RG60	IL-11	27.415
15		PC9-NC	IL-11	28.997
16		PC-9-RG60	IL-11	27.315
23		PC9-NC	GAPDH	31.011
24		PC-9-RG60	GAPDH	30.516

PC9 CXCL-2

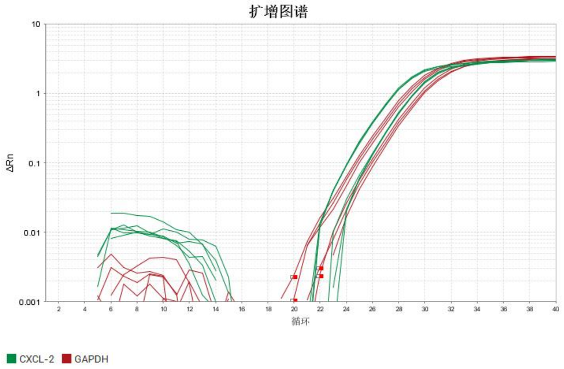

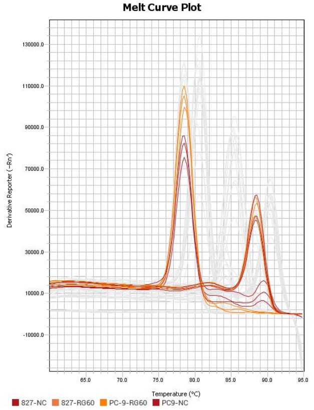

#	Omit	Sample	Target	Cт Mean
19		PC9-NC	CXCL-2	26.841
20		PC-9-RG60	CXCL-2	25.333
21		827-NC	GAPDH	28.433
22		827-RG60	GAPDH	29.081
23		PC9-NC	GAPDH	26.631
24		PC-9-RG60	GAPDH	27.738
